# Single-cell mass cytometry on peripheral cells in Myasthenia Gravis identifies dysregulation of innate immune cells

**DOI:** 10.3389/fimmu.2023.1083218

**Published:** 2023-01-30

**Authors:** Julien Verdier, Odessa-Maud Fayet, Edouard Hemery, Frédérique Truffault, Natalia Pinzón, Sophie Demeret, Anthony Behin, Elie Fadel, Julien Guihaire, Aurélien Corneau, Catherine Blanc, Sonia Berrih-Aknin, Rozen Le Panse

**Affiliations:** ^1^ Sorbonne University, INSERM, Institute of Myology, Center of Research in Myology, Paris, France; ^2^ APHP, Assistance Publique - Hopitaux de Paris, Paris, France; ^3^ AP-HP, Referral Center for Neuromuscular Disorders, Institute of Myology, Pitié-Salpêtrière Hospital, Paris, France; ^4^ Marie Lannelongue Hospital, Paris-Sud University, Le Plessis-Robinson, France; ^5^ Plateforme de Cytométrie de la Pitié-Salpétrière (CyPS), UMS037-PASS, Sorbonne Université-Faculté de Médecine, Paris, France

**Keywords:** autoimmunity, CyTOF mass cytometry, monocytes, γδ T cells, innate lymphoid cells, thymus, Myasthenia Gravis

## Abstract

Myasthenia Gravis (MG) is a neurological autoimmune disease characterized by disabling muscle weaknesses due to anti-acetylcholine receptor (AChR) autoantibodies. To gain insight into immune dysregulation underlying early-onset AChR^+^ MG, we performed an in-depth analysis of peripheral mononuclear blood cells (PBMCs) using mass cytometry. PBMCs from 24 AChR^+^ MG patients without thymoma and 16 controls were stained with a panel of 37 antibodies. Using both unsupervised and supervised approaches, we observed a decrease in monocytes, for all subpopulations: classical, intermediate, and non-classical monocytes. In contrast, an increase in innate lymphoid cells 2 (ILC2s) and CD27^-^ γδ T cells was observed. We further investigated the dysregulations affecting monocytes and γδ T cells in MG. We analyzed CD27^-^ γδ T cells in PBMCs and thymic cells from AChR^+^ MG patients. We detected the increase in CD27^-^ γδ T cells in thymic cells of MG patients suggesting that the inflammatory thymic environment might affect γδ T cell differentiation. To better understand changes that might affect monocytes, we analyzed RNA sequencing data from CD14^+^ PBMCs and showed a global decrease activity of monocytes in MG patients. Next, by flow cytometry, we especially confirmed the decrease affecting non-classical monocytes. In MG, as for other B-cell mediated autoimmune diseases, dysregulations are well known for adaptive immune cells, such as B and T cells. Here, using single-cell mass cytometry, we unraveled unexpected dysregulations for innate immune cells. If these cells are known to be crucial for host defense, our results demonstrated that they could also be involved in autoimmunity.

## Introduction

Myasthenia gravis (MG) is an autoimmune disease caused by autoantibodies against components of the neuromuscular junction that lead to an impairment of muscle contractility and muscle fatigability. The main antigenic target is the acetylcholine receptor (AChR) for about 85% of patients (1). AChR-MG is a complex disease in which the thymus plays a central role. In the early-onset form of the disease that affects mainly young women, thymic follicular hyperplasia is commonly observed. In the late-onset form of the disease, a thymoma is often associated with MG onset (1). In both cases, thymectomy is advised as a treatment for AChR MG patients (2). In addition to thymectomy, usual treatments include acetylcholinesterase inhibitors to prolong signaling at the neuromuscular junction and corticosteroids/immunosuppressors to calm down the immune system (1).

MG is an antibody-mediated autoimmune disease and immune cell dysregulations have been largely studied for CD4 T cells and B cells. Briefly, in AChR MG, there is a consensus to recognize that the autoreactive B cells emerge from the thymus. Recently, Jian et al. demonstrated that clonally related autoreactive B cells mature in the thymus, move to the periphery, and generally decline after thymectomy (3). Changes regarding regulatory B cells (Breg) that possess immunosuppressive functions have also been described in AChR MG patients. A decreased number and an altered functionality of regulatory B cells are observed in untreated AChR MG patients. The proportion of circulating regulatory B cells is restored after thymectomy, as they seem sequestered in the MG thymus (4).

CD4 T cell subpopulations are also largely affected in AChR MG. If the proportion of regulatory CD4^+^ T (Treg) cells does not seem clearly affected in the periphery, a majority of the studies observed a decrease in the immunosuppressive function of Treg cells (5). Truffault et al. showed that functional impairment is more pronounced in thymic than peripheral Treg cells that are phenotypically different (6). An increased proportion of Th17 cells is also observed in AChR MG patients and again seems more pronounced in the thymus (7, 8). The proportion of thymic and circulating follicular helper T (Tfh) cells is also increased in AChR MG. These cells could favor thymic germinal center development but also B-cell activation and antibody production (9–11).

One limitation of studies aiming at characterizing phenotypes of immune cell dysregulations in MG patients is often the lack of sufficiently high numbers of marker combinations enabling unambiguous discrimination between cell populations expressing some common markers. In addition, some immune cell populations such as monocytes, natural killer (NK) cell subsets, or other innate immune cells were not often, or not at all, investigated in MG or other autoimmune diseases (12). Mass cytometry - cytometry by time of flight (CyTOF) - enables the detection of ~40 different markers and provides an unprecedented depth and resolution of immune phenotyping at the single-cell level (13). Recently, CyTOF-based analysis of peripheral blood cells from late-onset AChR MG patients identified two novel dysregulated inflammatory circulating memory T helper cells with a lower percentage of effector memory CD4^+^ T cells expressing GM-CSF and of CD103^+^ CD4 T cells (14).

In this study, our objective was to determine if new immune cell dysregulation could be detected in early-onset MG patients using mass cytometry. To obtain a broad and high-resolution landscape of circulating immune cell dysregulations in early-onset AChR MG patients without thymoma, we analyzed the expression of 37 markers on peripheral blood mononucleated cells (PBMCs). We identified 28 different immune cell populations and found significant differences in MG patients affecting innate immune cells, such as lower percentages of monocyte subpopulations, and higher percentages of type 2 innate lymphoid cells (ILC2) and CD27^-^ γδ T cells and in MG patients. Changes affecting monocytes and γδ T cells were further investigated. Results open new perspectives regarding the implication of these cells in the pathophysiology of MG.

## Materials and methods

### MG patients

Blood was obtained from MG patients before thymectomy or during follow-up consultations, and from sex- and age-matched healthy individuals (Etablissement Français du Sang). PBMCs were isolated by Ficoll density gradient centrifugation (Eurobio), stored in fetal calf serum containing 20% DMSO, and kept in liquid nitrogen until use. All MG patients were early-onset AChR^+^ MG patients with an onset of the disease before 47 years old and without thymoma. Patient details are included in the **Supplementary Tables S1A-E**. Patients were either taking or not anticholinesterase medications. Here, treated patients refer to patients who were thymectomized and treated with corticosteroids. For mass cytometry, 24 treated and untreated AChR MG patients (17-53 years old) (**Supplementary Table 1A**) and 16 adult controls (19-52 years old) were analyzed (**Supplementary Figure 1**). For flow cytometry, 11 untreated AChR MG patients (17-49 years old) (**Supplementary Table 1B**) and 13 adult controls (22-49 years old) were analyzed. For transcriptomic analyses, 5 untreated early-onset AChR MG patients (35-59 years old at sampling) (**Supplementary Table 1C**) and 7 adult controls (23-52 years old) were analyzed (**Supplementary Figure 2**). MG severity score was evaluated at the time of sampling with the MGFA score and/or the quantitative Myasthenia Muscle Score (MMS) based on a scale of 0 to 100 (15).

Thymic biopsies stored at -80°C were obtained from the Marie Lannelongue Surgical Center (Le Plessis-Robinson, France), where early-onset AChR-positive underwent thymectomy, and age/sex-matched non-MG adults had cardiovascular surgery. Thymocytes were isolated from thymuses by mechanical dissociation of fresh thymic tissue, stored in fetal calf serum containing 20% DMSO, and kept in liquid nitrogen until use. Thymic cells were recovered from 8 untreated AChR MG patients (12-34 years old) (**Supplementary Table 1D**) and 9 adult controls (13-33 years old). mRNA was extracted from 10 untreated AChR MG patients (22-35 years old), 6 adult controls (15-37 years old), and 6 infant controls (3-12 months old) (**Supplementary Table 1E**).

These investigations were approved by the local Ethics committee (Comité consultatif de protection des personnes), Ile-de-France VII (Kremlin-Bicêtre, France). The relevant authorization numbers are ID RCB 2006-A00164-47 and 2010-A00250-39.

### Cell staining for mass cytometry

Frozen PBMC cryotubes were thawed and immediately washed twice in pre-warmed RPMI 1640 GlutaMAX™ supplemented with 10% fetal calf serum. Cells were incubated with a viability dye (Cell-ID™ Cisplatin, Fluidigm) washed extensively, and 3.10^6^ cells were stained for extracellular antigens (**Supplementary Table 2A**) in 50µL Maxpar Cell Staining Buffer (Fluidigm) during 1h at 4°C. Cells were fixed for 1h at 4°C and permeabilized for 15mn at 4°C using the Transcription Factor Phospho Buffer Set (BD Bioscience). In a pilot experiment, we observed no differences with or without FcR blocking reagent for cell surface or intracellular markers and did not use it thereafter. Cells were patient-wise barcoded using the Cell-ID™ 20-Plex Pd Barcoding Kit (Fluidigm) for 1h at room temperature and pooled before staining for intracellular antigens (**Supplementary Table 2A**) for 1h at room temperature. Cell DNA was then stained using Cell-ID™ Intercalator-Ir (Fluidigm) in Maxpar H_2_O containing 2% paraformaldehyde overnight at 4°C, frozen at -80°C using a cryopreservation box. Cells were thawed, supplemented with EQ™ Four Element Calibration Beads (Fluidigm) (16), and acquired on a Helios™ mass cytometer (Fluidigm). Files were concatenated, normalized, and debarcoded using the CyTOF software v6.7.1014 (separation cutoff: 0.1, Mahalanobis cutoff: 15) (**Supplementary Figure 3**).

We phenotyped healthy control individuals and treated, untreated MG patients in two different experimental cohorts (two datasets). To reduce bias due to shifts of the machine sensitivity with time, samples were barcoded within each acquired dataset, and we included one common donor in the two experimental cohorts to monitor the inter-experimental batch effect.

### Cell staining for flow cytometry

PBMCs or thymic cells were stained using the LIVE/DEAD™ Fixable Dead Cell Stain Kit (**Supplementary Table 2B**) for 30 min at 4°C in PBS. Cells were washed, split in two, and labeled with two distinct antibody panels for γδ T cell or monocyte investigations (**Supplementary Table 2B**) for 30 min at 4°C. Flow cytometry was performed on a Cytoflex S and data were analyzed using CytExpert (Beckman Coulter).

### RNA extraction and RT-PCR

Total RNA was extracted from thymic biopsies with the mirVana miRNA Isolation Kit (ThermoFisher Scientific). Biopsies were lysed in the Lysis/Binding buffer (mirVana kit) with the FastPrep FP120 instrument (Qbiogen). RNA quality was assessed on a Bioanalyzer 2100 (Agilent Technologies).

mRNAs were retro-transcribed from 1µg of total RNA using the Reverse Transcriptase AMV kit (Roche) and qPCR experiments were carried out using LightCycler 480 SYBR Green Master Mix (Roche). qPCR cycle conditions were: 1 cycle of polymerase activation and denaturation at 95°C for 10 minutes, 45 cycles of amplification at 95°C for 10 seconds, 60-64°C for 1 minute, and 72°C for 12-14 seconds. Primer sequences are listed in **Supplementary Table 2C**.

### CyTOF data analyses

Mass cytometry data were gated in Cytobank to exclude pollutants and select single live immune cells, non-transformed unscaled values were exported and compensated using the spillover matrix generated with the CATALYST R/Bioconductor package and the non-negative linear square method (https://github.com/mb3152/nonnegfac) before arcsinh transformation (scale argument: 5). Dimensionality reduction was performed with UMAP (https://umap-learn.readthedocs.io/en/latest/) and clustering with k-means or Phenograph (https://github.com/jacoblevine/PhenoGraph).

### Transcriptomic analysis

We used CD14 monocyte gene expression data already published (17) and available on GEO (GSE85649). Briefly, blood samples from MG and healthy females were collected, and subjects were tested for autoantibodies against AChR. RNA was purified from sorted CD14^+^ cells, and after quality control, hybridized to Illumina HumanHT-12 v4 (BD-103-0204 Illumina). Intensity values were background-subtracted using GenomeStudio software. In the MG group, we included only AChR MG patients and excluded one thymoma MG patient and patients receiving immunosuppressive drug therapy at the moment of sampling that could affect gene expression in monocytes (18). We also excluded healthy females who had a twin with MG and tested positive for auto-antibodies against AChR. In the current research, 5 AChR MG and 7 healthy samples were selected for further analysis.

Linear Models for Microarray Data (limma) package (version 3.50.3) was used to assess differential expression values, after loess normalization and filtering of negative probes < 6.5. Genes with adjusted p-value<0.05 were regarded as differentially expressed genes (DEGs, **Supplementary Table 3**). Factoextra package (version 1.0.7) was used for dimensionality reduction and PCA visualization of the 200 most variable genes. The package gplots_3.1.3 was used for standard plots such as heatmap.

We used clusterProfiler v4.2.0 package for all enrichment analyses. From the list of differentially expressed genes, enrichment of known biological functions was detected using Over Representation Analysis (ORA) (19) using all expressed genes as universe. To detect more subtle enrichment signals, Gene Set Enrichment Analysis (GSEA) (20) was performed on the entire gene set, with a minimum size of gene set accepted of 10. All collections available in the Molecular Signatures Database (http://www.gsea-msigdb.org/gsea/msigdb/collections.jsp) were queried. Redundant terms were manually removed, and a summary of the most interesting terms was plotted. The gene-term network was plotted using the cnetplot function.

### Statistical analysis

Statistical analyses were performed using the SciPy library for CyTOF analyses or GraphPad Prism 9 software. Comparisons were performed using the Mann-Whitney U test (two-tailed) for unpaired, non-parametric analyses, for two-by-two comparisons, or the one-way analysis of variance (ANOVA) test for unpaired and non-parametric analyses for multiple comparisons (the Kruskal-Wallis test with Dunn’s multiple comparisons test). For correlation analyses, the Spearman’s rank-order correlation, a non-parametric test was used. Details are given in figure legends.

## Results

### Identification of circulating immune cells by mass cytometry

We performed a mass cytometry analysis of PBMCs from 24 patients with MG patients as well as 16 sex- and aged-matched healthy controls in two datasets (**Supplementary Figure 1**). We stained cells with an antibody panel that spanned major immune cell populations expected to be found in PBMCs (**Supplementary Figure 1** and **Table S2A**). Files were de-barcoded and one control was excluded due to abnormally low percentages of immune cells (**Supplementary Figure 3A**). CD45^+^ live single-cells were gated (**Supplementary Figure 3B**) and non-negative least squares (NNLS) compensation was applied to samples (**Supplementary Figure 3C, D**) before down-sampling and downstream analyses (**Supplementary Figure 3E**).

**Figure 1:**
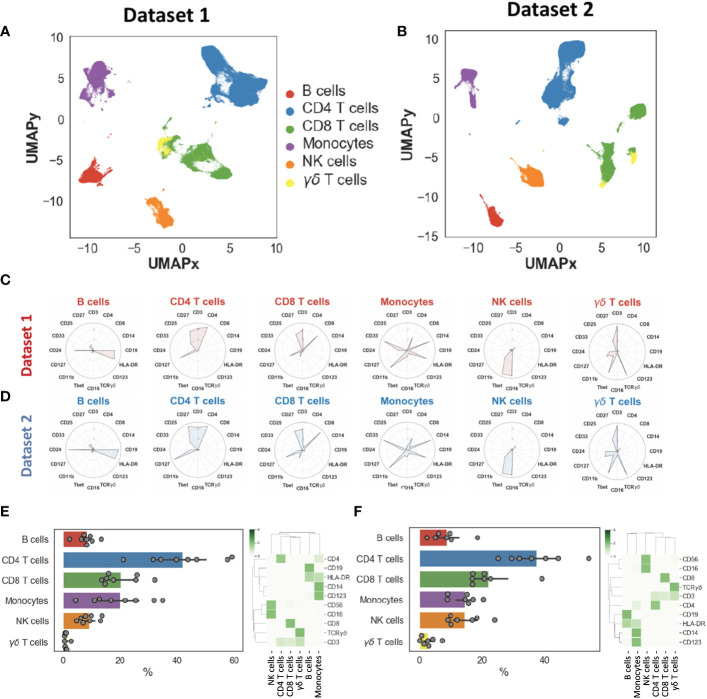
Landscape of major immune cell populations. Dimensionality reduction (UMAP) and annotation of main populations in datasets 1 and 2 **(A–B)**. Mean metal intensities used to identify the main populations for datasets 1 and 2 **(C–D)**. Population percentages and heatmaps illustrating main population phenotypes in datasets 1 **(E)** and dataset 2 **(F)**.

We generated two-dimensional maps of the immune landscape using the dimensionality reduction algorithm UMAP and we performed unsupervised clustering using both k-means and the Phenograph algorithm, which computes phenotypic proximity of cells in a high-dimensionality space. In the first step, we identified the main circulating immune cell populations, including CD4^+^ and CD8^+^ T cells, B cells, NK cells, γδ T cells, monocytes, and NK cells. Cell populations had similar and overlapping phenotypes in both datasets (**Figures 1A–D**). We did not find significantly different percentages between the two datasets, except for NK cells that we could not explain (**Supplementary Figure 4C**). Cell populations displayed expected phenotypes and percentages (**Figures 1E–F**): CD4^+^ T cells= 41.1 ± 13.0%, CD8^+^ T cells= 22.3 ± 7.6%, B cells= 8.3 ± 3.9%, monocytes= 14.0 ± 7.8%, γδ T cells= 2.5 ± 2.9% and NK cells= 11.2 ± 7.5% (these percentages took into account the two datasets).

**Figure 2:**
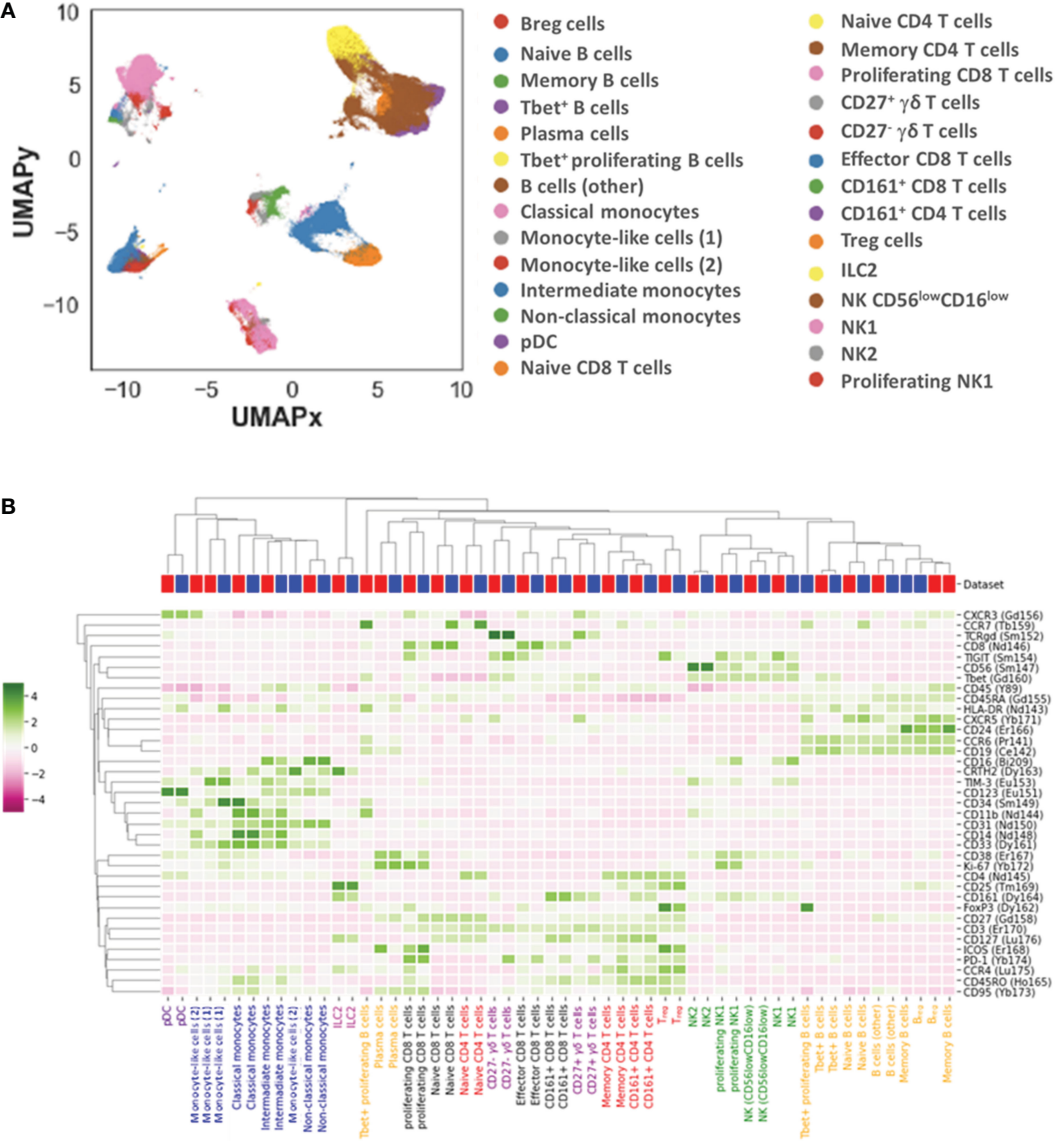
Landscape of immune cell subpopulations. Dimensionality reduction (UMAP) and annotation of subpopulations combining datasets 1 and 2 **(A)**. Heatmap illustrating immune cell subpopulation phenotypes in datasets 1 and 2 **(B)**.

We further identified 28 distinct cell subpopulations (**Figure 2A, B**): 7 for B cells (naïve, memory, plasma and Tbet^+^, Tbet^+^ proliferating B cells, Breg and other B cells), 4 for CD4^+^ T cells (naïve, memory, CD161^+^ and regulatory (Treg) CD4^+^ T cells), 4 for CD8^+^ T cells (naïve, memory, proliferating and CD161^+^ CD8^+^ T cells), 3 for monocytes (classical, intermediate, non-classical monocytes), 4 for NK cells (NK1, proliferating NK1, NK2, and CD56^low^ CD16^low^ NK cells), 2 for γδ T cells (CD27^+^ and CD27^-^ γδ T cells), plasmacytoid dendritic cells (pDCs), type 2 innate lymphoid cells (ILC2) and 2 monocyte-like cells (CD14^low^ CD16^-^ CD33^+^ HLA-DR^low^) that were further classified based on the expression of CD34 and mainly TIM-3.

The two datasets displayed overall equivalent cell population percentages and phenotypes, indicating the possibility to combine these datasets and search for pathophysiological changes in MG patients as compared to healthy donors.

### Differences between controls and MG patients

**Figure 3:**
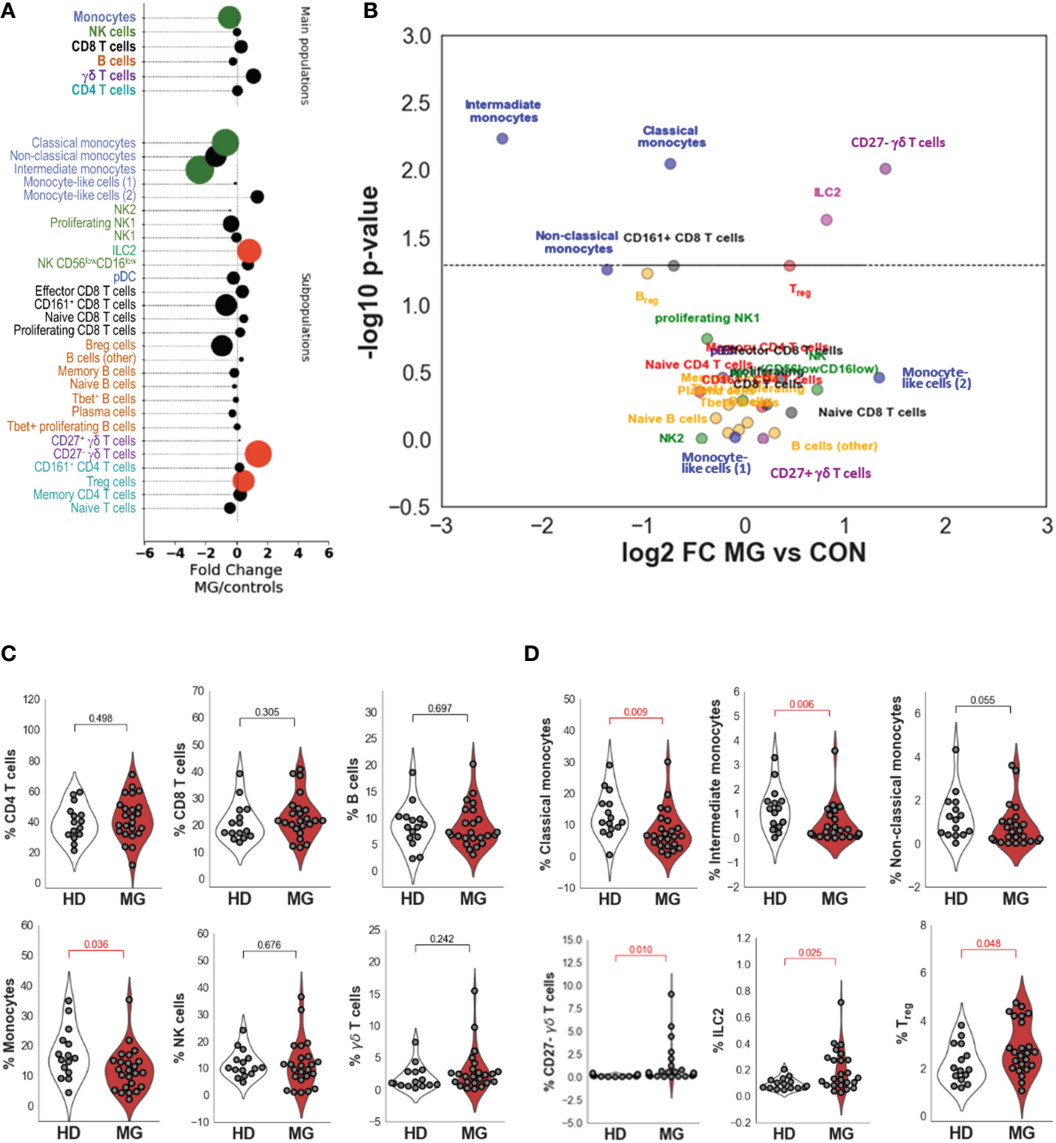
Differences between controls and MG patients. Bubble charts indicating log-2 median fold change of cell percentages between MG patients and controls for main cell populations and subpopulations. Bubble size is proportional to statistical significance and colorized in black if the difference is not significant, in green if the fold change is significantly lower in MG patients than in controls and in red if the fold change is significantly higher in MG patients than in controls **(A)**. Volcano plot indicating -log10 p values on the y-axis and log2 fold change between MG patients and healthy controls on the x-axis for the different annotated subpopulations **(B)**. Distribution of cell population percentages and differences between healthy donors (HD) and MG patients (MG) for the main cell populations (CD4, CD8, and γδ T cells, B cells, NK cells, and monocytes) **(C)** and significant differences observed for cell subpopulations (Classical, intermediate and non-classical monocytes, CD27^-^ γδ T cells, ILC2 and Treg cells) **(D)**. p values were determined by the Mann-Whitney test (two-tailed) for non-parametric analyses.

Comparing all MG patients to controls (**Figures 3A, B** and **Table 1**), we did not observe significantly different percentages of CD4^+^ and CD8^+^ T cells, and B cells between controls and MG patients (**Figure 3C** and **Table 1**). However, MG patients displayed significantly reduced percentages of monocytes (FC=0.72, pval=0.036, **Figure 3C**) compared to controls characterized by a decrease in all three monocytes subpopulations: classical (FC=0.6, pval=0.009), intermediate (FC=0.19, pval=0.006) and non-classical (FC=0.39, pval=0.055) monocytes (**Figure 3D**). We found a slight increase in Treg cells (FC=1.37, pval=0.048, **Figure 3D**) and a much higher and significant increase in ILC2 (FC=1.76, pval=0.025, **Figure 3D**) and in CD27^-^ γδ T cells (FC=2.65, pval=0.010) (**Figures 3A–D** and **Table 1**). At a less significant level with p-values between 0.05 and 0.06, we detected a slight decrease in Breg cells (FC=0.51, pval=0.055, **Table 1**) and CD8^+^CD161^+^ cells (FC=0.62, pval=0.051, **Table 1**).

**Table 1:** Comparison of proportion of cell subpopulations between different subgroups of patients.

Cell subpopulation definition	All MG vs HD		Untreated MG vs HD		Treated MG vs HD		Untreated vs treated MG		Correlation between the score severity and the proportion of cells for MG patients		Population classification
	FC	pval	FC	pval	FC	pval	FC	pval	r	correl. pval	
CD4 T cells	1.04	0.498	1.04	0.726	1.09	0.371	0.96	0.689	0.237	0.266	Main
CD8 T cells	1.22	0.305	1.24	0.371	1.30	0.371	0.96	0.936	0.178	0.406	Main
B cells	0.85	0.697	0.96	0.846	0.69	0.129	1.39	0.093	0.015	0.945	Main
Monocytes	0.72	0.036	0.70	0.150	0.72	0.111	0.98	0.936	-0.058	0.786	Main
NK cells	1.01	0.676	1.17	0.293	0.19	0.047	6.13	0.093	-0.578	0.003	Main
γδ T cells	2.14	0.242	2.58	0.111	1.27	0.559	2.03	0.128	-0.213	0.317	Main
CD4 T cells (naive)	0.74	0.444	0.67	0.460	1.05	0.613	0.64	0.378	0.244	0.252	Subpopulation
CD4 T cells (memory)	1.17	0.334	1.25	0.460	0.95	0.508	1.31	0.230	-0.010	0.961	Subpopulation
CD4 T cells (Treg)	1.37	0.048	1.81	0.011	1.25	0.293	1.46	0.066	-0.074	0.729	Subpopulation
CD4 T cells (CD161+)	1.13	0.593	1.46	0.259	1.09	0.613	1.34	0.471	-0.176	0.411	Subpopulation
CD8 T cells (naive)	1.38	0.634	1.65	0.559	1.71	0.228	0.96	0.936	0.477	0.018	Subpopulation
CD8 T cells (effector)	1.29	0.348	1.50	0.228	1.13	0.969	1.34	0.689	0.011	0.960	Subpopulation
CD8 T cells (proliferating)	1.17	0.573	1.84	0.371	1.09	0.726	1.69	0.689	-0.315	0.134	Subpopulation
CD8 T cells (CD161^+^)	0.62	0.051	0.59	0.080	0.51	0.094	1.17	0.810	-0.168	0.433	Subpopulation
B cells (naive)	0.90	0.897	0.98	0.508	0.39	0.056	2.48	0.031	0.047	0.827	Subpopulation
B cells (memory)	0.90	0.573	0.90	0.969	1.51	0.173	0.60	0.173	0.169	0.429	Subpopulation
B cells (nonproliferating Tbet^+^)	0.97	0.829	1.41	0.129	0.81	0.613	1.74	0.066	-0.053	0.807	Subpopulation
B cells (CD27^+^ CD38^-^ CD24^-^ CD95^+^)	1.24	0.897	1.35	0.726	1.88	0.293	0.72	0.230	0.104	0.630	Subpopulation
B cells (CD27^+^ CD38^+^CD24^+^) (Breg)	0.51	0.055	0.49	0.094	0.53	0.094	0.92	0.810	-0.261	0.217	Subpopulation
B cells (plasma cells)	0.82	0.697	0.82	0.846	1.29	0.414	0.64	0.936	-0.072	0.737	Subpopulation
B cells (proliferating Tbet^+^)	1.03	0.773	1.48	0.293	1.40	0.414	1.06	0.936	0.004	0.984	Subpopulation
Monocytes (classical)	0.60	0.009	0.64	0.094	0.71	0.111	0.90	0.936	0.007	0.974	Subpopulation
Monocytes (intermediate)	0.19	0.006	0.15	0.173	0.23	0.056	0.65	0.936	-0.154	0.473	Subpopulation
Monocytes (non-classical)	0.39	0.055	0.61	0.846	1.03	0.669	0.60	0.378	0.111	0.605	Subpopulation
Monocyte-Like cells 1 (CD14^low^ CD16^-^ CD33^+^ HLA-DR^low^ CD34^+^ TIM-3^+^)	2.53	0.334	0.42	0.150	0.67	0.414	0.63	0.471	-0.207	0.332	Subpopulation
Monocyte-Like cells 2 (CD14^low^ CD16^-^ CD33^+^ HLA-DR^low^ CD34^low^ TIM-3^-^)	0.94	0.965	0.72	0.228	0.62	0.129	1.16	0.689	-0.288	0.172	Subpopulation
NK (CD56^low^ CD16^low^)	1.66	0.427	2.66	0.067	0.27	0.293	9.88	0.230	-0.353	0.090	Subpopulation
NK1 (CD56^dim^ CD16^+^)	0.99	0.535	1.24	0.559	0.09	0.027	14.03	0.066	-0.616	0.001	Subpopulation
NK1 (proliferating)	0.78	0.179	1.38	0.460	0.50	0.027	2.79	0.045	-0.511	0.011	Subpopulation
NK2 (CD56^++^ CD16^-^)	0.75	0.988	1.61	0.173	1.38	0.199	1.17	0.936	-0.054	0.802	Subpopulation
ILC2	1.76	0.025	2.06	0.014	2.59	0.293	0.79	0.810	-0.130	0.544	Subpopulation
CD27^-^ γδ T cells	2.65	0.010	2.76	0.047	1.58	0.508	1.74	0.378	-0.194	0.364	Subpopulation
CD27^+^ γδ T cells	1.14	0.988	1.61	0.559	0.17	0.027	9.72	0.066	-0.373	0.073	Subpopulation
pDC	0.87	0.348	0.76	0.228	0.67	0.199	1.14	0.936	-0.317	0.132	Subpopulation

Analysis of the difference between the percentage of the main PBMC populations and PBMC subpopulations obtained by mass cytometry. A fold change (FC) was calculated and the Mann-Whitney test for unpaired, non-parametric analyses was used to determine the pvalue for the following comparisons: All MG patients versus healthy donors (HD), untreated MG patients versus HD, treated MG patients versus HD and untreated versus treated MG patients: . Correlations between cell population percentages and clinical activity scores were calculated using the Spearman rank-order correlation coefficient in the SciPy library.

Collectively, our high-dimensional analysis reveals a disequilibrium of circulating immune cell populations related to innate immunity such as monocytes, ILC2, and CD27^-^ γδ T cells that had not been associated with MG so far to the best of our knowledge.

### Impact of treatments and disease severity

**Figure 4:**
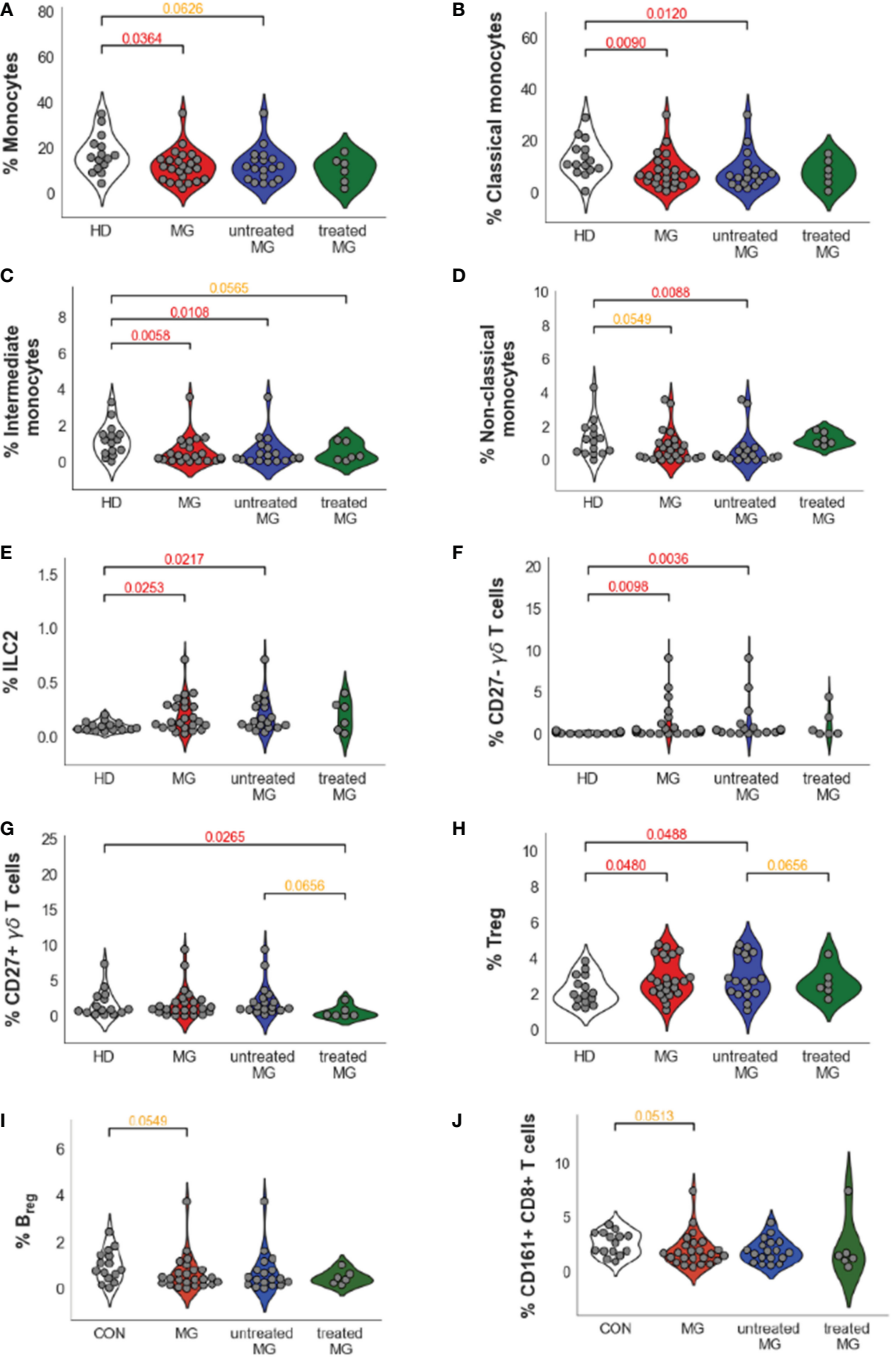
Impact of the treatments on cell percentage for dysregulated cells in AChR MG. Distribution of cell population percentages and differences between healthy donors (HD), all MG patients (MG), and separately for untreated and treated MG patients for all monocytes **(A)**, classical **(B)**, intermediate **(C)**, and non-classical **(D)** monocytes, ILC2 **(E)**, CD27^-^ **(F)** and CD27^+^ **(G)** γδ T cells, Treg **(H)** and Breg **(I)** cells and CD161^+^CD8^+^ T cells **(J)**. p values were determined by the Mann-Whitney test (two-tailed) for non-parametric analyses. p values were indicated when p <0.05 (in red) and 0.1 (in orange).

MG patients in our datasets included both non-thymectomized MG patients that were not treated with corticosteroids (untreated MG patients), and thymectomized MG patients receiving corticosteroids (treated MG patients), and all have various disease severity scores (**Supplementary Figure 1** and **Table S1A**). We analyzed separately all MG (n=24), untreated MG (n=18) and treated MG (n=6) patients versus controls for cell populations that were different between controls and all MG patients with a p-value below 0.1 (**Table 1**): Monocyte subpopulations (**Figures 4A–D**), ILC2 (**Figure 4E**), CD27^-^ but also CD27^+^ γδ T cells (**Figures 4F, G**), Treg cells (**Figure 4H**), Breg cells (**Figure 4I**) and CD161^+^CD8^+^ T cells (**Figure 4J**). As the number of treated MG patients was low, significant differences were not clearly observed when this group was compared to untreated MG patients or controls. Nevertheless, we did not observe much impact of treatments on the percentage of these cell populations except for non-classical monocytes which were clearly increased in the group of treated patients even if non-significantly (**Figure 4D**).

Interestingly, we observed that treatments could affect certain cell populations independently of the MG disease (**Table 1**). Comparing untreated versus treated MG patients, treatments decreased the percentage of CD27^+^ γδ T cells (FC=9.72, pval 0.066) (**Figure 4G**). The treatments affected also B cells by decreasing the percentage of all B cells (FC=1.39, pval=0.093), especially by affecting naïve B cells (FC=2.48, pval=0.031). Similarly, NK cells (FC=6.13, pval=0.093) were decreased in the group of treated MG patients and this was especially due to changes in NK1 cell subpopulations: NK1 CD56^dim^CD16+ (FC=14.03, pval=0.066) and proliferating NK1 (FC=2.79, pval=0.045) (**Table 1**). All these changes due to the treatments were probably independent of the disease as they were also observed when comparing controls and treated MG patients but not controls and untreated MG patients (**Table 1**). Of note, no correlation were observed between percentage of monocytes or CD27^-^ γδ T cells and anti-AChR antibody levels.

Amongst immune cell populations with significantly different percentages in MG patients, none had percentages correlating significantly with disease severity (**Table 1**). However, we observed a statistically significant positive correlation between disease severity and an increase in the percentage of NK cells (r=-0.58, p-value= 0.003), due especially to CD56^dim^CD16^+^ NK cells (r= -0.62, p-value= 0.001) and proliferating NK1 (r= -0.51, p-value= 0.011) (**Table 1**). Conversely, we observed statistically significant negative correlations between disease severity and the percentage of naïve CD8^+^ T cells (r= 0.48, p-value= 0.02) (**Table 1**).

Altogether, we confirmed that the changes observed for MG patients on innate immune cells, i.e. the decrease in monocyte subpopulations and the increase in ILC2 and CD27^-^ γδ T cells, were not different when comparing the group of untreated MG patients with the group of patients treated by thymectomy and corticosteroids. We next decided to investigate further cells that were mostly dysregulated in MG: CD27^-^ γδ T cells and monocytes.

### Increased proportion of CD27- γδ T cells in MG patients: thymic dysregulation

**Figure 5:**
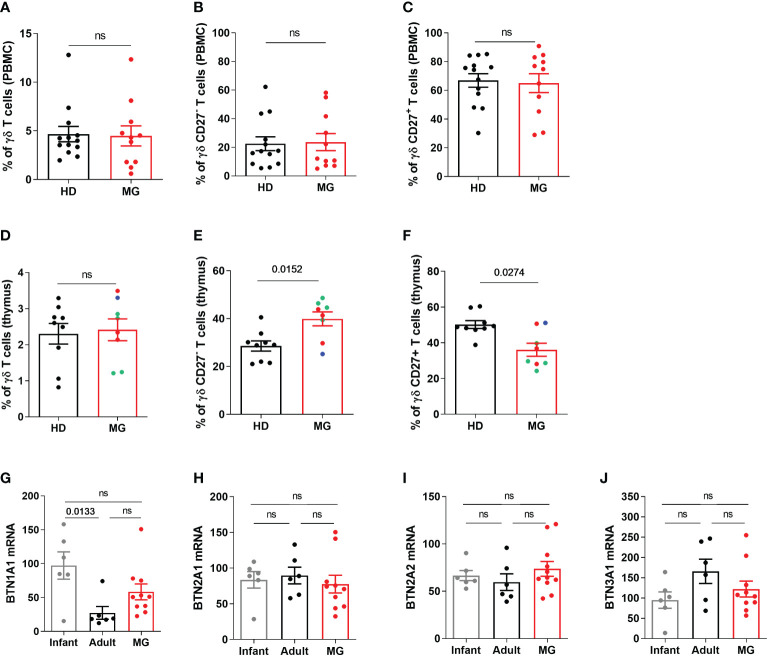
Peripheral and thymic γδ T cells in controls and MG patients. Analysis by flow cytometry of the percentages of γδ T cells **(A, D)**, of CD27^-^ **(B, E)** and CD27^+^ **(C, F)** γδ T cells in PBMCs and thymic cells, respectively, from healthy individuals (HD) or in AChR MG patients (MG). The degree of thymic follicular hyperplasia was indicated: numerous (red dots), few (green dots), and none (bleu dot) germinal centers. RT-PCR analysis for BTN1A1 **(G)**, BTN2A1 **(H)**, BTN2A2 **(I)**, and BTN3A1 **(J)** in the thymus of healthy individuals (infants or adults) and AChR MG patients). p values were determined by the Mann-Whitney test **(A–F)** or the Kruskal-Wallis test with Dunn’s multiple comparisons test for multiple comparisons **(G–J)**.

Unlike αβ T cells, γδ T cells correspond to a minor subset of T lymphocytes, accounting for only 1–5% of circulating lymphocytes (21). Analyzing the percentage of γδ T cells, and CD27^-^ or CD27^+^ γδ T cells by FACS in PBMCs, we did not observe any differences between controls and MG patients for the percentage of γδ T cells (**Figure 5A**) or CD27^-^ or CD27^+^ γδ T cells (**Figures 5B, C**). This difference between CyTOF and FACS results for CD27^-^ γδ T cells could be due to a signal loss by FACS due to the compensations that need to be applied depending on the combination of fluorochromes or to the different cohorts of MG patients. As immune dysregulation is often more pronounced in the thymus of MG patients (6), we analyzed thymic γδ T cells. No change was found regarding the percentage of γδ T cells in the thymus of MG patients (**Figure 5D**) but a significant increase and decrease in the percentage of CD27^-^ γδ T cells (**Figure 5E**) and in CD27^+^ γδ T cells (**Figure 5F**) was observed, respectively. These changes were observed independently of the degree of lympho-follicular hyperplasia. These analyses were done on CD3^+^ T cells but the same observations were made on the CD3^++^ T cells (**Supplementary Figure 5**). The variation of CD27 expression was specific to γδ T cells because we did not observe any change in the expression of CD27 on CD3^+^ γδ negative T cells (**Supplementary Figure 5**).

Butyrophilin (BTN) could shape γδ T cell differentiation profile and by analyzing by RT-PCR the expression of different subtypes of human BTN, we observed a selective increase in BTN1A expression in the thymus of MG patients (**Figures 5G–J**).

These results suggest that the thymic environment in MG patients affected γδ T cell differentiation and further investigations will be necessary to investigate the implication of these cells in the pathophysiology of MG.

### Decreased proportion of non-classical monocytes confirmed

**Figure 6:**
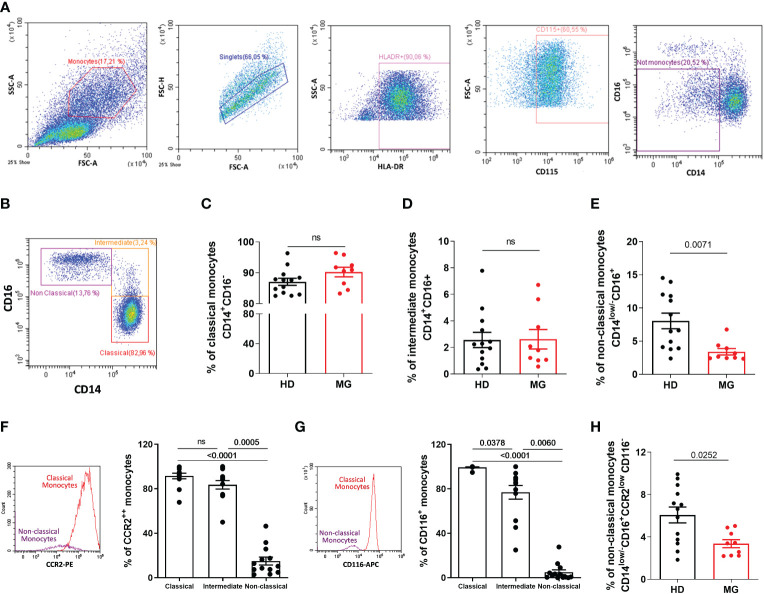
Analysis of monocytes by flow cytometry. Analysis by flow cytometry **(A–B)** of the percentages of classical **(C)**, CD14+CD16-), intermediate **(D)**, CD14+CD16+), and non-classical (**E)**, CD14low/-CD16+) monocytes in PBMCs from healthy individuals (HD) or in AChR MG patients (MG). Analysis of the percentage of CCR2 **(F)** and CD116 **(G)** expression by monocyte subpopulations in healthy donors. Percentage of newly defined non-classical monocytes **(H)**. p values were determined by the Mann-Whitney test **(C–E, H)** or the Kruskal-Wallis test with Dunn’s multiple comparisons test for multiple comparisons **(F, G)**.

CyTOF analyses on all PBMCs showed a decrease in all monocyte subpopulations in MG patients. We therefore analyzed by FACS, zooming in the SSC/FSC gate containing monocytes, the different monocyte subpopulations with a dedicated antibody panel. AChR MG patients were not thymectomized or on immunosuppressive treatments. To exclude contamination with NK cells (potentially CD16^+^ but HLA-DR^low/-^ and CD115^-^) and monocytic myeloid-derived suppressor cells (potentially CD14^+^ but HLA-DR^low/-^) (22), we gated on HLA-DR^+^ and CD115^+^ cells before analyzing monocytes based on CD14 and CD16 expression (**Figures 6A, B**). We observed a decrease in non-classical monocytes defined as CD14^low/-^CD16^+^ but no variation for classical and intermediate monocytes (**Figures 6C–E**). The classical definition of monocyte subpopulations based on CD14 and CD16 expression has strong limitations and monocytes are more heterogeneous as described by new multiparametric approaches (23) (24, 25). Since the definition of non-classical monocytes based on CD14^low/-^CD16^+^ may contain other cell populations such as conventional DC4 (cDC4) (26, 27), we further defined non-classical monocytes using two additional markers: CCR2 and CD116. These two markers are very weakly expressed in non-classical monocytes according to *protein atlas* (https://www.proteinatlas.org/) and as confirmed in our analyses (**Figures 6F, G**). We observed that non-classical monocytes defined as CD14^low/-^CD16^+^ CCR2^low^ CD116^-^ were also significantly decreased in MG patients (**Figure 6H**).

The same decrease in CD14^low/-^CD16^+^ non-classical monocytes was observed when the analyses were done on a larger gate containing all PBMCs, as for the CyTOF analysis. We could not explain why the decrease in classical and intermediate monocytes was not observed by classical cytometry. Monocytes are difficult to study because they suffer from cryopreservation and may be affected by the density gradient separation, but these difficulties should affect control and MG samples similarly both in CyTOF and FACS analyses (28).

### Decreased genes expression in monocytes affecting respiratory burst and inflammatory response

**Figure 7:**
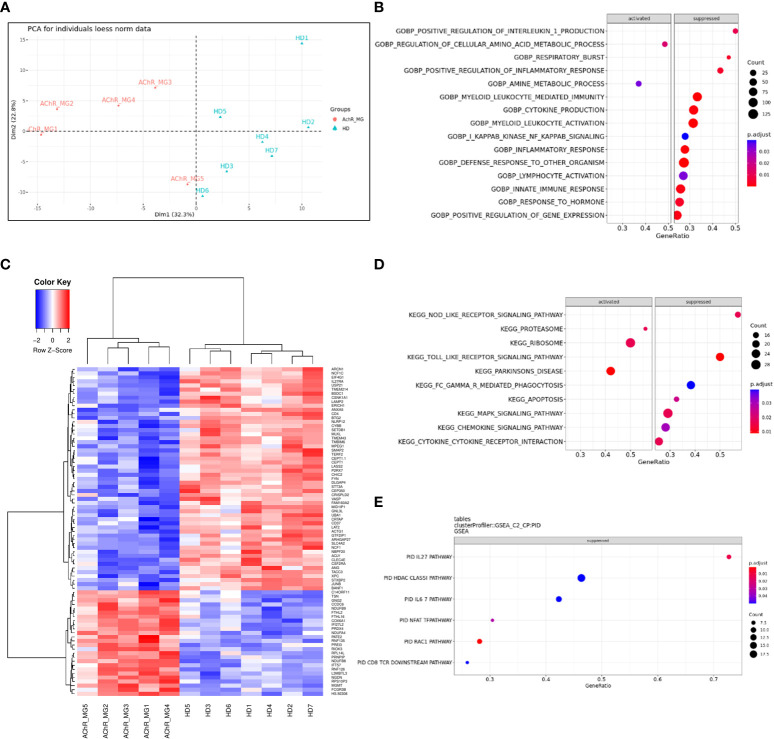
Dysregulated genes and pathways in monocytes from transcriptomic data. Principal component analysis of 200 most variable genes in the transcriptome (loess normalized expression). PC1, representing the majority of explained variances (32.3%), separates groups of anti-AChR positive and healthy subjects **(A)**. Unsupervised clustering of the top 100 most significant differentially expressed genes reveals similarity between groups of subjects (columns) and identifies top differentially up-regulated and down-regulated genes in circulating monocytes of patients (rows). Distance between features was measured by (1 - Kendall’s τ correlation coefficient) and clustering was performed using the Ward.D2 method. Similar results were obtained with alternative distance definitions and clustering procedures (not shown) **(B)**. Dot plot showing pathways that show a tendency for enrichment, determined from GSEA. Each dot plot demonstrates enriched pathways in subset gene sets from GO Biological Process ontology **(C)**, subset gene sets from PID pathway database **(D)**, and subset gene sets from KEGG pathway database **(E)**. The size of the dot represents gene count, and the color represents the adjusted p-value.

To better understand changes that might affect the monocytes of MG patients, we analyzed gene expression regulation using transcriptomic data from sorted CD14^+^ cells from very well-defined untreated AChR MG patients and healthy donors (**Supplementary Table S1C)**. PCA analysis showed that controls and MG patients were segregated by the first principal component (**Figure 7A**). We found 561 DEGs with an adjusted p-value below 0.05 (256 up- and 305 down-regulated genes). These genes were detailed in the supplementary data (**Supplementary Table S3**). We extracted the top-100 identified dysregulated genes excluding LOC genes and plotted a hierarchical clustering to reveal groups of genes with similar patterns of expression (**Figure 7B**). Among the 561 DEGs, 52 had a fold change above 2 with 17 up- and 35 down-regulated genes (**Supplementary Table S3**). There were 2 times more down-regulated genes suggesting that the global biological activity of monocytes could be decreased (**Figure 7A** and **Supplementary Table S3**).

The analyses of the 561 DEGs using ORA for biological processes showed a significantly decreased representation of genes associated with the “electron transport” and “respiratory burst” usually associated with the phagocyte capacity of phagocytes (data not shown). To analyze dysregulated functions or pathways with transcriptomic data, cut-offs for significance are somewhat arbitrary. To overcome this limitation, GSEA analyses use all genes to assess significance by weighting the scores (p-value and fold change). GSEA analyses for dysregulated biological processes demonstrated again a strong reduction of the respiratory burst function but also cytokine and chemokine production (**Figure 7C**) linked to innate and defense immune response, inflammatory response, cytokine production, and leukocyte/myeloid mediated immunity (**Supplementary Figure 6**). This is mainly associated with a decreased activity of the NOD- and Toll-like signaling KEGG pathways (**Figure 7D** and **Supplementary Figure 7A**) as previously observed (17), and of the IL-27, IFN-γ, and IL-6 PID (Pathway Interaction Database) pathways (**Figure 7E** and **Supplementary Figure 7A**). However, the proteasome, ribosome, and Parkinson’s disease KEGG pathways were strongly activated (**Figure 7D** and **Supplementary Figure 7A**).

Altogether, our results showed a global decrease activity of monocytes in MG patients. Decreased expression of genes involved in innate signaling pathways could alter the response to pathogen infection or monocyte-macrophage differentiation (29, 30).

## Discussion

In this study, by analyzing AChR MG peripheral blood cells with a multiparametric approach at the single-cell level, we brought to the forefront dysregulations concerning innate immune cells. The role of these cells has long been neglected in the study of autoimmune diseases considering that adaptive and innate immune cells were playing distinct roles in the functioning of the immune system.

### High-parameter single-cell phenotyping of MG patients

Using mass cytometry to study early-onset AChR MG patients, no particular changes were observed in the proportions for the main immune cell populations defined broadly as CD4 and CD8 T cells, γδ T cells, B cells, and NK cells, as observed for late-onset AChR MG patients by Ingelfinger et al. (14). However, we showed a decrease in monocytes in early-onset AChR MG patients that were not found in late-onset MG patients (14). By further analyzing the cell subpopulations, we observed a significant decrease in classical, intermediate, and non-classical monocytes but also an increase in ILC2 and CD27^-^ γδ T cells. At a less significant level, we detected a decrease in Breg cells as already studied in detail by Yilmaz et al. (4), and a slight increase in Treg cells that we did not explain clearly. This could be due to the inclusion in the analyses of CD4^+^CD25^int^ T cells which increase in MG patients (6) and not exclusively CD4^+^CD25^++^ T cells. A decreased proportion of CD8^+^CD161^+^ T cells was also detected. CD8 T cells expressing CD161 are subclassified as tissue-homing MAIT (Mucosal Associated Invariant T) cells, cytotoxic Tc17 cells, or memory T cells with stem cell-like phenotype (31). The CD8^+^CD161^+^ T cell population decreasing in our analysis did not correspond to Tc17 cells as they did not express CCR6 or CD127 (IL7-R) (**Figure 2B**). Our panel did not include MR1 (MHC-I related molecule) which would have allowed us to define this cell subset more precisely as MAIT. Nevertheless, a reduced frequency of CD8^+^CD161^+^ T cells has been observed in the blood of subjects with autoimmune diseases, such as in primary progressive multiple sclerosis (32) and systemic lupus erythematosus (33).

Unfortunately, we cannot compare our data with those of Ingelfinger et al. that investigated cell subpopulation changes by taking into account the cytokine expression profile. They observed a decreased proportion of two CD4^+^ T subpopulations: effector memory CD4^+^ T cells expressing GM-CSF, especially in newly diagnosed treatment-naïve MG patients with highly active disease, and CD103^+^CD4^+^ T cells described as tissue-resident T cells re-entering the circulation (14).

In this study, we further investigated cell subpopulations that were highly up- or down-regulated in our CyTOF analysis and that were not described so far to be dysregulated in MG patients: monocytes and γδ T cells. We did not further investigate the implication of ILC2. These cells belong to a heterogeneous group of immune cells that include ILC1, ILC2, and ILC3 expressing transcription factors and cytokines that reflect similarities to T helper cells, Th1, Th2, and Th17. ILC family also includes NK cells and lymphoid tissue inducer (LTi) cells. ILCs are involved in maintaining the integrity of the epithelium and homeostasis, tissue repair, and rapid response against pathogens. The role of these different ILC subpopulations in MG should be investigated in detail as their implication in autoimmunity is beginning to emerge (34).

### Dysregulation of γδ T cell subpopulations

γδ T cells are unconventional T cells at the crossroad of innate and adaptive immunity. They are mainly known to participate in host defense against pathogen infection, and cancer. However recent advances suggest their implication in autoimmunity (21). Here we observed by mass cytometry, but not by classical cytometry, an increase in the proportion of CD27^-^ γδ T cells in PBMCs of MG patients. This increase was further observed by classical cytometry in the thymus of MG patients regardless of the degree of thymic hyperplasia. It is known that immune cell alterations in early-onset MG are more pronounced in the thymus than in the periphery, as described for Treg cells and Th17 cells (6, 8).

γδ T cells differentiate in the thymus from double-negative thymocytes alongside the more well-known αβ T cells. In mice, γδ T cells with a specific γ chain develop by waves during fetal, neonatal, and adult development, and possess specific homing abilities to peripheral organs where they become long-lived tissue-resident γδ T cells. Mouse γδ T cells differentiate within the thymus into ready-to-act effector T cells with two distinct profiles: CD27^+^ γδ T cells producing IFN-γ and CD27^-^ γδ T cells producing IL-17 (21). In contrast in human, the commitment of γδ T cells is less well-known. Human fetal γδ thymocytes seem functionally programmed (35). However, this is not the case for post-natal γδ thymocytes that must complete their differentiation in the periphery (36). The differentiation of γδ T cells is dependent on the local environment and CD27^-^ γδ T cells can accumulate in inflamed tissues (37). It is more and more acknowledged that γδ T cells might play a role in autoimmunity, in particular CD27^-^ γδ T cells. Pathogenicity of CD27^-^ γδ T cells could be linked to their ability to recruit inflammatory myeloid populations, and to alter the balance between Th17 cells and regulatory αβ T cells (37).

Peripheral activation of γδ T cells can rely on TCR activation and cytokines, mainly IL-1β, IL-23 but also IL-6, TGF-β (37, 38). All these cytokines are known to be overexpressed in the thymus of MG patients (8, 38). IL-7 signaling could also favor the differentiation of CD27^-^ γδ T cells (39) and this cytokine is increased in the thymus of AChR MG patients (data not shown). γδ T cells respond to both self- and non-self-phosphoantigens, and butyrophilins (BTNs) play a key role in mediating phosphoantigen sensing by γδ T cells (40). BTNs are members of the immunoglobulin superfamily. They have sequence similarities with the B7 family of costimulatory receptors (including CD80, CD86, PDL1 (Programmed Cell Death Ligand-1), ICOS (Inducible T Cell Costimulator) ligand, and B7 homolog) (41). We observed an increased expression of BTN1A1 in the thymus of MG patients. If BTN1A1, is required for milk lipid secretion in lactation by mammary epithelial cells, it is also expressed in the spleen and thymus, in particular by thymic epithelial cells (42). BTN1A expression is also increased during inflammation in ulcerative colitis (43). Consequently, the overexpression of BTN1A1 could affect γδ T cell differentiation and/or activation but many things remain unknown about BTNs.

While αβ T cells are extremely well known, knowledge about γδ T cells is only beginning to emerge. These cells have long been considered to play a specific role in the innate immune response, especially in epithelial tissues with a barrier role. There is still a long way to go to define their role in autoimmunity and AChR MG in particular in the inflammatory thymus.

### Dysregulation of monocyte subpopulations

Monocytes are key innate immune cells that play important role in health and disease. In past years, mass cytometry and single-cell RNA sequencing analyses have been powerful tools to better defined monocytes, underlying, in particular, the complexity of monocyte subpopulations (23, 25, 44–47).

Here, by CyTOF analysis on PBMCs, we observed a decrease in all monocyte subpopulations. In a single-cell RNA sequencing analysis of PBMCs, an analysis of cluster abundance revealed also a decreased proportion of CD14^+^ or CD16^+^ monocytes in MG patients (48). Analyzing the transcriptome changes in CD14^+^ sorted cells, we observed a global down-regulated profile in gene expression for AChR MG patients which could correspond to an exhausted profile of monocytes. GSEA pathway analyses showed a decreased expression of genes involved in the respiratory burst function suggesting an altered phagocyte capacity of monocytes in MG patients. A reduced phagocytic capacity of monocytes was found in patients with rheumatoid arthritis (49) and Sjögren’s syndrome patients (50). These altered phagocyte capacities could be also found in monocyte-derived macrophages as observed in various autoimmune diseases (51).

GSEA analyses showed a strong reduction of cytokine and chemokine production linked to innate/defense immune response and inflammatory response was observed in monocytes from MG patients. In particular, Toll- and NOD-like receptor signaling pathways as previously observed by Mamrut et al. (17). The IL-27 pathway was also strongly down-regulated. IL-27 is known to modulate autoimmune inflammation and may affect the Treg/Th17 cell balance by acting as a negative regulator of Th17 commitment (52).

We do not explain clearly the differences between CyTOF and FACS analyses for monocyte subpopulations. The global decrease of MG monocytes observed with the CyTOF analysis could be associated with the gating strategy focusing on CD45^+^ cells. Indeed, among the DEG in monocytes, a decrease in CD45 (Protein tyrosine phosphatase, receptor type, C (PTPRC)) gene was observed in MG patients (**Table 1**). Nevertheless, a decrease in monocytes in AChR MG is also suggested by Jin et al. (48) and also Weiss et al. who observed a slight but significant decrease in CD14 cells (53). Human monocyte subpopulations are usually defined according to the levels of CD14 and CD16 expression: the classical CD14^+^CD16^−^, intermediate CD14^+^CD16^+^, and non-classical CD14^low^CD16^+^ or CD14^−^CD16^+^ depending on the gating strategy in each study. The gate containing non-classical monocytes is often contaminated by other cells. Here, using classical flow cytometry, excluding NK, myeloid cells, and also cDC4, we observed a clear decrease of non-classical monocytes defined as CD14^low/-^CD16^+^ but also as CD14^low/-^CD16^+^/CCR2^low^/CD116^-^. Non-classical monocytes are described as patrolling blood cells that selectively detect damaged and virally infected cells and produce proinflammatory cytokines (22). The altered activity of the NOD- and Toll-like signaling pathways detected in the transcriptomic analyses could thus reflect the decrease in non-classical monocytes in MG patients.

Increased or decreased levels of peripheral non-classical monocytes have been observed in autoimmune diseases (54, 55). Monocytes are recruited from the blood when tissue homeostasis is disturbed. They undergo a specific differentiation into macrophages depending on the local tissue environment. In lupus nephritis, patients have higher CD16^+^ monocyte counts in glomeruli and decreased frequency in peripheral blood suggesting the recruitment of non-classical monocytes to renal tissues (56). A similar hypothesis could apply to MG patients. Non-classical monocytes could be recruited to inflammatory MG thymuses. However, our team recently observed a decrease in the number of thymic macrophages in AChR-MG patients (57). Non-classical monocytes derived from the linear differentiation of classical monocytes into intermediate monocytes associated with the loss and gain expression of CD14 and CD16, respectively. Consequently, the decrease of non-classical monocytes could reflect an altered differentiation process of monocytes. In a comparative study of twins discordant for multiple sclerosis with high-dimensional single-cell technologies, a decrease in non-classical monocytes is observed in twins with multiple sclerosis. A shift in the myeloid compartment is suggested to drive away non-classical monocytes toward the inflammatory classical monocytes (18). In this study, it is also demonstrated that non-classical monocytes are more sensitive to environment-induced changes as compared to classical and intermediate monocytes which are mainly under genetic influence (18). This suggests that the decrease in non-classical monocytes in MG could be attributable to a specific disease environment.

## Conclusion

Regarding MG and other B-cell mediated autoimmune diseases, immune dysregulation is well known for adaptive immune cells (3–11). Our results show for the first time dysregulations affecting innate immune cells in AChR MG patients. To date, it is not clear whether these disturbances are the cause or the consequence of the disease. However, our study suggests that new research axes must be set up to define the role of these cells in Myasthenia Gravis and more globally in autoimmune diseases.

## Data availability statement

Mass cytometry data is available in FlowRepository (https://flowrepository.org/) under Repository ID: FR-FCM-Z6ZQ. CD14 monocyte gene expression data were already available on GEO (GSE85649). Other data are available from the corresponding author.

## Ethics statement

The studies involving human participants were reviewed and approved by Comité consultatif de protection des personnes), Ile-de-France VII (Kremlin-Bicêtre, France). Written informed consent to participate in this study was provided by the participants’ legal guardian/next of kin.

## Author contributions

JV, EH, O-MF, FT performed and analyzed the experiments, FT collected samples and provided patient information. NP performed bioinformatics analyses on transcriptomics data. SD and AB provided blood samples. AC and CB help performing mass cytometry analyses. EF and JG provided thymic biopsies. SB-A and JV revised the manuscript. JV, SB-A and RP designed the study, analyzed the experiments and wrote the manuscript. All authors contributed to the article and approved the submitted version.
