# Supplementary material for: Single-cell mass cytometry on peripheral cells in Myasthenia Gravis identifies dysregulation of innate immune cells
Source: Front Immunol. 2023 Jan 30;14:1083218. doi: 10.3389/fimmu.2023.1083218 (PMC9922723; doi:10.3389/fimmu.2023.1083218)
Supplement: Supplementary file 1 [file DataSheet_1.pdf]

Supplementary data

Single-cell mass cytometry on peripheral cells in Myasthenia Gravis identifies dysregulation of innate immune cells

Verdier et al.

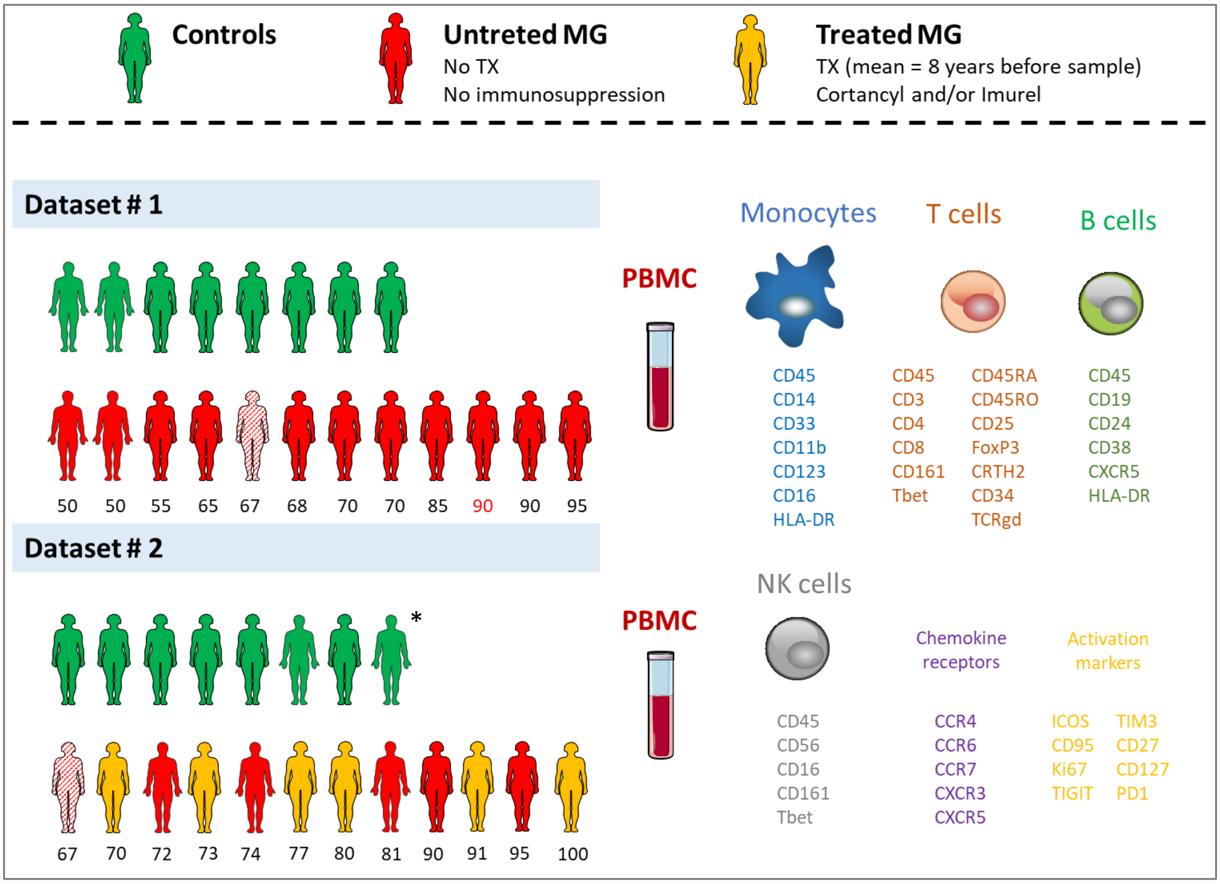

* Excluded from the analyses due to an abnormally low percentage of immune cells

Supplementary Fig. 1: Graphical summary of AChR MG patients and markers used in the CyTOF analysis

Graphical summary of the two datasets and of the different markers used in this study. Healthy sex- and age-matched donors are represented in green, untreated patients in red, and treated patients in orange. The patient with red stripes corresponds to the common donor used in the two datasets. The numbers below patients correspond to clinical scores. MG severity was evaluated with the quantitative Myasthenia Muscle Score (MMS) based on a scale of 0 to 100: lower scores represent greater symptom severity [40]. Patients were thymectomized (TX).

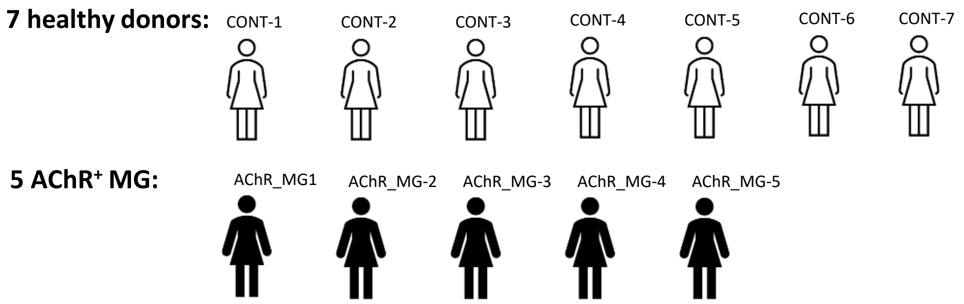

**Healthy donors**		**AChR$^+$-MG**	
Name in this analysis	GEO (GSE85649) link	Name in this analysis	GEO (GSE85649) link
CONT-1	GSM2267284	AChR_MG-1	GSM2267269
CONT-2	GSM2267288	AChR_MG-2	GSM2267270
CONT-3	GSM2280174	AChR_MG-3	GSM2267276
CONT-4	GSM2280166	AChR_MG-4	GSM2267275
CONT-5	GSM2267267	AChR_MG-5	GSM2267277
CONT-6	GSM2267266		
CONT-7	GSM2267285		

Supplementary Fig. 2: Graphical summary of AChR MG patients involved in the transcriptomic analysis

AChR MG patients from the Gene Expression Omnibus (GEO) study (GSE85649) were selected according to strict criteria: patients with MG symptoms, no thymoma, AChR antibodies, no immunosuppressive treatment, and no other autoimmune disease (Table S1C). For healthy donors, 2 donors were removed corresponding to duplicate samples

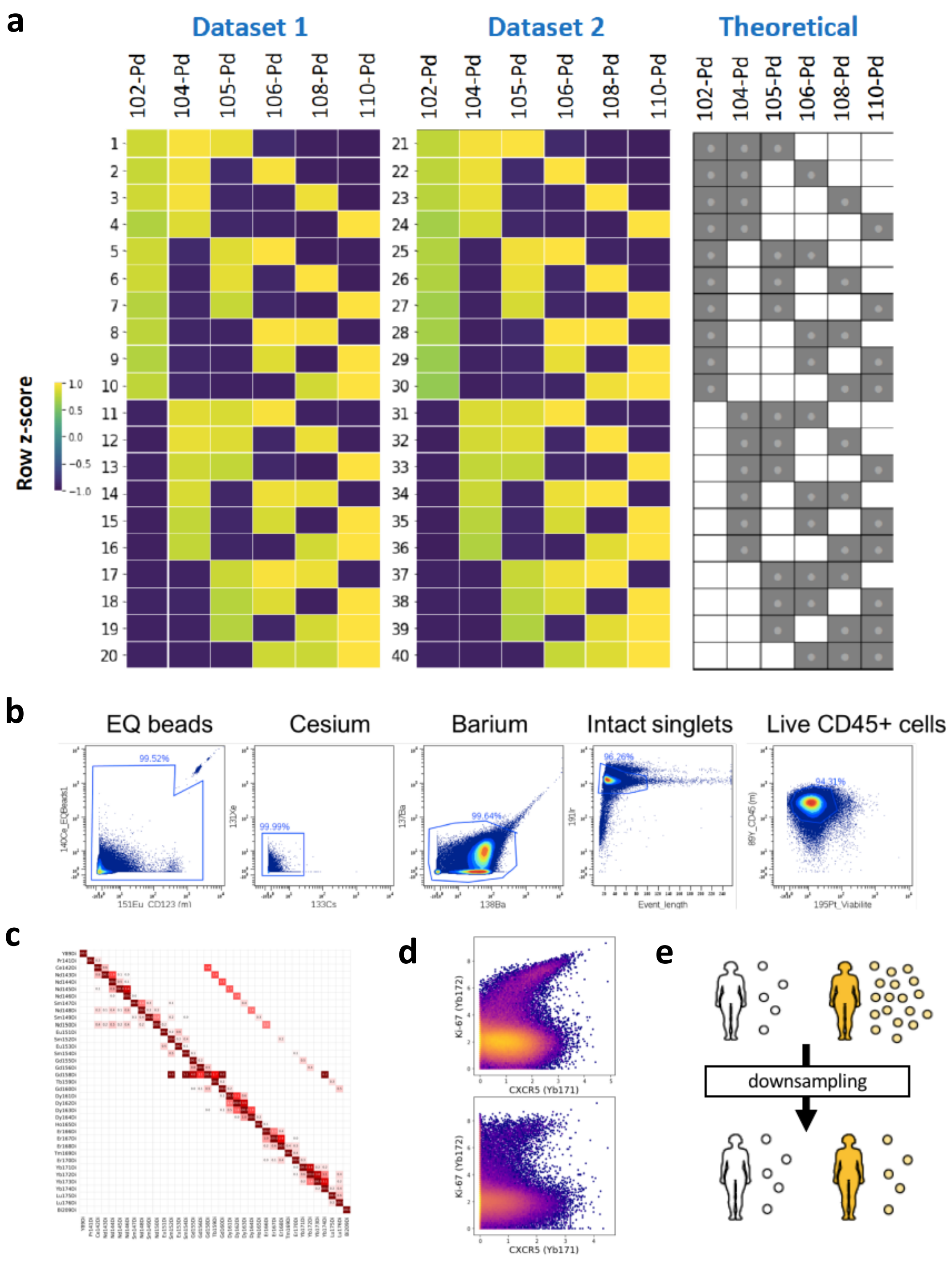

Supplementary Fig. 3 – Cell pre-processing for CyTOF analysis
(a) Heatmaps showing relative expression of Pd mean metal intensities (MMI) in ungated populations for the two datasets and what is expected to obtain theoretically. (b) Representative gating strategy to exclude EQ beads and contaminants (cesium and barium) and to select intact single-cells and live CD45+ cells. (c) Spillover matrix used to compute NNLS compensations. (d) Dot plots comparing the absence (top) and the presence (bottom) of NNLS compensations. (e) Equal down-sampling of events.

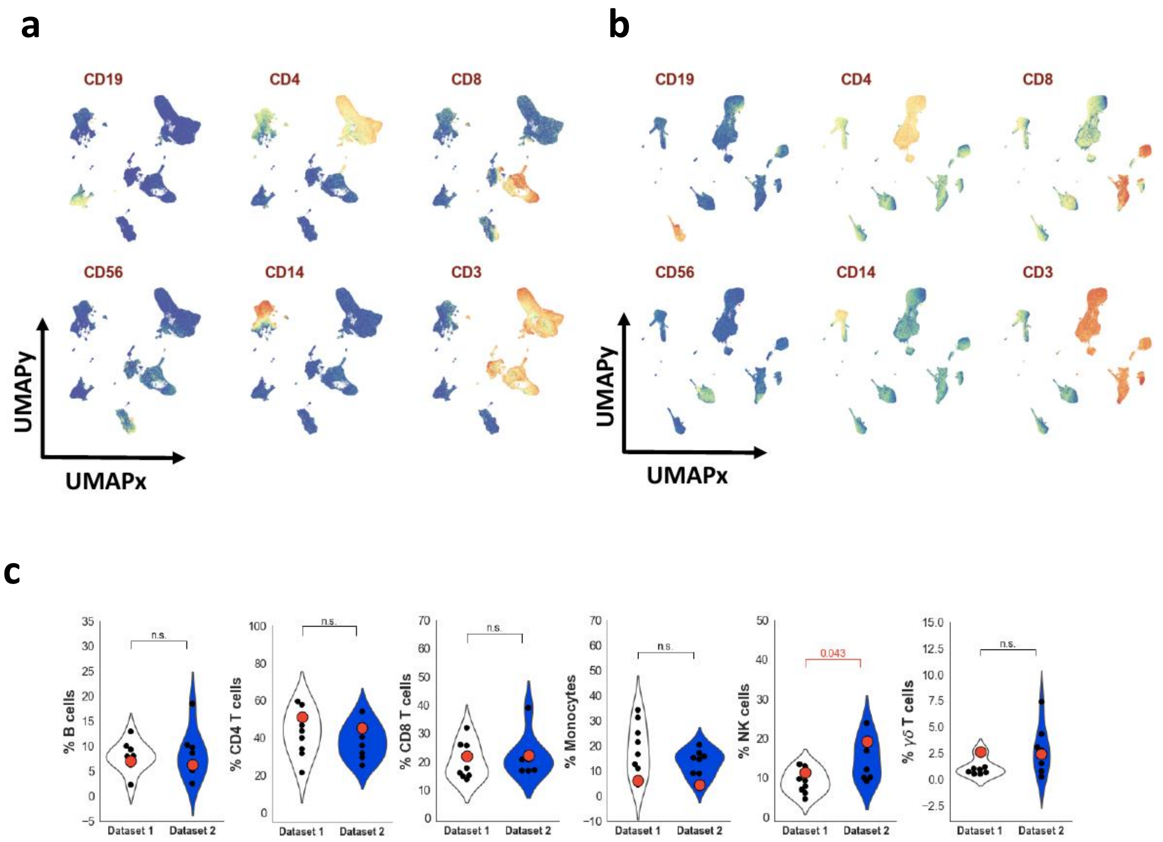

Supplementary Fig. 4 – Datasets harmonization

(a) Distribution of cell population percentages and differences between controls from dataset 1 and dataset 2 for the main populations. The red dot corresponds to the common donor (a patient) used as an indicator of cell population consistency between the two datasets. (b) Polar charts indicating the relative mean metal intensities (MMI) of canonical markers for the two overlaid datasets (dataset 1 in blue, dataset 2 in red) for the indicated cell populations. (c) Distribution of cell population percentages and differences between controls from dataset 1 and dataset 2 for the subpopulations. The red dot corresponds to the common donor used as an indicator of cell population consistency between the two datasets

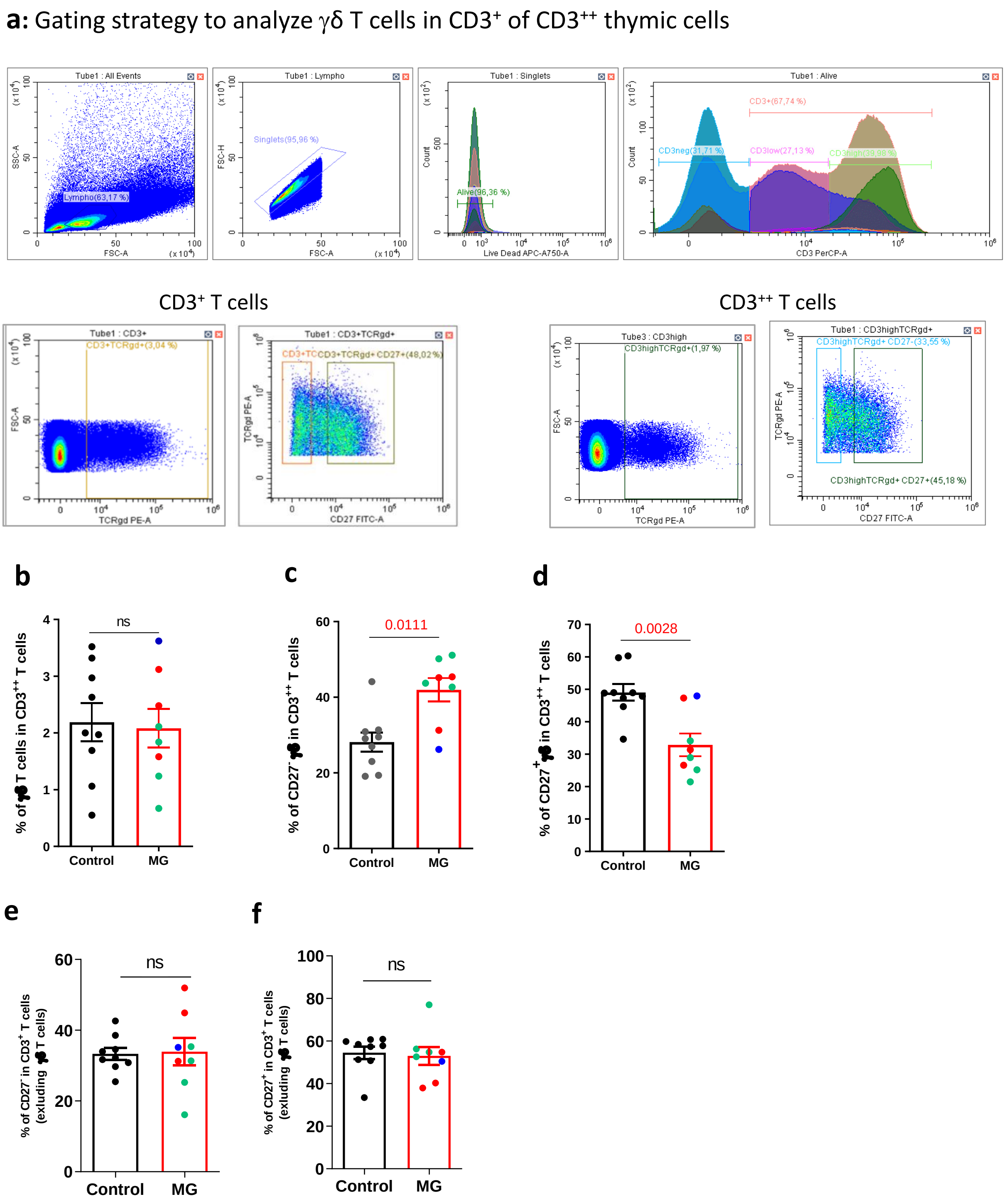

Supplementary Fig. 5 – Analysis of thymic γδ T cells in CD3++ T cells in controls and MG patients

Gating strategy for the analyses of the percentage of γδ T cells in CD3+ or CD3++ thymic cells (**a**). Percentage of γδ T cells (**b**), of CD27- (**c**) and CD27+ (**d**) in CD3++ T cells+ γδ T cells in thymic cells, from healthy individuals (HD) or in AChR MG patients (MG). Percentage of CD27- (**e**) and CD27+ (**f**) in CD3++ T cells excluding γδ T cells in healthy individuals (HD) or AChR MG patients (MG). The degree of thymic follicular hyperplasia was indicated: numerous (red dots), few (green dots), and none (bleu dot) germinal centers. Statistical test: two-tailed Mann-Whitney.

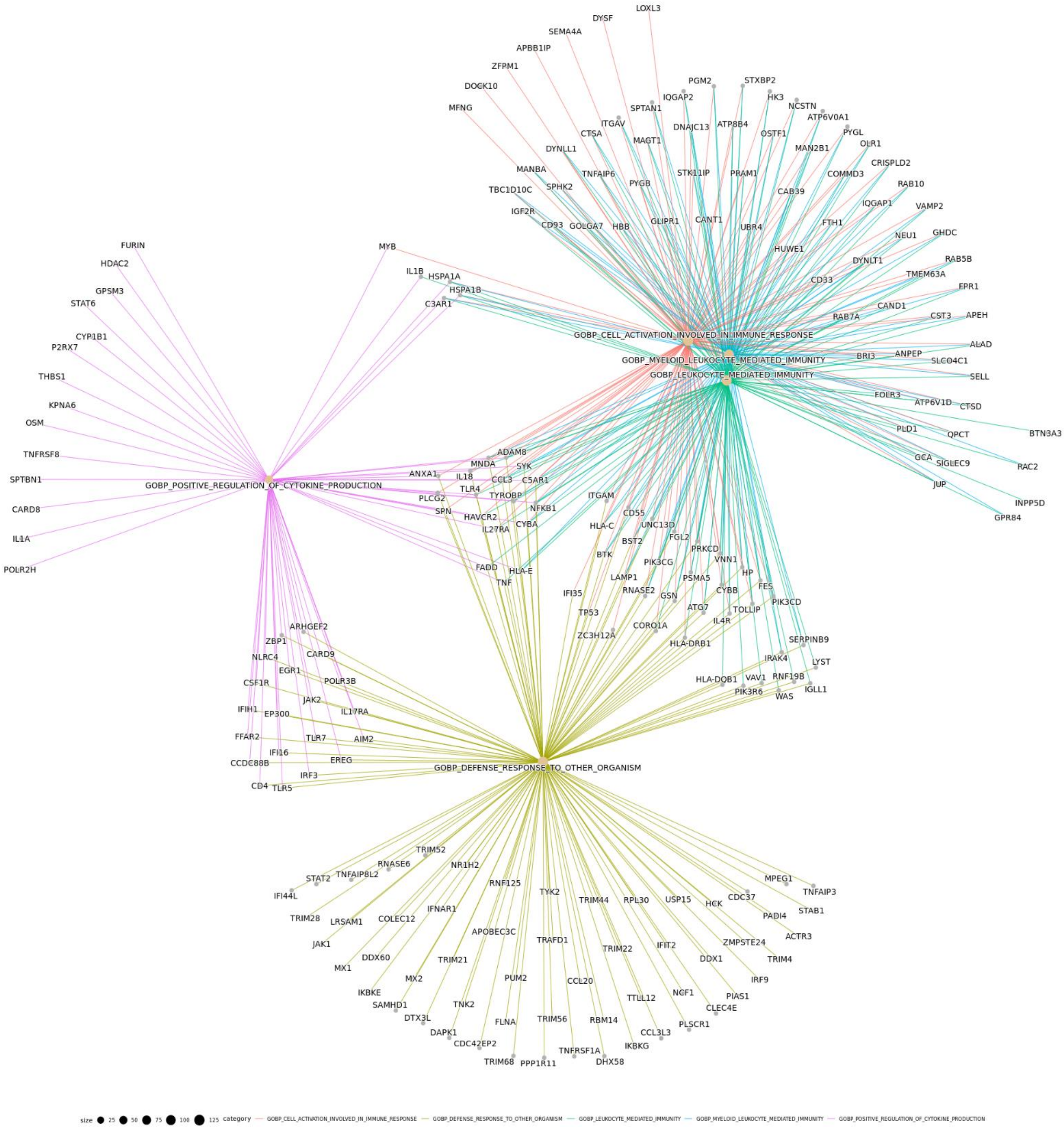

Supplementary Fig. 6 – GSEA analyses of dysregulated GO Biological processes in peripheral CD14^{+} cells from MG patients

Cnetplot depicting the genes behind each pathway and the link between functional profiles and gene clusters. The number of enriched pathways to show was limited to the 5 most significant ones

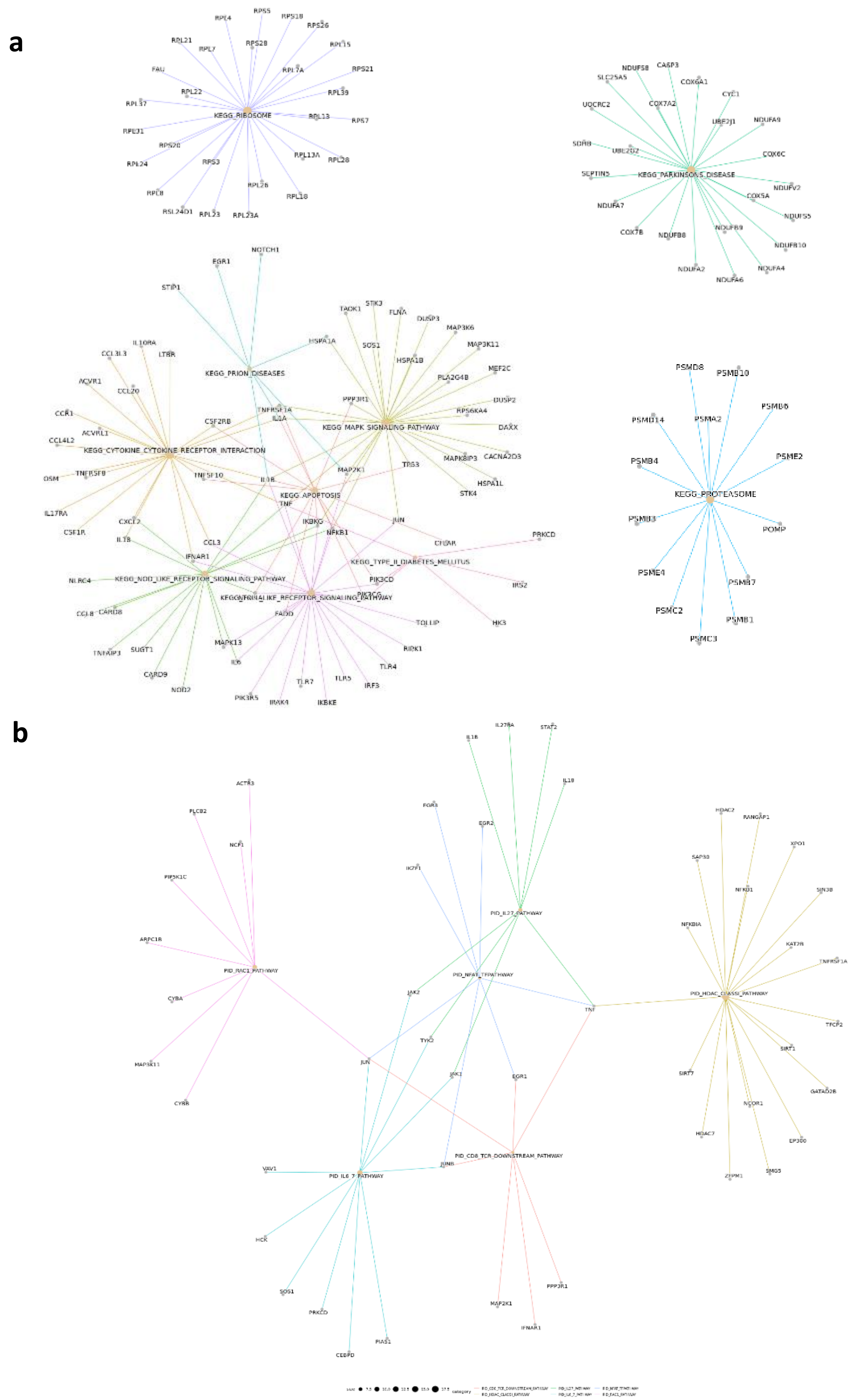

Supplementary Fig. 7 – GSEA analyses of dysregulated KEGG and PID pathways processes in peripheral CD14^{+} cells from MG patients

Cnetplot depicting the genes behind each pathway and the link between functional profiles and gene clusters for KEGG (**a**) and PID (**b**) pathways. The number of enriched pathways to show was limited to the 10 most significant ones
