# Supplementary material for: Single-cell mass cytometry on peripheral cells in Myasthenia Gravis identifies dysregulation of innate immune cells
Source: Front Immunol. 2023 Jan 30;14:1083218. doi: 10.3389/fimmu.2023.1083218 (PMC9922723; doi:10.3389/fimmu.2023.1083218)
Supplement: Supplementary file 2 [file DataSheet_2.pdf]

All supplementary tables

Supplemental table 1A : List of MG patients and controls included in CyTOF analyses.
For figures 1-4 and supplementals figures 1, 3 and 4

MG Patient	Gender	Age (years)	Anti-AChR titer (nmol/L)	Anti-cholinesterase drugs	Corticoids - Immunosupresors	Thymectomy	MMS Score*/100	MGFA Score	Dataset CyTOF
1	F	33	66.7	Yes	No	No	70	IIb	1
2	F	17	57.5	Yes	No	No	65	IIa	1
3	M	47	39.0	Yes	No	No	50	IIIb	1
4	F	33	100.0	Yes	No	No	90	IIIb	1
5	F	41	23.0	Yes	No	No	85	Unknown	1
6	F	19	56.2	Yes	No	No	55	IIIb	1
7	F	25	100.0	Yes	No	No	67	IIb	1 & 2
8	F	23	105.4	Yes	No	No	70	IIIb	1
9	F	32	100.0	Yes	No	No	68	IIb	1
10	F	28	36.4	Yes	No	No	95	Ia	1
11	M	17	2.4	Yes	No	No	50	IVb	1
12	M	17	0.4	Yes	No	No	85	Unknown	1
13	F	30	100.0	Yes	No	No	90	Unknown	2
14	F	25	7.0	Yes	No	No	95	Unknown	2
15	F	44	75.2	Yes	Yes	Yes	70	Unknown	2
16	F	53	10.9	Yes	Yes	Yes	100	Unknown	2
17	M	47	9.9	No	No	No	72	Unknown	2
18	M	21	3.3	Yes	No	No	74	Unknown	2
19	M	26	18.7	Yes	No	No	81	Unknown	2
20	F	37	100.0	Yes	Yes	Yes	77	Unknown	2
21	F	34	48.0	Yes	Yes	Yes	80	Unknown	2
22	F	38	100.0	Yes	Yes	Yes	73	Unknown	2
23	M	32	100.0	Yes	Yes	Yes	91	Unknown	2

* MG severity was evaluated with the quantitative Myasthenia Muscle Score (MMS) based on a scale of 0 to 100:
lower scores represent greater symptom severity (Sharshar et al., J. Neurol. 2000 J Neurol (DOI: 10.1007/s004150050585))

Adult Controls	Gender	Age (years)	Dataset CyTOF
1	F	41	1
2	F	28	1
3	F	32	1
4	M	52	1
5	F	35	1
6	F	40	1
7	M	27	1
8	F	32	1
9	F	41	2
10	F	19	2
11	F	32	2
12	F	52	2
13	F	47	2
14	M	44	2
15	F	22	2
16 **	M	25	2

** Excluded from the analyses due to an abnormally low percentage of immune cells

Supplemental table 1B : List of MG patients and controls included in flow cytometry analyses
For figures 5 A-C and 6 A-H

MG Patient	Gender	Age (years)	Anti-AChR titer (nmol/L)	Anti-cholinesterase drugs	Corticoids - Immunosupresors	Thymectomy	MMS Score*/100	MGFA Score	Comment
1	M	47	0.96	Yes	No	No	70	IIIa	
2	M	25	35.3	Yes	No	No	75	IIb	**
3	F	22	100	Yes	No	No	72	Unknown	
4	F	24	100	Yes	No	No	67	Unknown	
5	M	33	7.9	Yes	No	No	Unknown	Unknown	**
6	F	30	100	No	No	No	88	IIa	
7	F	28	1.92	Yes	No	No	Unknown	I	
8	F	49	14.5	No	No	No	100	I	
9	F	35	10.9	Yes	No	No	100	I	
10	M	39	0.65	No	No	No	90	I	
11	M	17	0.76	Yes	No	No	Unknown	I	

* MG severity was evaluated with the quantitative Myasthenia Muscle Score (MMS) based on a scale of 0 to 100:
lower scores represent greater symptom severity (Sharshar et al., J. Neurol. 2000 J Neurol (DOI: 10.1007/s004150050585))
** Excluded from monocyte analysis as the % of classical monocyte was below 25%

Healthy Donors	Gender	Age (years)
1	M	38
2	F	48
3	F	30
4	M	22
5	M	49
6	F	27
7	F	35
8	F	23
9	F	44
10	F	28
11	M	33
12	M	48
13	F	40

Supplemental table 1C : List of MG patients and controls included in transcriptomic analyses
For figures 7 A-E and supplementals figures 2, 6 and 7

MG Patient	GEO Accession number	Gender	Age at onset (Years)	Age at sampling (years)	Anti-AChR titer (nmol/L)	Anti-cholinesterase drugs	Corticoids - Immunosupresors	Thymectomy	MMS Score*/100	MGFA Score
AChR_MG1	GSM2267269	F	20	54	18.8	Yes	No	Yes at 21 y/o	Unknown	Unknown
AChR_MG2	GSM2267270	F	15	54	>100	No	No	Yes at 18 y/o	Unknown	Unknown
AChR_MG3	GSM2267276	F	29	59	Yes but 0 at sampling	Yes	No	Yes at 34 y/o	Unknown	IIa
AChR_MG4	GSM2267275	F	29	59	3.6	Yes	No	Yes at 34 y/o	Unknown	IIa
AChR_MG5	GSM2267277	F	31	35	11.1	Yes	No	Yes at 32 y/o	Unknown	IIb

* MG severity was evaluated with the quantitative Myasthenia Muscle Score (MMS) based on a scale of 0 to 100:
lower scores represent greater symptom severity (Sharshar et al., J. Neurol. 2000 J Neurol (DOI: 10.1007/s004150050585))

Healthy Donors	GEO Accession number	Gender	Age (years)
HD1	GSM2267284	F	23
HD2	GSM2267288	F	32
HD3	GSM2280174	F	33
HD4	GSM2280166	F	36
HD5	GSM2267267	F	41
HD6	GSM2267266	F	52
HD7	GSM2267285	F	49

Supplemental table 1D : List of MG patients and controls in thymic cell analyses by flow cytometry
For figures 5 D-E

MG Patient	Gender	Age at thymectomy (years)	Anti-AChR titer (nmol/L)	Anti-cholinesterase drugs	Corticoids - Immunosupresors	Germinal centers	MMS Score*/100	MGFA Score
1	F	25	6.99	yes	no	Few	95	II/III
2	M	13	100	yes	no	Numerous	40	IIb
3	F	27	1.14	yes	no	Few	75	IIa
4	F	12	9.63	yes	no	Numerous	25	IVb
5	F	34	101	yes	no	None	76	IIb
6	F	21	39.7	yes	no	Numerous	60	IIIb
7	F	19	26.2	Yes	no	Numerous	Unknown	IIb
8	M	21	87.6	yes	no	Few	78	IIa
9	F	28	100	yes	no	Numerous	75	IIb
10	M	28	11.8	yes	no	Few	29	Unknown

* MG severity was evaluated with the quantitative Myasthenia Muscle Score (MMS) based on a scale of 0 to 100:
lower scores represent greater symptom severity (Sharshar et al., J. Neurol. 2000 J Neurol (DOI: 10.1007/s004150050585))

Healthy Donors	Gender	Age (years)
1	F	22
2	M	22
3	F	15
4	F	17
5	F	29
6	M	13
7	F	26
8	F	33
9	M	31
10	F	15

Supplemental table 1E : List of MG patients and controls for RT-PCR analyses

For figures 5 F-H

MG Patient	Gender	Age (Years)	Anti-AChR titer (nmol/L)	Anti-cholinesterase drugs	Corticoids - Immunosupresors	Thymectomy	MMS Score*/100	MGFA
1	F	23	>100	yes	No	yes	75	IIa
2	F	29	83.7	yes	No	yes	90	Ia
3	F	35	2.6	yes	No	yes	40	IVa
4	F	33	7.8	yes	No	yes	nd	IIb
5	F	31	>100	yes	No	yes	30	IVa
6	F	31	3181	yes	No	yes	90	I
7	F	25	3.2	yes	No	yes	90	IIa
8	F	28	60.4	yes	No	yes	70	IIa
9	F	28	>9.7	yes	No	yes	nd	IIIa
10	F	22	264	yes	No	yes	nd	IIIa

Healthy Donors	Gender	Age (Months/Years)
1	F	3 M
2	F	7 M
3	F	12 M
4	F	3 M
5	F	12 M
6	F	7 M
7	F	15 Y
8	F	18 Y
9	F	33 Y
10	F	37 Y
11	F	23 Y
12	F	35 Y

Supplemental table 2A : List of antibodies for mass cytomety

**Cat. No**	**Company**	**Antigen**	**Full Name**	**Clone**	**Custom labeling**	**Metal**
**3144001B**	Fluidigm	CD11b (Mac-1)	Anti-Human CD11b/Mac-1 (ICRF44)-144Nd—100 Tests	ICRF44		144Nd
**3145001B**	Fluidigm	CD4	Anti-Human CD4 (RPA-T4)-145Nd—100 Tests	RPAT4		145Nd
**3146001B**	Fluidigm	CD8a	Anti-Human CD8 (RPA-T8)-146Nd—100 Tests	RPAT8		146Nd
**555513**	BD	CD56 (NCAM) *NCAM16.2*	Purified NA/LE Mouse Anti-Human CD56	B159	20170606	147Sm
**3148010B**	Fluidigm	CD14	Anti-Human CD14 (RMO52)-148Nd—100 Tests	RMO52		148Nd
**3149013B**	Fluidigm	CD34	Anti-Human CD34 (581)-149Sm—100 Tests	581		149Sm
**BLE303127**	Ozyme	CD31 *WM59*	Purified anti-human CD31 (MaxPar Ready)	WM59	20170608	150Nd
**3151001B**	Fluidigm	CD123 (IL-3R)	Anti-Human CD123/IL-3R (6H6)-151Eu—100 Tests	6H6		151Eu
**3152008B**	Fluidigm	TCRgd	Anti-Human TCRgd (11F2)-152Sm—100 Tests	11F2		152Sm
**3153008B**	Fluidigm	TIM-3	Anti-Human TIM-3 (F38-2E2)-153Eu—100 Tests	F382E2		153Eu
**3154016B**	Fluidigm	TIGIT	Anti-Human TIGIT (MBSA43)-154Sm—100 Tests	MBSA43		154Sm
**3155011B**	Fluidigm	CD45RA	Anti-Human CD45RA (HI100)-155Gd—100 Tests	HI100		155Gd
**3156004B**	Fluidigm	CD183 (CXCR3)	Anti-Human CD183/CXCR3 (G025H7)-156Gd—100 Tests	G025H7		156Gd
**3158010B**	Fluidigm	CD27	Anti-Human CD27 (L128)-158Gd—100 Tests	L128		158Gd
**3159003A**	Fluidigm	CD197 (CCR7)	Anti-Human CD197/CCR7 (G043H7)-159Tb—50 Tests	G043H7		159Tb
**3160010B**	Fluidigm	Tbet	Anti-Human/Mouse Tbet (4B10)-160Gd—100 Tests	4B10		160Gd
**BLE303419**	Ozyme	CD33 *WM53*	Purified anti-human CD33 (MaxPar Ready)	WM53	20170608	161Dy
**3162024A**	Fluidigm	FoxP3	Anti-Human FoxP3 (259D/C7)-162Dy—50 Tests	259D/C7		162Dy
**3163003B**	Fluidigm	CD294 (CRTH2)	Anti-Human CD294/CRTH2 (BM16)-163Dy—100 Tests	BM16		163Dy
**3164009B**	Fluidigm	CD161	Anti-Human CD161 (HP-3G10)-164Dy—100 Tests	HP3G10		164Dy
**3165011B**	Fluidigm	CD45RO	Anti-Human CD45RO (UCHL1)-165Ho—100 Tests	UCHL1		165Ho
**3166007B**	Fluidigm	CD24	Anti-Human CD24 (ML5)-166Er—100 Tests	ML5		166Er
**3167001B**	Fluidigm	CD38	Anti-Human CD38 (HIT2)-167Er—100 Tests	HIT2		167Er
**3168024B**	Fluidigm	CD278/ICOS	Anti-CD278/ICOS (C398.4A)-168Er—100 Tests	C398.4A		168Er
**3169003B**	Fluidigm	CD25 (IL-2R)	Anti-Human CD25 (2A3)-169Tm—100 Tests	2A3		169Tm
**3170001B**	Fluidigm	CD3	Anti-Human CD3 (UCHT1)-170Er—100 Tests	UCHT1		170Er
**3171006B**	Fluidigm	CD185 (CXCR5)	Anti-Human CD185/CXCR5 (51505)-171Yb—100 Tests	51505		171Yb
**3172024B**	Fluidigm	Ki-67	Anti-Ki-67 (B56)-172Yb—100 Tests	B56		172Yb
**555670**	BD	CD95/Fas *DX2*	Purified NA/LE Mouse Anti-Human CD95	DX2	20170606	173Yb
**3174020B**	Fluidigm	CD279 (PD-1)	Anti-Human CD279/PD-1 (EH12.2H7)-174Yb—100 Tests	EH12.2H7		174Yb
**3175021A**	Fluidigm	CD194 (CCR4)	Anti-Human CD194/CCR4 (205410)-175Lu—50 Tests	205410		175Lu
**3176004B**	Fluidigm	CD127 (IL-7Ra)	Anti-Human CD127/IL-7Ra (A019D5)-176Yb—100 Tests	A019D5		176Yb
**3209002B**	Fluidigm	CD16	Anti-Human CD16 (3G8)-209Bi—100 Tests	3G8		209Bi
**3142001B**	Fluidigm	CD19	Anti-Human CD19 (HIB19)-142Nd—100 Tests	HIB19		142Nd
**3143013B**	Fluidigm	HLA-DR	Anti-Human HLA-DR (L243)-143Nd—100 Tests	L243		143Nd
**3141003A**	Fluidigm	CD196 (CCR6)	Anti-Human CD196/CCR6 (G034E3)-141Pr—50 Tests	G034E3		141Pr
**3089003B**	Fluidigm	CD45	Anti-Human CD45 (HI30)-Y89—100 Tests	HI30		Y9Y

Supplemental table 2B : List of antibodies for flow cytometry

Target	Fluorochrome	Company	Clone	Reference
CD3	PerCP-Cy5.5	BioLegend	OKT3	317305
TCR gamma/delta	PE	BD Biosciences	B1	555717
CD27	FITC	BioLegend	M-T271	356404
CD14	PE-Cy7	BD Pharmingen	M5E2	557742
CD16	FITC	BioLegend	3G8	302005
HLA-DR	BV510	BioLegend	L243	307645
CD115	PE/Dazzle™ 594	BioLegend	9-4D2-1E4	347319
CD116	APC	BioLegend	4H1	305913
CCR2	PE	BioLegend	K036C2	357205
Live Dead	APC-Cy7	Invitrogen	-	L10120

Supplemental table 2C : List of primers

Gene	Human primers	
**BTN1A1**	GTTAGCTTCAACTGCCTCCG	(f)
	TCAGGGGCAAATACACAGGT	(r)
**BTN2A1**	TGCTCGGCCAGAAGAAAGAA	(f)
	CCACAATGATAGGCAGGGC	(r)
**BTN2A2**	TCTCCAGCCTACAGATGAGC	(f)
	TGCCTCATTTTATTAGTCAGGGT	(r)
**BTN3A1**	GGAGGGTGTATCCTGTACCATCA	(f)
	AAGAAGCAGCAGCAAGACAGG	(r)
**28S**	CGGGTAAACGGCGGGAGTAA	(f)
	GGTAGGGACAGTGGGAATCT	(r)

Table S3: List of DEG in CD14⁺ monocytes in MG patients versus healthy donors

Row.names	ACCESSION	ILMN_GENE	logFC	FC	adj.P.Val	CHROMOSOME	DEFINITION	cluster
ILMN_1701603	NM_000478.3	ALPL	1.60	3.02	0.0398	1	Homo sapiens alkaline phosphatase. liver/bone/kidney (ALPL). transcript variant 1. mRNA.	Up_Regulated
ILMN_1728639	NM_000570.3	FCGR3B	1.46	2.75	0.0227	1	Homo sapiens Fc fragment of IgG. low affinity IIIb. receptor (CD16b) (FCGR3B). mRNA.	Up_Regulated
ILMN_3236130	XR_039468.1	LOC100132547	1.44	2.70	0.0227	12	PREDICTED: Homo sapiens misc_RNA (LOC100132547). miscRNA.	Up_Regulated
ILMN_1726460	NM_001034996.1	RPL14	1.32	2.50	0.0258	3	Homo sapiens ribosomal protein L14 (RPL14). transcript variant 1. mRNA.	Up_Regulated
ILMN_3199929	XR_018242.2	LOC390183	1.23	2.35	0.0324		PREDICTED: Homo sapiens misc_RNA (LOC390183). miscRNA.	Up_Regulated
ILMN_3207738	XR_016632.2	LOC646527	1.22	2.32	0.0434	13	PREDICTED: Homo sapiens misc_RNA (LOC646527). miscRNA.	Up_Regulated
ILMN_3294222	XR_039018.1	LOC100132673	1.20	2.30	0.0432		PREDICTED: Homo sapiens misc_RNA (LOC100132673). miscRNA.	Up_Regulated
ILMN_3261938	XM_001717333.1	LOC100130154	1.18	2.26	0.0227	2	PREDICTED: Homo sapiens similar to thymosin. beta 10 (LOC100130154). mRNA.	Up_Regulated
ILMN_3286813	XR_019605.1	LOC391019	1.13	2.19	0.0164		PREDICTED: Homo sapiens misc_RNA (LOC391019). miscRNA.	Up_Regulated
ILMN_1683044	NM_006241.3	PPP1R2	1.10	2.15	0.0233	3	Homo sapiens protein phosphatase 1. regulatory (inhibitor) subunit 2 (PPP1R2). mRNA.	Up_Regulated
ILMN_1654504	NM_197954.2	CLEC7A	1.04	2.06	0.0398	12	Homo sapiens C-type lectin domain family 7. member A (CLEC7A). transcript variant 6. mRNA.	Up_Regulated
ILMN_1808837	XR_017397.1	LOC644029	1.04	2.06	0.0432	15	PREDICTED: Homo sapiens similar to 60S ribosomal protein L7a (LOC644029). mRNA.	Up_Regulated
ILMN_2091846	NR_002200.1	FTHL2	1.04	2.05	0.0164	1	Homo sapiens ferritin. heavy polypeptide-like 2 (FTHL2) on chromosome 1.	Up_Regulated
ILMN_3199974	XR_039693.1	LOC100131787	1.02	2.03	0.0324	1	PREDICTED: Homo sapiens misc_RNA (LOC100131787). miscRNA.	Up_Regulated
ILMN_2145250	NR_002182.1	NACAP1	1.01	2.02	0.0389	8	Homo sapiens nascent-polypeptide-associated complex alpha polypeptide pseudogene 1 (NACAP1). non-coding RNA.	Up_Regulated
ILMN_3280496	XR_038945.1	LOC100131526	1.01	2.01	0.0353		PREDICTED: Homo sapiens misc_RNA (LOC100131526). miscRNA.	Up_Regulated
ILMN_2166534	NM_004226.2	STK17B	1.01	2.01	0.0280	2	Homo sapiens serine/threonine kinase 17b (STK17B). mRNA.	Up_Regulated
ILMN_1796830	NM_198157.1	UBE2L3	0.98	1.97	0.0370	22	Homo sapiens ubiquitin-conjugating enzyme E2L 3 (UBE2L3). transcript variant 2. mRNA.	Up_Regulated
ILMN_2091123	NR_001318.1	HCG2P7	0.97	1.96	0.0434	6	Homo sapiens HLA complex group 2 pseudogene 7 (HCG2P7). non-coding RNA.	Up_Regulated
ILMN_1677814	NM_003786.2	ABCC3	0.97	1.96	0.0404	17	Homo sapiens ATP-binding cassette. sub-family C (CFTR/MRP). member 3 (ABCC3). mRNA.	Up_Regulated
ILMN_3282174	XR_038043.1	LOC646688	0.97	1.95	0.0096	1	PREDICTED: Homo sapiens misc_RNA (LOC646688). miscRNA.	Up_Regulated
ILMN_1798448	NM_006123.2	IDS	0.92	1.89	0.0479	X	Homo sapiens iduronate 2-sulfatase (Hunter syndrome) (IDS). transcript variant 2. mRNA.	Up_Regulated
ILMN_1766221	NM_001497.2	B4GALT1	0.91	1.88	0.0258	9	Homo sapiens UDP-Gal:betaGlcNAc beta 1.4- galactosyltransferase. polypeptide 1 (B4GALT1). mRNA.	Up_Regulated
ILMN_3264073	XM_001723889.1	LOC100130070	0.91	1.88	0.0227		PREDICTED: Homo sapiens similar to metallopanstimulin (LOC100130070). mRNA.	Up_Regulated
ILMN_3188076	XM_001723512.1	LOC100128060	0.91	1.88	0.0324		PREDICTED: Homo sapiens similar to mCG19129. transcript variant 2 (LOC100128060). mRNA.	Up_Regulated
ILMN_3257030	XR_016025.2	FTHL16	0.88	1.85	0.0178		PREDICTED: Homo sapiens misc_RNA (FTHL16). miscRNA.	Up_Regulated
ILMN_2112580	NM_000569.6	FCGR3A	0.88	1.84	0.0473	1	Homo sapiens Fc fragment of IgG. low affinity IIIa. receptor (CD16a) (FCGR3A). transcript variant 1. mRNA.	Up_Regulated
ILMN_3305397	XR_015391.1	LOC728843	0.86	1.82	0.0432	5	PREDICTED: Homo sapiens misc_RNA (LOC728843). miscRNA.	Up_Regulated
ILMN_3208014	XR_038146.1	LOC100131866	0.86	1.82	0.0318	1	PREDICTED: Homo sapiens misc_RNA (LOC100131866). miscRNA.	Up_Regulated
ILMN_3243744	NM_018453.3	EAPP	0.85	1.81	0.0398	14	Homo sapiens E2F-associated phosphoprotein (EAPP). mRNA.	Up_Regulated
ILMN_2337655	NM_004184.3	WARS	0.85	1.80	0.0319	14	Homo sapiens tryptophanyl-tRNA synthetase (WARS). transcript variant 1. mRNA.	Up_Regulated
ILMN_3214256	XM_001725191.1	LOC128192	0.84	1.79	0.0227		PREDICTED: Homo sapiens hypothetical LOC128192 (LOC128192). mRNA.	Up_Regulated
ILMN_1655827	NM_004236.2	COPS2	0.83	1.78	0.0280	15	Homo sapiens COP9 constitutive photomorphogenic homolog subunit 2 (Arabidopsis) (COPS2). mRNA.	Up_Regulated
ILMN_3201216	XR_038097.1	LOC441550	0.83	1.78	0.0250		PREDICTED: Homo sapiens misc_RNA (LOC441550). miscRNA.	Up_Regulated
ILMN_2244547	NM_001039481.1	ETNK1	0.82	1.77	0.0487	12	Homo sapiens ethanolamine kinase 1 (ETNK1). transcript variant 2. mRNA.	Up_Regulated
ILMN_3229652	XM_001721287.1	LOC728953	0.82	1.76	0.0227		PREDICTED: Homo sapiens similar to S19 ribosomal protein (LOC728953). mRNA.	Up_Regulated
ILMN_3289171	XM_001725183.1	LOC100131572	0.82	1.76	0.0336	1	PREDICTED: Homo sapiens similar to hCG1783679 (LOC100131572). mRNA.	Up_Regulated
ILMN_1722662	NM_002874.3	RAD23B	0.79	1.73	0.0438	9	Homo sapiens RAD23 homolog B (S. cerevisiae) (RAD23B). mRNA.	Up_Regulated
ILMN_3301052	XR_015547.2	LOC728791	0.77	1.71	0.0306		PREDICTED: Homo sapiens misc_RNA (LOC728791). miscRNA.	Up_Regulated
ILMN_3280020	XR_017565.2	LOC441506	0.77	1.70	0.0441	16	PREDICTED: Homo sapiens misc_RNA (LOC441506). miscRNA.	Up_Regulated
ILMN_1760089	XM_930178.1	LOC645018	0.76	1.70	0.0364	4	PREDICTED: Homo sapiens similar to ribosomal protein S2 (LOC645018). mRNA.	Up_Regulated
ILMN_1777286	XM_371023.4	LOC388344	0.76	1.69	0.0364	17	PREDICTED: Homo sapiens similar to ribosomal protein L13. transcript variant 1 (LOC388344). mRNA.	Up_Regulated
ILMN_3243859	NM_005005.2	NDUFB9	0.76	1.69	0.0227	8	Homo sapiens NADH dehydrogenase (ubiquinone) 1 beta subcomplex. 9. 22kDa (NDUFB9). mRNA.	Up_Regulated
ILMN_1755909	NM_017896.2	C20ORF11	0.75	1.69	0.0336	20	Homo sapiens chromosome 20 open reading frame 11 (C20orf11). mRNA.	Up_Regulated
ILMN_1724230	XM_943005.2	LOC642236	0.74	1.67	0.0339		PREDICTED: Homo sapiens similar to FRG1 protein (FSHD region gene 1 protein). transcript variant 8 (LOC642236). mRNA	Up_Regulated
ILMN_1679045	NM_016038.2	SBDS	0.74	1.67	0.0227	7	Homo sapiens Shwachman-Bodian-Diamond syndrome (SBDS). mRNA.	Up_Regulated
ILMN_1767892	NM_007240.1	DUSP12	0.74	1.67	0.0443	1	Homo sapiens dual specificity phosphatase 12 (DUSP12). mRNA.	Up_Regulated
ILMN_3271244	XR_042325.1	LOC100130775	0.74	1.67	0.0406	12	PREDICTED: Homo sapiens misc_RNA (LOC100130775). miscRNA.	Up_Regulated
ILMN_1659843	NM_006260.2	DNAJC3	0.74	1.67	0.0407	13	Homo sapiens DnaJ (Hsp40) homolog. subfamily C. member 3 (DNAJC3). mRNA.	Up_Regulated
ILMN_1807925	NM_053064.3	GNG2	0.74	1.66	0.0227	14	Homo sapiens guanine nucleotide binding protein (G protein). gamma 2 (GNG2). mRNA.	Up_Regulated
ILMN_3305475	XM_001725700.1	LOC729708	0.73	1.66	0.0210		PREDICTED: Homo sapiens similar to rcTPI1. transcript variant 1 (LOC729708). mRNA.	Up_Regulated
ILMN_2298511	NM_001031827.1	BOLA2	0.73	1.66	0.0353	16	Homo sapiens bolA homolog 2 (E. coli) (BOLA2). mRNA.	Up_Regulated
ILMN_1770667	NM_016217.2	HECA	0.73	1.66	0.0454	6	Homo sapiens headcase homolog (Drosophila) (HECA). mRNA.	Up_Regulated
ILMN_2055700	NM_006527.2	SLBP	0.73	1.66	0.0404	4	Homo sapiens stem-loop binding protein (SLBP). mRNA.	Up_Regulated
ILMN_1685378	XR_015809.1	LOC728973	0.72	1.65	0.0236	1	PREDICTED: Homo sapiens similar to 40S ribosomal protein S7 (S8) (LOC728973). mRNA.	Up_Regulated
ILMN_3242120	NM_001089704.3	RAP1BL	0.72	1.65	0.0403		Homo sapiens hCG1757335 (RAP1BL). mRNA.	Up_Regulated
ILMN_1769665	NM_201434.1	RAB5C	0.72	1.65	0.0292	17	Homo sapiens RAB5C. member RAS oncogene family (RAB5C). transcript variant 1. mRNA.	Up_Regulated
ILMN_1674024	NM_022466.3	IKZF5	0.72	1.64	0.0227		Homo sapiens IKAROS family zinc finger 5 (Pegasus) (IKZF5). mRNA.	Up_Regulated
ILMN_3290385	XR_017380.2	LOC401640	0.71	1.64	0.0371	10	PREDICTED: Homo sapiens misc_RNA (LOC401640). miscRNA.	Up_Regulated
ILMN_1659976	NM_006831.1	CLP1	0.71	1.64	0.0432	11	Homo sapiens CLP1. cleavage and polyadenylation factor I subunit. homolog (S. cerevisiae) (CLP1). mRNA.	Up_Regulated
ILMN_1654609	NM_053000.1	TIGA1	0.71	1.63	0.0382	5	Homo sapiens TIGA1 (TIGA1). mRNA.	Up_Regulated
ILMN_1678919	NM_018566.3	YOD1	0.71	1.63	0.0404	1	Homo sapiens YOD1 OTU deubiquinating enzyme 1 homolog (S. cerevisiae) (YOD1). mRNA.	Up_Regulated
ILMN_1661174	XM_001133089.1	LOC731640	0.70	1.63	0.0495		PREDICTED: Homo sapiens similar to 60S ribosomal protein L21. transcript variant 2 (LOC731640). mRNA.	Up_Regulated
ILMN_2133784	NM_212555.1	PATE2	0.70	1.62	0.0227	11	Homo sapiens prostate and testis expressed 2 (PATE2). mRNA.	Up_Regulated
ILMN_1811636	NM_018010.2	IFT57	0.70	1.62	0.0171	3	Homo sapiens intraflagellar transport 57 homolog (Chlamydomonas) (IFT57). mRNA.	Up_Regulated
ILMN_1678808	NM_014924.3	KIAA0831	0.70	1.62	0.0364	14	Homo sapiens KIAA0831 (KIAA0831). mRNA.	Up_Regulated
ILMN_1678170	NM_000902.3	MME	0.69	1.62	0.0254	3	Homo sapiens membrane metallo-endopeptidase (MME). transcript variant 1. mRNA.	Up_Regulated
ILMN_1722532	NM_018433.3	JMJD1A	0.68	1.61	0.0443	2	Homo sapiens jumonji domain containing 1A (JMJD1A). mRNA.	Up_Regulated
ILMN_1680347	NM_020933.2	ZNF317	0.68	1.60	0.0280	19	Homo sapiens zinc finger protein 317 (ZNF317). mRNA.	Up_Regulated
ILMN_3267760	XM_001721819.1	LOC100128936	0.68	1.60	0.0331		PREDICTED: Homo sapiens similar to ribosomal protein L10a (LOC100128936). mRNA.	Up_Regulated
ILMN_1755808	XM_942669.1	LOC654194	0.67	1.60	0.0429		PREDICTED: Homo sapiens similar to ribosomal protein S27 (LOC654194). mRNA.	Up_Regulated
ILMN_1692545	XM_933970.1	LOC646849	0.67	1.59	0.0364	3	PREDICTED: Homo sapiens hypothetical protein LOC646849 (LOC646849). mRNA.	Up_Regulated
ILMN_1659411	NM_032940.2	POLR2C	0.67	1.59	0.0324	16	Homo sapiens polymerase (RNA) II (DNA directed) polypeptide C. 33kDa (POLR2C). mRNA.	Up_Regulated
ILMN_3225591	XR_041286.1	RPL14L	0.66	1.58	0.0182		PREDICTED: Homo sapiens misc_RNA (RPL14L). miscRNA.	Up_Regulated
ILMN_1675462	NM_007080.2	LSM6	0.66	1.58	0.0432	4	Homo sapiens LSM6 homolog. U6 small nuclear RNA associated (S. cerevisiae) (LSM6). mRNA.	Up_Regulated
ILMN_1745904	NM_005436.2	CCDC6	0.66	1.58	0.0210	10	Homo sapiens coiled-coil domain containing 6 (CCDC6). mRNA.	Up_Regulated
ILMN_3235216	NM_174887.2	IFT20	0.66	1.58	0.0336	17	Homo sapiens intraflagellar transport 20 homolog (Chlamydomonas) (IFT20). mRNA.	Up_Regulated
ILMN_1743352	NM_003192.2	TBCC	0.65	1.57	0.0319	6	Homo sapiens tubulin folding cofactor C (TBCC). mRNA.	Up_Regulated
ILMN_1813344	NM_024120.3	C20ORF7	0.65	1.57	0.0434	20	Homo sapiens chromosome 20 open reading frame 7 (C20orf7). transcript variant 1. mRNA.	Up_Regulated
ILMN_3290577	XR_038761.1	LOC391833	0.65	1.57	0.0339		PREDICTED: Homo sapiens misc_RNA (LOC391833). miscRNA.	Up_Regulated
ILMN_1756779	NM_004859.3	CLTC	0.65	1.57	0.0434	17	Homo sapiens clathrin. heavy chain (Hc) (CLTC). mRNA.	Up_Regulated
ILMN_1804656	NM_032901.2	C12ORF62	0.65	1.56	0.0375	12	Homo sapiens chromosome 12 open reading frame 62 (C12orf62). mRNA.	Up_Regulated
ILMN_3276209	XR_038568.1	LOC727865	0.64	1.56	0.0230		PREDICTED: Homo sapiens misc_RNA (LOC727865). miscRNA.	Up_Regulated
ILMN_1766171	NM_007241.2	SNF8	0.64	1.56	0.0280	17	Homo sapiens SNF8. ESCRT-II complex subunit. homolog (S. cerevisiae) (SNF8). mRNA.	Up_Regulated
ILMN_3227563	XR_016047.1	LOC729255	0.64	1.56	0.0286		PREDICTED: Homo sapiens misc_RNA (LOC729255). miscRNA.	Up_Regulated
ILMN_1758311	NM_001047160.1	NET1	0.64	1.56	0.0327	10	Homo sapiens neuroepithelial cell transforming 1 (NET1). transcript variant 1. mRNA.	Up_Regulated
ILMN_3202396	XM_001723015.1	LOC390735	0.63	1.55	0.0371		PREDICTED: Homo sapiens hCG1642987 (LOC390735). mRNA.	Up_Regulated
ILMN_3252446	XM_001715065.1	LOC100129543	0.63	1.55	0.0429		PREDICTED: Homo sapiens hypothetical protein LOC100129543 (LOC100129543). mRNA.	Up_Regulated
ILMN_2134453	NM_000570.2	FCGR3B	0.63	1.55	0.0434	1	Homo sapiens Fc fragment of IgG. low affinity IIIb. receptor (CD16b) (FCGR3B). mRNA.	Up_Regulated
ILMN_2324998	NM_001042635.1	NGDN	0.63	1.54	0.0164	14	Homo sapiens neuroguidin. EIF4E binding protein (NGDN). transcript variant 1. mRNA.	Up_Regulated
ILMN_1736806	NM_018440.3	PAG1	0.63	1.54	0.0429	8	Homo sapiens phosphoprotein associated with glycosphingolipid microdomains 1 (PAG1). mRNA.	Up_Regulated
ILMN_1663090	NM_032195.1	SON	0.62	1.53	0.0367	21	Homo sapiens SON DNA binding protein (SON). transcript variant b. mRNA.	Up_Regulated
ILMN_1659553	NM_022662.2	ANAPC1	0.62	1.53	0.0404	2	Homo sapiens anaphase promoting complex subunit 1 (ANAPC1). mRNA.	Up_Regulated
ILMN_1790741	NM_194460.1	RNF126	0.62	1.53	0.0171	19	Homo sapiens ring finger protein 126 (RNF126). mRNA.	Up_Regulated
ILMN_1715661	NM_003201.1	TFAM	0.61	1.53	0.0287	10	Homo sapiens transcription factor A. mitochondrial (TFAM). nuclear gene encoding mitochondrial protein. mRNA.	Up_Regulated
ILMN_1737813	XM_001132636.1	PRNPIP	0.61	1.53	0.0164		PREDICTED: Homo sapiens prion protein interacting protein (PRNPIP). mRNA.	Up_Regulated
ILMN_1686152	NM_015044.3	GGA2	0.61	1.52	0.0406	16	Homo sapiens golgi associated. gamma adaptin ear containing. ARF binding protein 2 (GGA2). mRNA.	Up_Regulated
ILMN_1740319	NM_032036.2	IFI27L2	0.61	1.52	0.0164	14	Homo sapiens interferon. alpha-inducible protein 27-like 2 (IFI27L2). mRNA.	Up_Regulated
ILMN_3279675	XM_942039.3	LOC388339	0.61	1.52	0.0227		PREDICTED: Homo sapiens similar to ribosomal protein. transcript variant 4 (LOC388339). mRNA.	Up_Regulated

ILMN_1684114	NR_002187.2	LOC286016	0.61	1.52	0.0227	7	Homo sapiens triosephosphate isomerase 1 pseudogene (LOC286016). non-coding RNA.	Up_Regulated
ILMN_1768913	NM_004622.2	TSN	0.61	1.52	0.0227	2	Homo sapiens translin (TSN). mRNA.	Up_Regulated
ILMN_1762294	NM_025008.3	ADAMTSL4	0.61	1.52	0.0317	1	Homo sapiens ADAMTS-like 4 (ADAMTSL4). transcript variant 2. mRNA.	Up_Regulated
ILMN_3234116	XM_001133471.2	LOC730382	0.60	1.52	0.0227	7	PREDICTED: Homo sapiens hypothetical LOC730382 (LOC730382). mRNA.	Up_Regulated
ILMN_2365544	NM_001034833.1	NHP2	0.60	1.52	0.0287	5	Homo sapiens NHP2 ribonucleoprotein homolog (yeast) (NHP2). transcript variant 2. mRNA.	Up_Regulated
ILMN_1727495	NM_032438.1	L3MBTL3	0.60	1.52	0.0172	6	Homo sapiens l(3)mbt-like 3 (Drosophila) (L3MBTL3). transcript variant 1. mRNA.	Up_Regulated
ILMN_1782611	XM_927140.1	LOC643870	0.60	1.51	0.0227	6	PREDICTED: Homo sapiens similar to Translationally-controlled tumor protein (TCTP) (p23) (Histamine-releasing factor) (	Up_Regulated
ILMN_1728799	NM_000507.2	FBP1	0.59	1.51	0.0413	9	Homo sapiens fructose-1.6-bisphosphatase 1 (FBP1). mRNA.	Up_Regulated
ILMN_1654289	NM_005229.2	ELK1	0.59	1.50	0.0451	X	Homo sapiens ELK1. member of ETS oncogene family (ELK1). mRNA.	Up_Regulated
ILMN_1676792	XM_928168.1	LOC645138	0.59	1.50	0.0321	16	PREDICTED: Homo sapiens similar to ribosomal protein S11 (LOC645138). mRNA.	Up_Regulated
ILMN_2409078	NR_001459.2	SNHG10	0.59	1.50	0.0228	14	Homo sapiens small nucleolar RNA host gene 10 (non-protein coding) (SNHG10). non-coding RNA.	Up_Regulated
ILMN_2397954	NM_005485.3	PARP3	0.58	1.49	0.0432	3	Homo sapiens poly (ADP-ribose) polymerase family. member 3 (PARP3). transcript variant 2. mRNA.	Up_Regulated
ILMN_2098437	NR_002183.1	FAM10A4	0.57	1.49	0.0315	13	Homo sapiens family with sequence similarity 10. member A4 pseudogene (FAM10A4). non-coding RNA.	Up_Regulated
ILMN_3285410	XR_039794.1	LOC642738	0.57	1.49	0.0488		PREDICTED: Homo sapiens misc_RNA (LOC642738). miscRNA.	Up_Regulated
ILMN_1678729	NM_001037633.1	SIL1	0.57	1.48	0.0257	5	Homo sapiens SIL1 homolog. endoplasmic reticulum chaperone (S. cerevisiae) (SIL1). transcript variant 1. mRNA.	Up_Regulated
ILMN_1690295	XR_018327.1	LOC648343	0.57	1.48	0.0210		PREDICTED: Homo sapiens similar to protein phosphatase 1 regulatory subunit 14B (LOC648343). mRNA.	Up_Regulated
ILMN_2078264	NM_173824.2	C3ORF38	0.57	1.48	0.0388	3	Homo sapiens chromosome 3 open reading frame 38 (C3orf38). mRNA.	Up_Regulated
ILMN_3291413	XR_017293.1	LOC646672	0.57	1.48	0.0353	16	PREDICTED: Homo sapiens misc_RNA (LOC646672). miscRNA.	Up_Regulated
ILMN_2160160	NR_000016.1	SNORD36C	0.57	1.48	0.0353	9	Homo sapiens small nucleolar RNA. C/D box 36C (SNORD36C). small nucleolar RNA.	Up_Regulated
ILMN_1721762	NM_003853.2	IL18RAP	0.56	1.48	0.0389	2	Homo sapiens interleukin 18 receptor accessory protein (IL18RAP). mRNA.	Up_Regulated
ILMN_1724700	NM_003831.3	RIOK3	0.56	1.48	0.0227	18	Homo sapiens RIO kinase 3 (yeast) (RIOK3). mRNA.	Up_Regulated
ILMN_1776653	NM_001037540.1	SCML1	0.56	1.48	0.0299	X	Homo sapiens sex comb on midleg-like 1 (Drosophila) (SCML1). transcript variant 1. mRNA.	Up_Regulated
ILMN_3203515	XR_016218.2	LOC401717	0.56	1.48	0.0227	12	PREDICTED: Homo sapiens misc_RNA (LOC401717). miscRNA.	Up_Regulated
ILMN_1718672	NM_001034833.1	NHP2	0.56	1.47	0.0249	5	Homo sapiens NHP2 ribonucleoprotein homolog (yeast) (NHP2). transcript variant 2. mRNA.	Up_Regulated
ILMN_2181125	NM_022080.1	NAPB	0.56	1.47	0.0324	20	Homo sapiens N-ethylmaleimide-sensitive factor attachment protein. beta (NAPB). mRNA.	Up_Regulated
ILMN_1680703	NM_031280.2	MRPS15	0.56	1.47	0.0282	1	Homo sapiens mitochondrial ribosomal protein S15 (MRPS15). nuclear gene encoding mitochondrial protein. mRNA.	Up_Regulated
ILMN_1701434	NM_015646.4	RAP1B	0.55	1.47	0.0331	12	Homo sapiens RAP1B. member of RAS oncogene family (RAP1B). transcript variant 1. mRNA.	Up_Regulated
ILMN_2175465	NM_016304.2	RSL24D1	0.55	1.47	0.0432	15	Homo sapiens ribosomal L24 domain containing 1 (RSL24D1). mRNA.	Up_Regulated
ILMN_1733356	NM_199482.1	PREI3	0.55	1.46	0.0227	2	Homo sapiens preimplantation protein 3 (PREI3). transcript variant 2. mRNA.	Up_Regulated
ILMN_1710697	NM_003910.2	BUD31	0.55	1.46	0.0353	7	Homo sapiens BUD31 homolog (S. cerevisiae) (BUD31). mRNA.	Up_Regulated
ILMN_1726308	NR_002183.1	FAM10A4	0.54	1.45	0.0441	13	Homo sapiens family with sequence similarity 10. member A4 pseudogene (FAM10A4). non-coding RNA.	Up_Regulated
ILMN_1703226	NM_138417.2	KTI12	0.54	1.45	0.0439	1	Homo sapiens KTI12 homolog. chromatin associated (S. cerevisiae) (KTI12). mRNA.	Up_Regulated
ILMN_1728355	NM_002810.2	PSMD4	0.53	1.45	0.0485	1	Homo sapiens proteasome (prosome. macropain) 26S subunit. non-ATPase. 4 (PSMD4). mRNA.	Up_Regulated
ILMN_2156982	NM_033416.1	IMP4	0.53	1.44	0.0280	2	Homo sapiens IMP4. U3 small nucleolar ribonucleoprotein. homolog (yeast) (IMP4). mRNA.	Up_Regulated
ILMN_1795007	NM_024520.1	C2ORF47	0.52	1.44	0.0429	2	Homo sapiens chromosome 2 open reading frame 47 (C2orf47). mRNA.	Up_Regulated
ILMN_1686811	XM_938297.1	LOC402644	0.52	1.44	0.0280		PREDICTED: Homo sapiens similar to peptidylprolyl isomerase A isoform 1 (LOC402644). mRNA.	Up_Regulated
ILMN_1756139	XM_926656.1	LOC643310	0.52	1.44	0.0287	X	PREDICTED: Homo sapiens similar to heat shock 70kD protein binding protein (LOC643310). mRNA.	Up_Regulated
ILMN_3247882	NM_024066.1	ERI3	0.52	1.44	0.0338	1	Homo sapiens ERI1 exoribonuclease family member 3 (ERI3). mRNA.	Up_Regulated
ILMN_1680419	NM_024708.2	ASB7	0.52	1.44	0.0227	15	Homo sapiens ankyrin repeat and SOCS box-containing 7 (ASB7). transcript variant 1. mRNA.	Up_Regulated
ILMN_1788062	NM_003025.2	SH3GL1	0.52	1.44	0.0324	19	Homo sapiens SH3-domain GRB2-like 1 (SH3GL1). mRNA.	Up_Regulated
ILMN_1761801	NR_003148.2	LOC147804	0.52	1.44	0.0446	19	Homo sapiens tropomyosin 3 pseudogene (LOC147804). non-coding RNA.	Up_Regulated
ILMN_1759030	NM_198794.1	MAP4K5	0.52	1.44	0.0428	14	Homo sapiens mitogen-activated protein kinase kinase kinase kinase 5 (MAP4K5). transcript variant 2. mRNA.	Up_Regulated
ILMN_1734696	NM_004477.2	FRG1	0.52	1.43	0.0252	4	Homo sapiens FSHD region gene 1 (FRG1). mRNA.	Up_Regulated
ILMN_1769277	XM_940587.1	LOC651436	0.52	1.43	0.0287		PREDICTED: Homo sapiens similar to ribosomal protein L9 (LOC651436). mRNA.	Up_Regulated
ILMN_2407529	NM_197939.1	RNF135	0.52	1.43	0.0164	17	Homo sapiens ring finger protein 135 (RNF135). transcript variant 2. mRNA.	Up_Regulated
ILMN_1690386	NM_020536.2	CSRP2BP	0.51	1.43	0.0403	20	Homo sapiens CSRP2 binding protein (CSRP2BP). transcript variant 1. mRNA.	Up_Regulated
ILMN_1799516	NM_015190.3	DNAJC9	0.51	1.43	0.0258	10	Homo sapiens DnaJ (Hsp40) homolog. subfamily C. member 9 (DNAJC9). mRNA.	Up_Regulated
ILMN_3306168	NM_199482.2	MOBKL3	0.50	1.42	0.0258	2	Homo sapiens MOB1. Mps One Binder kinase activator-like 3 (yeast) (MOBKL3). transcript variant 2. mRNA.	Up_Regulated
ILMN_1817255	BX537514	HS.92308	0.50	1.42	0.0164	12	Homo sapiens mRNA; cDNA DKFZp313N0919 (from clone DKFZp313N0919)	Up_Regulated
ILMN_3225669	XR_016556.2	RPS10P3	0.50	1.41	0.0227	9	PREDICTED: Homo sapiens misc_RNA (RPS10P3). miscRNA.	Up_Regulated
ILMN_1758474	NM_003690.3	PRKRA	0.50	1.41	0.0485	2	Homo sapiens protein kinase. interferon-inducible double stranded RNA dependent activator (PRKRA). mRNA.	Up_Regulated
ILMN_2098325	NM_023080.1	C8ORF33	0.50	1.41	0.0385	8	Homo sapiens chromosome 8 open reading frame 33 (C8orf33). mRNA.	Up_Regulated
ILMN_1752285	NM_000968.2	RPL4	0.50	1.41	0.0413	15	Homo sapiens ribosomal protein L4 (RPL4). mRNA.	Up_Regulated
ILMN_1795856	XM_928022.1	LOC644935	0.50	1.41	0.0394	2	PREDICTED: Homo sapiens similar to E74-like factor 2 (ets domain transcription factor) isoform 1 (LOC644935). mRNA.	Up_Regulated
ILMN_1751258	NM_002489.2	NDUFA4	0.50	1.41	0.0227	7	Homo sapiens NADH dehydrogenase (ubiquinone) 1 alpha subcomplex. 4. 9kDa (NDUFA4). nuclear gene encoding mitoc	Up_Regulated
ILMN_3215715	XR_017251.2	LOC389386	0.50	1.41	0.0437	6	PREDICTED: Homo sapiens misc_RNA (LOC389386). partial miscRNA.	Up_Regulated
ILMN_1660812	NM_018453.2	C14ORF11	0.49	1.41	0.0227	14	Homo sapiens chromosome 14 open reading frame 11 (C14orf11). mRNA.	Up_Regulated
ILMN_2222234	NM_006406.1	PRDX4	0.49	1.41	0.0227	X	Homo sapiens peroxiredoxin 4 (PRDX4). mRNA.	Up_Regulated
ILMN_1779401	NM_007236.3	CHP	0.49	1.41	0.0491	15	Homo sapiens calcium binding protein P22 (CHP). mRNA.	Up_Regulated
ILMN_2358560	NM_001010927.2	TIAM2	0.49	1.40	0.0280	6	Homo sapiens T-cell lymphoma invasion and metastasis 2 (TIAM2). transcript variant 2. mRNA.	Up_Regulated
ILMN_2367070	NM_001037171.1	ACOT9	0.49	1.40	0.0339	X	Homo sapiens acyl-CoA thioesterase 9 (ACOT9). transcript variant 1. mRNA.	Up_Regulated
ILMN_1689652	NM_018146.2	RNMTL1	0.47	1.39	0.0497	17	Homo sapiens RNA methyltransferase like 1 (RNMTL1). mRNA.	Up_Regulated
ILMN_2155172	NM_018321.3	BRIX1	0.47	1.39	0.0254	5	Homo sapiens BRX1. biogenesis of ribosomes. homolog (S. cerevisiae) (BRIX1). mRNA.	Up_Regulated
ILMN_1766247	NM_020861.1	ZBTB2	0.47	1.39	0.0299	6	Homo sapiens zinc finger and BTB domain containing 2 (ZBTB2). mRNA.	Up_Regulated
ILMN_1709728	NM_022902.2	SLC30A5	0.47	1.38	0.0463	5	Homo sapiens solute carrier family 30 (zinc transporter). member 5 (SLC30A5). transcript variant 1. mRNA.	Up_Regulated
ILMN_3241051	NM_001090027.1	LOC644907	0.47	1.38	0.0404	7	Homo sapiens hCG18290 (LOC644907). mRNA.	Up_Regulated
ILMN_1713482	NM_016403.3	CWC15	0.46	1.38	0.0407	11	Homo sapiens CWC15 spliceosome-associated protein homolog (S. cerevisiae) (CWC15). mRNA.	Up_Regulated
ILMN_3215381	XR_017268.2	LOC645175	0.46	1.38	0.0227	6	PREDICTED: Homo sapiens misc_RNA (LOC645175). miscRNA.	Up_Regulated
ILMN_2042771	NM_004219.2	PTTG1	0.46	1.37	0.0315	5	Homo sapiens pituitary tumor-transforming 1 (PTTG1). mRNA.	Up_Regulated
ILMN_1801913	NM_006347.3	PPIH	0.46	1.37	0.0394	1	Homo sapiens peptidylprolyl isomerase H (cyclophilin H) (PPIH). mRNA.	Up_Regulated
ILMN_3243890	NM_002488.3	NDUFA2	0.46	1.37	0.0258	5	Homo sapiens NADH dehydrogenase (ubiquinone) 1 alpha subcomplex. 2. 8kDa (NDUFA2). mRNA.	Up_Regulated
ILMN_1734486	NM_052965.1	C1ORF19	0.46	1.37	0.0445	1	Homo sapiens chromosome 1 open reading frame 19 (C1orf19). mRNA.	Up_Regulated
ILMN_1805998	NM_031446.3	C18ORF21	0.45	1.37	0.0336	18	Homo sapiens chromosome 18 open reading frame 21 (C18orf21). mRNA.	Up_Regulated
ILMN_1767894	NM_002690.1	POLB	0.45	1.37	0.0299	8	Homo sapiens polymerase (DNA directed). beta (POLB). mRNA.	Up_Regulated
ILMN_3229859	NM_001100819.1	MOBKL3	0.45	1.37	0.0233	2	Homo sapiens MOB1. Mps One Binder kinase activator-like 3 (yeast) (MOBKL3). transcript variant 3. mRNA.	Up_Regulated
ILMN_3211302	XR_039057.1	LOC646909	0.45	1.36	0.0336		PREDICTED: Homo sapiens misc_RNA (LOC646909). miscRNA.	Up_Regulated
ILMN_1800311	NM_004506.2	HSF2	0.44	1.36	0.0470	6	Homo sapiens heat shock transcription factor 2 (HSF2). mRNA.	Up_Regulated
ILMN_1759184	NM_199250.1	C19ORF48	0.44	1.36	0.0404	19	Homo sapiens chromosome 19 open reading frame 48 (C19orf48). mRNA.	Up_Regulated
ILMN_1746525	NR_002200.1	FTHL2	0.44	1.36	0.0434	1	Homo sapiens ferritin. heavy polypeptide-like 2 (FTHL2) on chromosome 1.	Up_Regulated
ILMN_1795639	NM_002412.2	MGMT	0.44	1.36	0.0172	10	Homo sapiens O-6-methylguanine-DNA methyltransferase (MGMT). mRNA.	Up_Regulated
ILMN_1731107	NM_025140.1	CCDC92	0.44	1.36	0.0445	12	Homo sapiens coiled-coil domain containing 92 (CCDC92). mRNA.	Up_Regulated
ILMN_3256926	XM_001723713.1	LOC100130764	0.44	1.36	0.0353	8	PREDICTED: Homo sapiens p150-like (LOC100130764). mRNA.	Up_Regulated
ILMN_1696066	NM_001014438.1	CARS	0.44	1.36	0.0299	11	Homo sapiens cysteinyl-tRNA synthetase (CARS). transcript variant 4. mRNA.	Up_Regulated
ILMN_3300891	XR_040489.1	LOC729652	0.44	1.35	0.0324		PREDICTED: Homo sapiens hypothetical protein LOC729652 (LOC729652). miscRNA.	Up_Regulated
ILMN_3277297	XR_018378.2	LOC391825	0.44	1.35	0.0249		PREDICTED: Homo sapiens similar to hCG1643032 (LOC391825). mRNA.	Up_Regulated
ILMN_1704305	NM_016101.3	NIP7	0.43	1.35	0.0319	16	Homo sapiens nuclear import 7 homolog (S. cerevisiae) (NIP7). mRNA.	Up_Regulated
ILMN_1695899	XM_944469.1	LOC648659	0.43	1.35	0.0385		PREDICTED: Homo sapiens similar to ribosomal protein S3a. transcript variant 4 (LOC648659). mRNA.	Up_Regulated
ILMN_1773228	NM_001933.3	DLST	0.43	1.35	0.0443	14	Homo sapiens dihydrolipoamide S-succinyltransferase (E2 component of 2-oxo-glutarate complex) (DLST). mRNA.	Up_Regulated
ILMN_3238782	XR_018431.1	LOC728244	0.43	1.35	0.0434		PREDICTED: Homo sapiens misc_RNA (LOC728244). miscRNA.	Up_Regulated
ILMN_1783636	NM_004373.2	COX6A1	0.42	1.34	0.0227	12	Homo sapiens cytochrome c oxidase subunit VIa polypeptide 1 (COX6A1). nuclear gene encoding mitochondrial protein.	Up_Regulated
ILMN_2059535	NM_014634.2	PPM1F	0.42	1.34	0.0299	22	Homo sapiens protein phosphatase 1F (PP2C domain containing) (PPM1F). mRNA.	Up_Regulated
ILMN_1874613	AW979088	HS.163264	0.42	1.34	0.0230	18	EST391198 MAGE resequences. MAGP Homo sapiens cDNA. mRNA sequence	Up_Regulated
ILMN_2278653	NM_001076678.1	ZNF493	0.42	1.34	0.0470	19	Homo sapiens zinc finger protein 493 (ZNF493). transcript variant 3. mRNA.	Up_Regulated
ILMN_1770048	NM_001077394.1	DPH5	0.41	1.33	0.0315	1	Homo sapiens DPH5 homolog (S. cerevisiae) (DPH5). transcript variant 1. mRNA.	Up_Regulated
ILMN_3218538	XR_016204.2	LOC345645	0.41	1.33	0.0336	5	PREDICTED: Homo sapiens similar to hCG2002932 (LOC345645). mRNA.	Up_Regulated
ILMN_1730077	NM_152260.1	RPUSD2	0.41	1.33	0.0334	15	Homo sapiens RNA pseudouridylate synthase domain containing 2 (RPUSD2). mRNA.	Up_Regulated
ILMN_1794011	NM_004267.3	CHST2	0.41	1.33	0.0413	3	Homo sapiens carbohydrate (N-acetylglucosamine-6-O) sulfotransferase 2 (CHST2). mRNA.	Up_Regulated
ILMN_3210171	XR_019429.2	LOC389156	0.41	1.33	0.0469		PREDICTED: Homo sapiens misc_RNA (LOC389156). miscRNA.	Up_Regulated
ILMN_1797384	NM_000375.1	UROS	0.40	1.32	0.0331	10	Homo sapiens uroporphyrinogen III synthase (congenital erythropoietic porphyria) (UROS). mRNA.	Up_Regulated
ILMN_1673282	NM_002294.1	LAMP2	0.40	1.32	0.0319	X	Homo sapiens lysosomal-associated membrane protein 2 (LAMP2). transcript variant LAMP2A. mRNA.	Up_Regulated

ILMN_3244434	NM_015230.2	ARAP2	0.40	1.32	0.0404	4	Homo sapiens ArfGAP with RhoGAP domain. ankyrin repeat and PH domain 2 (ARAP2). mRNA.	Up_Regulated
ILMN_1710354	NM_005550.2	KIFC3	0.40	1.32	0.0469	16	Homo sapiens kinesin family member C3 (KIFC3). mRNA.	Up_Regulated
ILMN_1706571	NM_007001.1	SLC35D2	0.40	1.32	0.0364	9	Homo sapiens solute carrier family 35. member D2 (SLC35D2). mRNA.	Up_Regulated
ILMN_2215640	NM_080386.1	TUBA3D	0.39	1.31	0.0434	2	Homo sapiens tubulin. alpha 3d (TUBA3D). mRNA.	Up_Regulated
ILMN_1731720	NM_030815.2	PDRG1	0.39	1.31	0.0365	20	Homo sapiens p53 and DNA-damage regulated 1 (PDRG1). mRNA.	Up_Regulated
ILMN_1722583	NM_006067.3	COX4NB	0.39	1.31	0.0446	16	Homo sapiens COX4 neighbor (COX4NB). mRNA.	Up_Regulated
ILMN_3181695	XR_038380.1	LOC100130178	0.39	1.31	0.0434		PREDICTED: Homo sapiens misc_RNA (LOC100130178). miscRNA.	Up_Regulated
ILMN_3296519	XR_038902.1	LOC728002	0.39	1.31	0.0443		PREDICTED: Homo sapiens misc_RNA (LOC728002). miscRNA.	Up_Regulated
ILMN_1661170	NM_005004.2	NDUFB8	0.38	1.30	0.0227	10	Homo sapiens NADH dehydrogenase (ubiquinone) 1 beta subcomplex. 8. 19kDa (NDUFB8). mRNA.	Up_Regulated
ILMN_1681008	NM_006568.2	CGRRF1	0.38	1.30	0.0485	14	Homo sapiens cell growth regulator with ring finger domain 1 (CGRRF1). mRNA.	Up_Regulated
ILMN_1758915	NM_144781.1	PDCD2	0.38	1.30	0.0322	6	Homo sapiens programmed cell death 2 (PDCD2). transcript variant 2. mRNA.	Up_Regulated
ILMN_3237589	NM_032177.2	PHAX	0.37	1.30	0.0420	5	Homo sapiens phosphorylated adaptor for RNA export (PHAX). mRNA.	Up_Regulated
ILMN_1724181	NM_000585.2	IL15	0.37	1.30	0.0230	4	Homo sapiens interleukin 15 (IL15). transcript variant 3. mRNA.	Up_Regulated
ILMN_1761969	NM_016041.3	DERL2	0.37	1.29	0.0446	17	Homo sapiens Der1-like domain family. member 2 (DERL2). mRNA.	Up_Regulated
ILMN_1740185	NM_000367.2	TPMT	0.37	1.29	0.0432	6	Homo sapiens thiopurine S-methyltransferase (TPMT). mRNA.	Up_Regulated
ILMN_3285785	XR_039752.1	LOC647307	0.37	1.29	0.0336		PREDICTED: Homo sapiens misc_RNA (LOC647307). miscRNA.	Up_Regulated
ILMN_1699610	NM_006584.2	CCT6B	0.36	1.28	0.0319	17	Homo sapiens chaperonin containing TCP1. subunit 6B (zeta 2) (CCT6B). mRNA.	Up_Regulated
ILMN_1804955	NM_003793.2	CTSF	0.36	1.28	0.0404	11	Homo sapiens cathepsin F (CTSF). mRNA.	Up_Regulated
ILMN_2415439	NM_001018160.1	NAE1	0.36	1.28	0.0434	16	Homo sapiens NEDD8 activating enzyme E1 subunit 1 (NAE1). transcript variant 3. mRNA.	Up_Regulated
ILMN_1688034	NM_004645.2	COIL	0.35	1.28	0.0322	17	Homo sapiens coilin (COIL). mRNA.	Up_Regulated
ILMN_1763080	NM_005051.1	QARS	0.35	1.28	0.0443	3	Homo sapiens glutaminyl-tRNA synthetase (QARS). mRNA.	Up_Regulated
ILMN_1655377	NM_020191.2	MRPS22	0.35	1.27	0.0396	3	Homo sapiens mitochondrial ribosomal protein S22 (MRPS22). nuclear gene encoding mitochondrial protein. mRNA.	Up_Regulated
ILMN_1657446	NM_032324.1	C1ORF57	0.35	1.27	0.0470	1	Homo sapiens chromosome 1 open reading frame 57 (C1orf57). mRNA.	Up_Regulated
ILMN_1694100	NM_000947.2	PRIM2	0.35	1.27	0.0406	6	Homo sapiens primase. DNA. polypeptide 2 (58kDa) (PRIM2). mRNA.	Up_Regulated
ILMN_2135175	NR_002448.1	SNORD36A	0.35	1.27	0.0495	9	Homo sapiens small nucleolar RNA. C/D box 36A (SNORD36A). small nucleolar RNA.	Up_Regulated
ILMN_1789176	NM_002793.2	PSMB1	0.35	1.27	0.0441	6	Homo sapiens proteasome (prosome. macropain) subunit. beta type. 1 (PSMB1). mRNA.	Up_Regulated
ILMN_1701293	NM_001865.2	COX7A2	0.35	1.27	0.0444	6	Homo sapiens cytochrome c oxidase subunit VIIa polypeptide 2 (liver) (COX7A2). mRNA.	Up_Regulated
ILMN_1773716	NM_031420.2	MRPL9	0.35	1.27	0.0471	1	Homo sapiens mitochondrial ribosomal protein L9 (MRPL9). nuclear gene encoding mitochondrial protein. mRNA.	Up_Regulated
ILMN_3281599	XR_019077.2	LOC642741	0.34	1.27	0.0434		PREDICTED: Homo sapiens misc_RNA (LOC642741). miscRNA.	Up_Regulated
ILMN_3279712	XR_037021.1	LOC642590	0.34	1.27	0.0476		PREDICTED: Homo sapiens misc_RNA (LOC642590). miscRNA.	Up_Regulated
ILMN_1680644	NM_019005.3	MIOS	0.34	1.27	0.0407	7	Homo sapiens missing oocyte. meiosis regulator. homolog (Drosophila) (MIOS). mRNA.	Up_Regulated
ILMN_2056551	NM_016024.2	RBMX2	0.34	1.27	0.0319	X	Homo sapiens RNA binding motif protein. X-linked 2 (RBMX2). mRNA.	Up_Regulated
ILMN_3238123	NM_001136501.1	ZNF844	0.34	1.26	0.0280	19	Homo sapiens zinc finger protein 844 (ZNF844). mRNA.	Up_Regulated
ILMN_1782417	XM_944489.1	LOC651064	0.33	1.26	0.0453		PREDICTED: Homo sapiens hypothetical protein LOC651064 (LOC651064). mRNA.	Up_Regulated
ILMN_1704477	NM_004255.2	COX5A	0.33	1.26	0.0434	15	Homo sapiens cytochrome c oxidase subunit Va (COX5A). nuclear gene encoding mitochondrial protein. mRNA.	Up_Regulated
ILMN_3243871	XM_001717499.1	LOC642076	0.33	1.26	0.0491	6	PREDICTED: Homo sapiens similar to hCG1789038 (LOC642076). mRNA.	Up_Regulated
ILMN_3237579	NM_138374.1	ZNF845	0.33	1.26	0.0401	19	Homo sapiens zinc finger protein 845 (ZNF845). mRNA. XM_039908	Up_Regulated
ILMN_3284084	XR_018292.2	LOC392522	0.32	1.25	0.0299		PREDICTED: Homo sapiens misc_RNA (LOC392522). miscRNA.	Up_Regulated
ILMN_2177732	NM_012416.2	RANBP6	0.32	1.25	0.0230	9	Homo sapiens RAN binding protein 6 (RANBP6). transcript variant 1. mRNA.	Up_Regulated
ILMN_1785198	NM_017443.3	POLE3	0.32	1.25	0.0485	9	Homo sapiens polymerase (DNA directed). epsilon 3 (p17 subunit) (POLE3). mRNA.	Up_Regulated
ILMN_1784292	NM_020319.1	ANKMY2	0.32	1.25	0.0353	7	Homo sapiens ankyrin repeat and MYND domain containing 2 (ANKMY2). mRNA.	Up_Regulated
ILMN_1666477	XM_372704.4	LOC390876	0.31	1.24	0.0428	19	PREDICTED: Homo sapiens similar to 60S ribosomal protein L35 (LOC390876). mRNA.	Up_Regulated
ILMN_1757408	NM_005773.2	ZNF256	0.31	1.24	0.0441	19	Homo sapiens zinc finger protein 256 (ZNF256). mRNA.	Up_Regulated
ILMN_2288740	NM_006631.2	ZNF266	0.29	1.22	0.0429	19	Homo sapiens zinc finger protein 266 (ZNF266). mRNA.	Up_Regulated
ILMN_1788059	NM_032373.3	PCGF5	0.29	1.22	0.0319	10	Homo sapiens polycomb group ring finger 5 (PCGF5). mRNA.	Up_Regulated
ILMN_1747423	XM_931506.1	LOC389901	0.28	1.22	0.0434	X	PREDICTED: Homo sapiens similar to ATP-dependent DNA helicase II. 70 kDa subunit (Lupus Ku autoantigen protein p70)	Up_Regulated
ILMN_3307868	NM_001276.2	CHI3L1	0.28	1.21	0.0448	1	Homo sapiens chitinase 3-like 1 (cartilage glycoprotein-39) (CHI3L1). mRNA.	Up_Regulated
ILMN_2174296	NM_014377.1	DNAJC2	0.27	1.21	0.0391	7	Homo sapiens DnaJ (Hsp40) homolog. subfamily C. member 2 (DNAJC2). transcript variant 1. mRNA.	Up_Regulated
ILMN_2395055	NM_001042546.1	ATPAF1	0.26	1.20	0.0299	1	Homo sapiens ATP synthase mitochondrial F1 complex assembly factor 1 (ATPAF1). nuclear gene encoding mitochondria	Up_Regulated
ILMN_3202576	XM_001721342.1	LOC100131643	0.26	1.20	0.0443	14	PREDICTED: Homo sapiens hypothetical protein LOC100131643 (LOC100131643). mRNA.	Up_Regulated
ILMN_3294126	XR_038937.1	LOC100131160	0.26	1.19	0.0434	5	PREDICTED: Homo sapiens misc_RNA (LOC100131160). miscRNA.	Up_Regulated
ILMN_1684258	XM_940585.2	LOC646195	0.25	1.19	0.0432	11	PREDICTED: Homo sapiens similar to 40S ribosomal protein S28. transcript variant 2 (LOC646195). mRNA.	Up_Regulated
ILMN_1692133	NM_001032372.1	ZNF226	0.24	1.18	0.0434	19	Homo sapiens zinc finger protein 226 (ZNF226). transcript variant 1. mRNA.	Up_Regulated
ILMN_2115011	NM_173558.2	FGD2	0.24	1.18	0.0491	6	Homo sapiens FYVE. RhoGEF and PH domain containing 2 (FGD2). mRNA.	Up_Regulated
ILMN_2277252	NM_003622.2	PPFIBP1	0.23	1.18	0.0485	12	Homo sapiens PTPRF interacting protein. binding protein 1 (liprin beta 1) (PPFIBP1). transcript variant 1. mRNA.	Up_Regulated
ILMN_1651507	XR_015408.1	LOC642732	0.23	1.18	0.0363		PREDICTED: Homo sapiens misc_RNA (LOC642732). miscRNA.	Up_Regulated
ILMN_1802627	NM_032302.2	PSMG3	0.21	1.16	0.0470	7	Homo sapiens proteasome (prosome. macropain) assembly chaperone 3 (PSMG3). mRNA.	Up_Regulated
ILMN_1667306	NM_020850.1	RANBP10	-0.24	0.85	0.0331	16	Homo sapiens RAN binding protein 10 (RANBP10). mRNA.	Down_Regulated
ILMN_2325978	NM_001001520.1	HDGF2	-0.24	0.85	0.0469	19	Homo sapiens hepatoma-derived growth factor-related protein 2 (HDGF2). transcript variant 1. mRNA.	Down_Regulated
ILMN_1776119	NM_033450.2	ABCC10	-0.25	0.84	0.0485	6	Homo sapiens ATP-binding cassette. sub-family C (CFTR/MRP). member 10 (ABCC10). mRNA.	Down_Regulated
ILMN_1658798	NM_001080497.1	MEGF9	-0.25	0.84	0.0391	9	Homo sapiens multiple EGF-like-domains 9 (MEGF9). mRNA.	Down_Regulated
ILMN_2368617	NM_024301.3	FKRP	-0.25	0.84	0.0413	19	Homo sapiens fukutin related protein (FKRP). transcript variant 1. mRNA.	Down_Regulated
ILMN_2126239	NM_015327.1	SMG5	-0.26	0.84	0.0438	1	Homo sapiens Smg-5 homolog. nonsense mediated mRNA decay factor (C. elegans) (SMG5). mRNA.	Down_Regulated
ILMN_1763326	NM_198567.2	C5ORF25	-0.27	0.83	0.0389	5	Homo sapiens chromosome 5 open reading frame 25 (C5orf25). mRNA.	Down_Regulated
ILMN_1778132	NM_014807.3	C2CD2L	-0.28	0.82	0.0389	11	Homo sapiens C2CD2-like (C2CD2L). mRNA.	Down_Regulated
ILMN_3243943	XM_001726411.1	LOC730385	-0.28	0.82	0.0407	7	PREDICTED: Homo sapiens hypothetical LOC730385 (LOC730385). mRNA.	Down_Regulated
ILMN_1775761	NM_018128.4	TSR1	-0.28	0.82	0.0470	17	Homo sapiens TSR1. 20S rRNA accumulation. homolog (S. cerevisiae) (TSR1). mRNA.	Down_Regulated
ILMN_1725471	NM_000167.3	GK	-0.29	0.82	0.0363	X	Homo sapiens glycerol kinase (GK). transcript variant 2. mRNA.	Down_Regulated
ILMN_1691090	NM_002437.4	MPV17	-0.29	0.82	0.0437	2	Homo sapiens MpV17 mitochondrial inner membrane protein (MPV17). nuclear gene encoding mitochondrial protein. n	Down_Regulated
ILMN_1716907	NM_017727.3	FLJ20254	-0.30	0.81	0.0397	2	Homo sapiens hypothetical protein FLJ20254 (FLJ20254). mRNA.	Down_Regulated
ILMN_2131381	NM_000922.2	PDE3B	-0.30	0.81	0.0437	11	Homo sapiens phosphodiesterase 3B. cGMP-inhibited (PDE3B). mRNA.	Down_Regulated
ILMN_1781135	NM_005843.3	STAM2	-0.30	0.81	0.0484	2	Homo sapiens signal transducing adaptor molecule (SH3 domain and ITAM motif) 2 (STAM2). mRNA.	Down_Regulated
ILMN_1677768	NM_000941.2	POR	-0.31	0.81	0.0227	7	Homo sapiens P450 (cytochrome) oxidoreductase (POR). mRNA.	Down_Regulated
ILMN_1654566	NM_005527.3	HSPA1L	-0.31	0.81	0.0353	6	Homo sapiens heat shock 70kDa protein 1-like (HSPA1L). mRNA.	Down_Regulated
ILMN_2220739	NM_017905.3	TMCO3	-0.32	0.80	0.0425	13	Homo sapiens transmembrane and coiled-coil domains 3 (TMCO3). mRNA.	Down_Regulated
ILMN_2092333	NM_181791.1	GPR141	-0.32	0.80	0.0485	7	Homo sapiens G protein-coupled receptor 141 (GPR141). mRNA.	Down_Regulated
ILMN_3248412	XR_038123.1	LOC730387	-0.33	0.80	0.0414	7	PREDICTED: Homo sapiens misc_RNA (LOC730387). miscRNA.	Down_Regulated
ILMN_1754842	NM_001042486.1	DLGAP4	-0.33	0.80	0.0227	20	Homo sapiens discs. large (Drosophila) homolog-associated protein 4 (DLGAP4). transcript variant 3. mRNA.	Down_Regulated
ILMN_2319910	NM_201554.1	DGKA	-0.33	0.80	0.0429	12	Homo sapiens diacylglycerol kinase. alpha 80kDa (DGKA). transcript variant 4. mRNA.	Down_Regulated
ILMN_1673753	XM_936103.1	LOC642033	-0.33	0.80	0.0280		PREDICTED: Homo sapiens similar to ATP-binding cassette. sub-family F. member 1 isoform b (LOC642033). mRNA.	Down_Regulated
ILMN_1676759	NM_017895.6	DDX27	-0.33	0.79	0.0434	20	Homo sapiens DEAD (Asp-Glu-Ala-Asp) box polypeptide 27 (DDX27). mRNA.	Down_Regulated
ILMN_1722642	NM_001001330.1	REEP3	-0.34	0.79	0.0278	10	Homo sapiens receptor accessory protein 3 (REEP3). mRNA.	Down_Regulated
ILMN_2337551	NM_001048200.1	HIPK3	-0.34	0.79	0.0476	11	Homo sapiens homeodomain interacting protein kinase 3 (HIPK3). transcript variant 3. mRNA.	Down_Regulated
ILMN_1659470	NM_181042.2	PBRM1	-0.34	0.79	0.0434	3	Homo sapiens polybromo 1 (PBRM1). transcript variant 4. mRNA.	Down_Regulated
ILMN_1706706	NM_001003725.1	WDR68	-0.34	0.79	0.0406	17	Homo sapiens WD repeat domain 68 (WDR68). transcript variant 2. mRNA.	Down_Regulated
ILMN_1760563	NM_080686.2	BAT2	-0.35	0.78	0.0289	6	Homo sapiens HLA-B associated transcript 2 (BAT2). mRNA.	Down_Regulated
ILMN_2277419	NM_001037984.1	SLC38A10	-0.35	0.78	0.0282	17	Homo sapiens solute carrier family 38. member 10 (SLC38A10). transcript variant 1. mRNA.	Down_Regulated
ILMN_2330552	NM_033531.1	CDC2L2	-0.35	0.78	0.0448	1	Homo sapiens cell division cycle 2-like 2 (PITSLRE proteins) (CDC2L2). transcript variant 5. mRNA.	Down_Regulated
ILMN_2226015	NM_198578.2	LRRK2	-0.36	0.78	0.0319	12	Homo sapiens leucine-rich repeat kinase 2 (LRRK2). mRNA.	Down_Regulated
ILMN_2287888	NM_006503.2	PSMC4	-0.37	0.77	0.0437	19	Homo sapiens proteasome (prosome. macropain) 26S subunit. ATPase. 4 (PSMC4). transcript variant 1. mRNA.	Down_Regulated
ILMN_1795026	NM_006589.2	FAM189B	-0.37	0.77	0.0398	1	Homo sapiens family with sequence similarity 189. member B (FAM189B). transcript variant 1. mRNA.	Down_Regulated
ILMN_1698996	NM_194255.1	SLC19A1	-0.37	0.77	0.0434	21	Homo sapiens solute carrier family 19 (folate transporter). member 1 (SLC19A1). mRNA.	Down_Regulated
ILMN_1746378	NM_032484.3	GHDC	-0.38	0.77	0.0445	17	Homo sapiens GH3 domain containing (GHDC). mRNA.	Down_Regulated
ILMN_2380946	NM_001418.3	EIF4G2	-0.38	0.77	0.0292	11	Homo sapiens eukaryotic translation initiation factor 4 gamma. 2 (EIF4G2). transcript variant 1. mRNA.	Down_Regulated
ILMN_1784320	NM_014800.9	ELMO1	-0.38	0.77	0.0434	7	Homo sapiens engulfment and cell motility 1 (ELMO1). transcript variant 1. mRNA.	Down_Regulated
ILMN_1789839	NM_001520.2	GTF3C1	-0.38	0.77	0.0475	16	Homo sapiens general transcription factor IIIC. polypeptide 1. alpha 220kDa (GTF3C1). mRNA.	Down_Regulated
ILMN_1736575	NM_005762.2	TRIM28	-0.39	0.76	0.0249	19	Homo sapiens tripartite motif-containing 28 (TRIM28). mRNA.	Down_Regulated
ILMN_2197247	NM_007055.2	POLR3A	-0.39	0.76	0.0379	10	Homo sapiens polymerase (RNA) III (DNA directed) polypeptide A. 155kDa (POLR3A). mRNA.	Down_Regulated
ILMN_2294978	NM_194430.1	RNASE4	-0.39	0.76	0.0228	14	Homo sapiens ribonuclease. RNase A family. 4 (RNASE4). transcript variant 1. mRNA.	Down_Regulated
ILMN_1761828	NM_001950.3	E2F4	-0.39	0.76	0.0491	16	Homo sapiens E2F transcription factor 4. p107/p130-binding (E2F4). mRNA.	Down_Regulated

ILMN_1807833	NM_178580.1	HM13	-0.39	0.76	0.0495	20	Homo sapiens histocompatibility (minor) 13 (HM13). transcript variant 2. mRNA.	Down_Regulated
ILMN_2356574	NM_001035521.1	GTF3C2	-0.39	0.76	0.0434	2	Homo sapiens general transcription factor IIIC. polypeptide 2. beta 110kDa (GTF3C2). transcript variant 2. mRNA.	Down_Regulated
ILMN_1661409	NM_015224.2	C3ORF63	-0.39	0.76	0.0315	3	Homo sapiens chromosome 3 open reading frame 63 (C3orf63). mRNA.	Down_Regulated
ILMN_2213834	NR_002764.1	PRO0628	-0.40	0.76	0.0470	20	Homo sapiens hypothetical LOC29053 (PRO0628). non-coding RNA.	Down_Regulated
ILMN_3297644	NM_017727.4	TMEM214	-0.40	0.76	0.0227	2	Homo sapiens transmembrane protein 214 (TMEM214). transcript variant 1. mRNA.	Down_Regulated
ILMN_3255061	NM_001033555.1	CYTSB	-0.40	0.76	0.0437	17	Homo sapiens cytospin B (CYTSB). transcript variant NSP5alpha3beta. mRNA.	Down_Regulated
ILMN_1765649	NM_001571.2	IRF3	-0.40	0.76	0.0390	19	Homo sapiens interferon regulatory factor 3 (IRF3). mRNA.	Down_Regulated
ILMN_1724959	NM_014933.2	SEC31A	-0.41	0.75	0.0443	4	Homo sapiens SEC31 homolog A (S. cerevisiae) (SEC31A). transcript variant 1. mRNA.	Down_Regulated
ILMN_1680104	NM_018389.3	SLC35C1	-0.41	0.75	0.0470	11	Homo sapiens solute carrier family 35. member C1 (SLC35C1). mRNA.	Down_Regulated
ILMN_2393296	NM_203391.1	GK	-0.41	0.75	0.0324	X	Homo sapiens glycerol kinase (GK). transcript variant 1. mRNA.	Down_Regulated
ILMN_1726786	NM_015088.2	TNRC6B	-0.41	0.75	0.0406	22	Homo sapiens trinucleotide repeat containing 6B (TNRC6B). transcript variant 2. mRNA.	Down_Regulated
ILMN_2349006	NM_012475.4	USP21	-0.42	0.75	0.0227	1	Homo sapiens ubiquitin specific peptidase 21 (USP21). transcript variant 1. mRNA.	Down_Regulated
ILMN_1788315	NM_015260.1	SIN3B	-0.42	0.75	0.0404	19	Homo sapiens SIN3 homolog B. transcription regulator (yeast) (SIN3B). mRNA.	Down_Regulated
ILMN_1741032	NM_014606.1	HERC3	-0.42	0.74	0.0295	4	Homo sapiens hect domain and RLD 3 (HERC3). mRNA.	Down_Regulated
ILMN_3243069	XM_001714086.1	LOC100133692	-0.43	0.74	0.0448		PREDICTED: Homo sapiens similar to cell division cycle 2-like 1 (PITSLRE proteins) (LOC100133692). mRNA.	Down_Regulated
ILMN_1780382	XM_934796.2	LOC653566	-0.43	0.74	0.0470	1	PREDICTED: Homo sapiens similar to Signal peptidase complex subunit 2 (Microsomal signal peptidase 25 kDa subunit) (	Down_Regulated
ILMN_1652753	NM_025155.1	PAAF1	-0.43	0.74	0.0364	11	Homo sapiens proteasomal ATPase-associated factor 1 (PAAF1). mRNA.	Down_Regulated
ILMN_1655577	NM_003253.2	TIAM1	-0.43	0.74	0.0488	21	Homo sapiens T-cell lymphoma invasion and metastasis 1 (TIAM1). mRNA.	Down_Regulated
ILMN_1797342	NM_015033.2	FNBP1	-0.43	0.74	0.0407	9	Homo sapiens formin binding protein 1 (FNBP1). mRNA.	Down_Regulated
ILMN_1751034	NM_001034841.2	ITPRIPL2	-0.43	0.74	0.0398	16	Homo sapiens inositol 1.4.5-triphosphate receptor interacting protein-like 2 (ITPRIPL2). mRNA.	Down_Regulated
ILMN_3238375	NM_025082.3	CENPT	-0.44	0.74	0.0336	16	Homo sapiens centromere protein T (CENPT). mRNA.	Down_Regulated
ILMN_1765547	NM_002199.3	IRF2	-0.44	0.74	0.0286	4	Homo sapiens interferon regulatory factor 2 (IRF2). mRNA.	Down_Regulated
ILMN_1747192	NM_017831.3	RNF125	-0.44	0.74	0.0274	18	Homo sapiens ring finger protein 125 (RNF125). mRNA.	Down_Regulated
ILMN_1728517	NM_002028.3	FNTB	-0.44	0.74	0.0442	14	Homo sapiens farnesyltransferase. CAAX box. beta (FNTB). mRNA.	Down_Regulated
ILMN_1754179	NM_003917.2	AP1G2	-0.44	0.74	0.0406	14	Homo sapiens adaptor-related protein complex 1. gamma 2 subunit (AP1G2). mRNA.	Down_Regulated
ILMN_1784737	NM_003775.2	S1PR4	-0.44	0.74	0.0464	19	Homo sapiens sphingosine-1-phosphate receptor 4 (S1PR4). mRNA.	Down_Regulated
ILMN_2368318	NM_001042729.1	FGR	-0.45	0.73	0.0372	1	Homo sapiens Gardner-Rasheed feline sarcoma viral (v-fgr) oncogene homolog (FGR). transcript variant 3. mRNA.	Down_Regulated
ILMN_1789419	NM_007277.4	EXOC3	-0.45	0.73	0.0396	5	Homo sapiens exocyst complex component 3 (EXOC3). mRNA.	Down_Regulated
ILMN_1727642	NM_006302.2	MOGS	-0.45	0.73	0.0319	2	Homo sapiens mannosyl-oligosaccharide glucosidase (MOGS). transcript variant 1. mRNA.	Down_Regulated
ILMN_2072296	NM_001827.1	CKS2	-0.45	0.73	0.0434	9	Homo sapiens CDC28 protein kinase regulatory subunit 2 (CKS2). mRNA.	Down_Regulated
ILMN_1674926	NM_198532.1	C19ORF35	-0.46	0.73	0.0282	19	Homo sapiens chromosome 19 open reading frame 35 (C19orf35). mRNA.	Down_Regulated
ILMN_1786893	NM_005778.1	RBM5	-0.46	0.73	0.0469	3	Homo sapiens RNA binding motif protein 5 (RBM5). mRNA.	Down_Regulated
ILMN_3235003	XM_001715429.1	LOC100132395	-0.46	0.73	0.0227	1	PREDICTED: Homo sapiens similar to hCG1810766 (LOC100132395). mRNA.	Down_Regulated
ILMN_1668514	NM_012398.1	PIP5K1C	-0.46	0.73	0.0228	19	Homo sapiens phosphatidylinositol-4-phosphate 5-kinase. type I. gamma (PIP5K1C). mRNA.	Down_Regulated
ILMN_3269719	NM_001040118.2	ARAP1	-0.46	0.73	0.0336	11	Homo sapiens ArfGAP with RhoGAP domain. ankyrin repeat and PH domain 1 (ARAP1). transcript variant 3. mRNA.	Down_Regulated
ILMN_1810941	NM_000754.2	COMT	-0.46	0.73	0.0484	22	Homo sapiens catechol-O-methyltransferase (COMT). transcript variant MB-COMT. mRNA.	Down_Regulated
ILMN_1652680	XM_942152.1	LOC652616	-0.47	0.72	0.0227		PREDICTED: Homo sapiens similar to neutrophil cytosolic factor 1 (LOC652616). mRNA.	Down_Regulated
ILMN_1768470	NM_182917.2	EIF4G1	-0.47	0.72	0.0227	3	Homo sapiens eukaryotic translation initiation factor 4 gamma. 1 (EIF4G1). transcript variant 1. mRNA.	Down_Regulated
ILMN_1731181	NM_018469.3	TEX2	-0.47	0.72	0.0434	17	Homo sapiens testis expressed 2 (TEX2). mRNA.	Down_Regulated
ILMN_1698367	NM_003874.1	CD84	-0.48	0.72	0.0463	1	Homo sapiens CD84 molecule (CD84). mRNA.	Down_Regulated
ILMN_1799082	NM_015042.1	ZNF609	-0.48	0.72	0.0324	15	Homo sapiens zinc finger protein 609 (ZNF609). mRNA.	Down_Regulated
ILMN_2356068	NM_031267.1	CDC2L5	-0.48	0.72	0.0275	7	Homo sapiens cell division cycle 2-like 5 (cholinesterase-related cell division controller) (CDC2L5). transcript variant 2. ml	Down_Regulated
ILMN_3307752	NM_198989.2	DLEU7	-0.48	0.72	0.0466	13	Homo sapiens deleted in lymphocytic leukemia. 7 (DLEU7). mRNA.	Down_Regulated
ILMN_1751330	NM_031229.2	RBCK1	-0.48	0.72	0.0404	20	Homo sapiens RanBP-type and C3HC4-type zinc finger containing 1 (RBCK1). transcript variant 2. mRNA.	Down_Regulated
ILMN_1697559	NM_000402.3	G6PD	-0.49	0.71	0.0404	X	Homo sapiens glucose-6-phosphate dehydrogenase (G6PD). transcript variant 1. mRNA.	Down_Regulated
ILMN_1804476	NM_205847.1	GMPPA	-0.49	0.71	0.0246	2	Homo sapiens GDP-mannose pyrophosphorylase A (GMPPA). transcript variant 2. mRNA.	Down_Regulated
ILMN_1797964	NM_152522.3	ARL6IP6	-0.49	0.71	0.0437	2	Homo sapiens ADP-ribosylation-like factor 6 interacting protein 6 (ARL6IP6). mRNA.	Down_Regulated
ILMN_1751097	NM_194071.2	CREB3L2	-0.49	0.71	0.0470	7	Homo sapiens cAMP responsive element binding protein 3-like 2 (CREB3L2). mRNA.	Down_Regulated
ILMN_1718960	NM_198833.1	SERPINB8	-0.49	0.71	0.0274	18	Homo sapiens serpin peptidase inhibitor. clade B (ovalbumin). member 8 (SERPINB8). transcript variant 2. mRNA.	Down_Regulated
ILMN_1666632	NM_022553.4	VPS52	-0.49	0.71	0.0500	6	Homo sapiens vacuolar protein sorting 52 homolog (S. cerevisiae) (VPS52). mRNA.	Down_Regulated
ILMN_1744268	NM_000445.2	PLEC1	-0.50	0.71	0.0312	8	Homo sapiens plectin 1. intermediate filament binding protein 500kDa (PLEC1). transcript variant 1. mRNA.	Down_Regulated
ILMN_2064606	NM_015079.2	TBC1D2B	-0.50	0.71	0.0428	15	Homo sapiens TBC1 domain family. member 2B (TBC1D2B). mRNA.	Down_Regulated
ILMN_1741003	NM_001154.2	ANXA5	-0.50	0.71	0.0227	4	Homo sapiens annexin A5 (ANXA5). mRNA.	Down_Regulated
ILMN_1687592	NM_015691.2	WWC3	-0.50	0.71	0.0365	X	Homo sapiens WWC family member 3 (WWC3). mRNA.	Down_Regulated
ILMN_2330966	NM_173176.1	PTK2B	-0.50	0.71	0.0373	8	Homo sapiens PTK2B protein tyrosine kinase 2 beta (PTK2B). transcript variant 3. mRNA.	Down_Regulated
ILMN_2323302	NM_138927.1	SON	-0.50	0.71	0.0469	21	Homo sapiens SON DNA binding protein (SON). transcript variant f. mRNA.	Down_Regulated
ILMN_1687484	NM_003410.2	ZFX	-0.50	0.71	0.0333	X	Homo sapiens zinc finger protein. X-linked (ZFX). mRNA.	Down_Regulated
ILMN_1722294	NM_153634.2	CPNE8	-0.50	0.70	0.0280	12	Homo sapiens copine VIII (CPNE8). mRNA.	Down_Regulated
ILMN_2246661	NM_031208.1	FAHD1	-0.51	0.70	0.0434	16	Homo sapiens fumarylacetoacetate hydrolase domain containing 1 (FAHD1). transcript variant 2. mRNA.	Down_Regulated
ILMN_1657483	NM_032985.4	SEC23B	-0.51	0.70	0.0495	20	Homo sapiens Sec23 homolog B (S. cerevisiae) (SEC23B). transcript variant 2. mRNA.	Down_Regulated
ILMN_2143822	NM_021964.1	ZNF148	-0.51	0.70	0.0434	3	Homo sapiens zinc finger protein 148 (ZNF148). mRNA.	Down_Regulated
ILMN_1783350	XM_374898.4	PCNXL3	-0.51	0.70	0.0300	11	PREDICTED: Homo sapiens pecanex-like 3 (Drosophila). transcript variant 1 (PCNXL3). mRNA.	Down_Regulated
ILMN_1721116	NM_005153.2	USP10	-0.52	0.70	0.0463	16	Homo sapiens ubiquitin specific peptidase 10 (USP10). mRNA.	Down_Regulated
ILMN_3247064	NM_004814.2	SNRNP40	-0.52	0.70	0.0258	1	Homo sapiens small nuclear ribonucleoprotein 40kDa (U5) (SNRNP40). mRNA.	Down_Regulated
ILMN_1805863	NM_152348.1	WDR81	-0.52	0.70	0.0491	17	Homo sapiens WD repeat domain 81 (WDR81). mRNA.	Down_Regulated
ILMN_1665859	NM_183236.1	RAB27A	-0.52	0.70	0.0432	15	Homo sapiens RAB27A. member RAS oncogene family (RAB27A). transcript variant 4. mRNA.	Down_Regulated
ILMN_1718734	NM_005937.3	MLLT6	-0.52	0.70	0.0371	17	Homo sapiens myeloid/lymphoid or mixed-lineage leukemia (trithorax homolog. Drosophila); translocated to. 6 (MLLT6)	Down_Regulated
ILMN_1688152	NM_004843.2	IL27RA	-0.52	0.70	0.0185	19	Homo sapiens interleukin 27 receptor. alpha (IL27RA). mRNA.	Down_Regulated
ILMN_1793829	NM_019026.2	TMCO1	-0.53	0.69	0.0389	1	Homo sapiens transmembrane and coiled-coil domains 1 (TMCO1). mRNA.	Down_Regulated
ILMN_2320964	NM_015840.2	ADAR	-0.54	0.69	0.0469	1	Homo sapiens adenosine deaminase. RNA-specific (ADAR). transcript variant 2. mRNA.	Down_Regulated
ILMN_2101930	NM_002693.1	POLG	-0.54	0.69	0.0364	15	Homo sapiens polymerase (DNA directed). gamma (POLG). mRNA.	Down_Regulated
ILMN_1779677	NM_024617.2	ZCCHC6	-0.55	0.69	0.0373	9	Homo sapiens zinc finger. CCHC domain containing 6 (ZCCHC6). mRNA.	Down_Regulated
ILMN_1753393	NM_017807.2	OSGEP	-0.55	0.68	0.0282	14	Homo sapiens O-sialoglycoprotein endopeptidase (OSGEP). mRNA.	Down_Regulated
ILMN_1662232	NM_006400.3	DCTN2	-0.55	0.68	0.0327	12	Homo sapiens dynactin 2 (p50) (DCTN2). mRNA.	Down_Regulated
ILMN_1771664	NM_014358.2	CLEC4E	-0.55	0.68	0.0227	12	Homo sapiens C-type lectin domain family 4. member E (CLEC4E). mRNA.	Down_Regulated
ILMN_1784218	NM_004818.2	DDX23	-0.55	0.68	0.0473	12	Homo sapiens DEAD (Asp-Glu-Ala-Asp) box polypeptide 23 (DDX23). mRNA.	Down_Regulated
ILMN_1680579	NM_001001396.1	ATP2B4	-0.55	0.68	0.0338	1	Homo sapiens ATPase. Ca++ transporting. plasma membrane 4 (ATP2B4). transcript variant 1. mRNA.	Down_Regulated
ILMN_1699226	NM_020765.2	UBR4	-0.55	0.68	0.0445	1	Homo sapiens ubiquitin protein ligase E3 component n-recognin 4 (UBR4). mRNA.	Down_Regulated
ILMN_1661650	NM_020463.1	SMEK2	-0.55	0.68	0.0499	2	Homo sapiens SMEK homolog 2. suppressor of mek1 (Dictyostelium) (SMEK2). mRNA.	Down_Regulated
ILMN_1802027	NM_002413.3	MGST2	-0.56	0.68	0.0469	4	Homo sapiens microsomal glutathione S-transferase 2 (MGST2). mRNA.	Down_Regulated
ILMN_1703153	NM_032569.2	N-PAC	-0.56	0.68	0.0286	16	Homo sapiens cytokine-like nuclear factor n-pac (N-PAC). mRNA.	Down_Regulated
ILMN_1659058	NM_002714.2	PPP1R10	-0.56	0.68	0.0432	6	Homo sapiens protein phosphatase 1. regulatory (inhibitor) subunit 10 (PPP1R10). mRNA.	Down_Regulated
ILMN_2371379	NM_198830.1	ACLY	-0.56	0.68	0.0164	17	Homo sapiens ATP citrate lyase (ACLY). transcript variant 2. mRNA.	Down_Regulated
ILMN_1713301	NM_005137.2	DGCR2	-0.56	0.68	0.0434	22	Homo sapiens DiGeorge syndrome critical region gene 2 (DGCR2). mRNA.	Down_Regulated
ILMN_1752355	XM_166227.6	MPEG1	-0.56	0.68	0.0227	11	PREDICTED: Homo sapiens macrophage expressed gene 1. transcript variant 1 (MPEG1). mRNA.	Down_Regulated
ILMN_1683175	NM_148178.1	C9ORF23	-0.56	0.68	0.0434	9	Homo sapiens chromosome 9 open reading frame 23 (C9orf23). transcript variant 1. mRNA.	Down_Regulated
ILMN_1742544	NM_002397.2	MEF2C	-0.57	0.68	0.0241	5	Homo sapiens myocyte enhancer factor 2C (MEF2C). mRNA.	Down_Regulated
ILMN_1658460	XM_936240.1	LOC653884	-0.57	0.67	0.0227		PREDICTED: Homo sapiens similar to FUS interacting protein (serine-arginine rich) 1 (LOC653884). mRNA.	Down_Regulated
ILMN_2402806	NM_015638.2	TRPC4AP	-0.57	0.67	0.0434	20	Homo sapiens transient receptor potential cation channel. subfamily C. member 4 associated protein (TRPC4AP). transcr	Down_Regulated
ILMN_1699636	NM_014977.2	ACIN1	-0.57	0.67	0.0228	14	Homo sapiens apoptotic chromatin condensation inducer 1 (ACIN1). mRNA.	Down_Regulated
ILMN_1705679	NM_014649.1	SAFB2	-0.57	0.67	0.0278	19	Homo sapiens scaffold attachment factor B2 (SAFB2). mRNA.	Down_Regulated
ILMN_1793934	NM_014338.3	PISD	-0.57	0.67	0.0336	22	Homo sapiens phosphatidylserine decarboxylase (PISD). mRNA.	Down_Regulated
ILMN_1734483	NM_018045.5	BSDC1	-0.58	0.67	0.0227	1	Homo sapiens BSD domain containing 1 (BSDC1). mRNA.	Down_Regulated
ILMN_2054213	NM_017520.2	MPHOSPH8	-0.58	0.67	0.0432	13	Homo sapiens M-phase phosphoprotein 8 (MPHOSPH8). mRNA.	Down_Regulated
ILMN_1778360	NM_002862.3	PYGB	-0.58	0.67	0.0306	20	Homo sapiens phosphorylase. glycogen; brain (PYGB). mRNA.	Down_Regulated
ILMN_1762835	NM_014877.3	HELZ	-0.58	0.67	0.0292	17	Homo sapiens helicase with zinc finger (HELZ). mRNA.	Down_Regulated
ILMN_1714965	NM_003998.2	NFKB1	-0.59	0.67	0.0228	4	Homo sapiens nuclear factor of kappa light polypeptide gene enhancer in B-cells 1 (NFKB1). mRNA.	Down_Regulated
ILMN_1780533	NM_005615.4	RNASE6	-0.59	0.67	0.0439	14	Homo sapiens ribonuclease. RNase A family. k6 (RNASE6). mRNA.	Down_Regulated
ILMN_2367753	NM_001684.3	ATP2B4	-0.59	0.67	0.0389	1	Homo sapiens ATPase. Ca++ transporting. plasma membrane 4 (ATP2B4). transcript variant 2. mRNA.	Down_Regulated
ILMN_1743137	NM_015172.3	BAT2D1	-0.59	0.67	0.0437	1	Homo sapiens BAT2 domain containing 1 (BAT2D1). mRNA.	Down_Regulated

ILMN_1663866	NM_000358.1	TGFBI	-0.59	0.66	0.0407	5	Homo sapiens transforming growth factor. beta-induced. 68kDa (TGFBI). mRNA.	Down_Regulated
ILMN_1713402	NM_001098794.1	FAM160A2	-0.59	0.66	0.0227	11	Homo sapiens family with sequence similarity 160. member A2 (FAM160A2). transcript variant 2. mRNA.	Down_Regulated
ILMN_2208158	NR_002206.1	GTF2IP1	-0.59	0.66	0.0227	7	Homo sapiens general transcription factor II. i. pseudogene 1 (GTF2IP1) on chromosome 7.	Down_Regulated
ILMN_1742400	NM_014810.3	CEP350	-0.60	0.66	0.0227	1	Homo sapiens centrosomal protein 350kDa (CEP350). mRNA.	Down_Regulated
ILMN_1696065	NM_016547.1	SDF4	-0.60	0.66	0.0336	1	Homo sapiens stromal cell derived factor 4 (SDF4). mRNA.	Down_Regulated
ILMN_2251184	NM_006817.3	ERP29	-0.60	0.66	0.0484	12	Homo sapiens endoplasmic reticulum protein 29 (ERP29). transcript variant 1. mRNA.	Down_Regulated
ILMN_1715863	XM_001126647.1	MLKL	-0.60	0.66	0.0324		PREDICTED: Homo sapiens mixed lineage kinase domain-like (MLKL). mRNA.	Down_Regulated
ILMN_1718207	NM_012432.2	SETDB1	-0.60	0.66	0.0227	1	Homo sapiens SET domain. bifurcated 1 (SETDB1). mRNA.	Down_Regulated
ILMN_2252136	NM_006761.3	YWHAE	-0.60	0.66	0.0470	17	Homo sapiens tyrosine 3-monooxygenase/tryptophan 5-monooxygenase activation protein. epsilon polypeptide (YWHA	Down_Regulated
ILMN_1656016	NM_001357.3	DHX9	-0.60	0.66	0.0275	1	Homo sapiens DEAH (Asp-Glu-Ala-His) box polypeptide 9 (DHX9). mRNA.	Down_Regulated
ILMN_2400500	NM_022075.3	LASS2	-0.60	0.66	0.0227	1	Homo sapiens LAG1 homolog. ceramide synthase 2 (LASS2). transcript variant 2. mRNA.	Down_Regulated
ILMN_2078389	NM_003040.2	SLC4A2	-0.61	0.65	0.0227	7	Homo sapiens solute carrier family 4. anion exchanger. member 2 (erythrocyte membrane protein band 3-like 1) (SLC4A	Down_Regulated
ILMN_1704730	NM_012072.3	CD93	-0.62	0.65	0.0429	20	Homo sapiens CD93 molecule (CD93). mRNA.	Down_Regulated
ILMN_1703427	NM_138927.1	SON	-0.62	0.65	0.0470	21	Homo sapiens SON DNA binding protein (SON). transcript variant f. mRNA.	Down_Regulated
ILMN_1676600	NM_198597.1	SEC24C	-0.62	0.65	0.0466	10	Homo sapiens SEC24 family. member C (S. cerevisiae) (SEC24C). transcript variant 2. mRNA.	Down_Regulated
ILMN_1651767	NM_020831.3	MKL1	-0.63	0.65	0.0278	22	Homo sapiens megakaryoblastic leukemia (translocation) 1 (MKL1). mRNA.	Down_Regulated
ILMN_3234967	XR_038892.1	LOC646301	-0.63	0.64	0.0336	17	PREDICTED: Homo sapiens misc_RNA (LOC646301). miscRNA.	Down_Regulated
ILMN_2117623	NM_005761.1	PLXNC1	-0.64	0.64	0.0398	12	Homo sapiens plexin C1 (PLXNC1). mRNA.	Down_Regulated
ILMN_1660810	NM_018259.4	TTC17	-0.64	0.64	0.0312	11	Homo sapiens tetratricopeptide repeat domain 17 (TTC17). mRNA.	Down_Regulated
ILMN_1772686	NM_033086.2	FGD3	-0.65	0.64	0.0434	9	Homo sapiens FYVE. RhoGEF and PH domain containing 3 (FGD3). transcript variant 2. mRNA.	Down_Regulated
ILMN_1733276	NM_006639.2	CYSLTR1	-0.65	0.64	0.0286	X	Homo sapiens cysteinyl leukotriene receptor 1 (CYSLTR1). mRNA.	Down_Regulated
ILMN_2075334	NM_003542.3	HIST1H4C	-0.65	0.64	0.0332	6	Homo sapiens histone cluster 1. H4c (HIST1H4C). mRNA.	Down_Regulated
ILMN_1727284	NM_000616.3	CD4	-0.65	0.64	0.0171	12	Homo sapiens CD4 molecule (CD4). mRNA.	Down_Regulated
ILMN_1713156	NM_078629.1	MSL3L1	-0.65	0.64	0.0309	X	Homo sapiens male-specific lethal 3-like 1 (Drosophila) (MSL3L1). transcript variant 1. mRNA.	Down_Regulated
ILMN_1804863	NM_015258.1	FKBP15	-0.65	0.64	0.0299	9	Homo sapiens FK506 binding protein 15. 133kDa (FKBP15). mRNA.	Down_Regulated
ILMN_1785988	NM_001025105.1	CSNK1A1	-0.66	0.63	0.0227	5	Homo sapiens casein kinase 1. alpha 1 (CSNK1A1). transcript variant 1. mRNA.	Down_Regulated
ILMN_3238001	XR_037182.1	LOC100132112	-0.66	0.63	0.0396	7	PREDICTED: Homo sapiens misc_RNA (LOC100132112). partial miscRNA.	Down_Regulated
ILMN_1721204	XM_942501.1	CSF2RA	-0.66	0.63	0.0227		PREDICTED: Homo sapiens colony stimulating factor 2 receptor. alpha. low-affinity (granulocyte-macrophage) (CSF2RA).	Down_Regulated
ILMN_2283325	NM_024911.4	GPR177	-0.66	0.63	0.0249	1	Homo sapiens G protein-coupled receptor 177 (GPR177). transcript variant 1. mRNA.	Down_Regulated
ILMN_1806122	NM_020920.2	CHD8	-0.66	0.63	0.0319	14	Homo sapiens chromodomain helicase DNA binding protein 8 (CHD8). mRNA.	Down_Regulated
ILMN_1693311	NM_003217.2	TMBIM6	-0.66	0.63	0.0164	12	Homo sapiens transmembrane BAX inhibitor motif containing 6 (TMBIM6). transcript variant 1. mRNA.	Down_Regulated
ILMN_2398865	NM_018080.2	VPS13C	-0.66	0.63	0.0322	15	Homo sapiens vacuolar protein sorting 13 homolog C (S. cerevisiae) (VPS13C). transcript variant 1B. mRNA.	Down_Regulated
ILMN_1666933	NM_004674.2	ASH2L	-0.67	0.63	0.0444		Homo sapiens ash2 (absent. small. or homeotic)-like (Drosophila) (ASH2L). mRNA.	Down_Regulated
ILMN_2154115	NM_012455.2	PSD4	-0.67	0.63	0.0324	2	Homo sapiens pleckstrin and Sec7 domain containing 4 (PSD4). mRNA.	Down_Regulated
ILMN_2228809	NM_012479.2	YWHAG	-0.67	0.63	0.0489	7	Homo sapiens tyrosine 3-monooxygenase/tryptophan 5-monooxygenase activation protein. gamma polypeptide (YWHA	Down_Regulated
ILMN_3225300	XR_015428.2	LOC728532	-0.67	0.63	0.0484	10	PREDICTED: Homo sapiens misc_RNA (LOC728532). miscRNA.	Down_Regulated
ILMN_2069446	NM_014649.2	SAFB2	-0.68	0.63	0.0434	19	Homo sapiens scaffold attachment factor B2 (SAFB2). mRNA.	Down_Regulated
ILMN_1756131	NM_194310.1	LOC284837	-0.68	0.63	0.0446	21	Homo sapiens hypothetical protein LOC284837 (LOC284837). mRNA.	Down_Regulated
ILMN_2279961	NM_013995.1	LAMP2	-0.68	0.62	0.0370	X	Homo sapiens lysosomal-associated membrane protein 2 (LAMP2). transcript variant LAMP2B. mRNA.	Down_Regulated
ILMN_2083469	NM_003749.2	IRS2	-0.68	0.62	0.0336	13	Homo sapiens insulin receptor substrate 2 (IRS2). mRNA.	Down_Regulated
ILMN_1655557	NM_001039937.1	INTS6	-0.68	0.62	0.0432	13	Homo sapiens integrator complex subunit 6 (INTS6). transcript variant 2. mRNA.	Down_Regulated
ILMN_2358540	NM_002897.3	RBMS1	-0.68	0.62	0.0324	2	Homo sapiens RNA binding motif. single stranded interacting protein 1 (RBMS1). transcript variant 3. mRNA.	Down_Regulated
ILMN_1672503	NM_001386.4	DPYSL2	-0.68	0.62	0.0434	8	Homo sapiens dihydropyrimidinase-like 2 (DPYSL2). mRNA.	Down_Regulated
ILMN_1724407	NM_006342.1	TACC3	-0.68	0.62	0.0096	4	Homo sapiens transforming. acidic coiled-coil containing protein 3 (TACC3). mRNA.	Down_Regulated
ILMN_1778374	NM_198590.1	BSG	-0.69	0.62	0.0466	19	Homo sapiens basigin (Ok blood group) (BSG). transcript variant 3. mRNA.	Down_Regulated
ILMN_1700923	NM_032852.2	ATG4C	-0.69	0.62	0.0476	1	Homo sapiens ATG4 autophagy related 4 homolog C (S. cerevisiae) (ATG4C). transcript variant 7. mRNA.	Down_Regulated
ILMN_1758864	NM_004665.2	VNN2	-0.69	0.62	0.0401	6	Homo sapiens vanin 2 (VNN2). transcript variant 1. mRNA.	Down_Regulated
ILMN_1737535	NM_003185.3	TAF4	-0.69	0.62	0.0467	20	Homo sapiens TAF4 RNA polymerase II. TATA box binding protein (TBP)-associated factor. 135kDa (TAF4). mRNA.	Down_Regulated
ILMN_1663080	NM_001040167.1	LFNG	-0.69	0.62	0.0437	7	Homo sapiens LFNG O-fucosylpeptide 3-beta-N-acetylglucosaminyltransferase (LFNG). transcript variant 1. mRNA.	Down_Regulated
ILMN_1704972	NM_033034.1	TRIM5	-0.70	0.62	0.0331	11	Homo sapiens tripartite motif-containing 5 (TRIM5). transcript variant alpha. mRNA.	Down_Regulated
ILMN_1751164	NM_001025598.1	ARHGAP30	-0.70	0.62	0.0336	1	Homo sapiens Rho GTPase activating protein 30 (ARHGAP30). transcript variant 1. mRNA.	Down_Regulated
ILMN_1708414	NM_019067.4	GNL3L	-0.70	0.62	0.0227	X	Homo sapiens guanine nucleotide binding protein-like 3 (nucleolar)-like (GNL3L). mRNA.	Down_Regulated
ILMN_1772370	NM_004706.3	ARHGEF1	-0.70	0.62	0.0371	19	Homo sapiens Rho guanine nucleotide exchange factor (GEF) 1 (ARHGEF1). transcript variant 2. mRNA.	Down_Regulated
ILMN_1790689	NM_031476.2	CRISPLD2	-0.70	0.61	0.0227	16	Homo sapiens cysteine-rich secretory protein LCCL domain containing 2 (CRISPLD2). mRNA.	Down_Regulated
ILMN_1768488	NM_005652.2	TERF2	-0.70	0.61	0.0164	16	Homo sapiens telomeric repeat binding factor 2 (TERF2). mRNA.	Down_Regulated
ILMN_1801710	NM_019043.3	APBB1IP	-0.71	0.61	0.0258	10	Homo sapiens amyloid beta (A4) precursor protein-binding. family B. member 1 interacting protein (APBB1IP). mRNA.	Down_Regulated
ILMN_1783684	XM_944246.2	LOC648695	-0.72	0.61	0.0364		PREDICTED: Homo sapiens similar to retinoblastoma binding protein 4. transcript variant 5 (LOC648695). mRNA.	Down_Regulated
ILMN_1676575	NM_006060.3	IKZF1	-0.73	0.60	0.0495	7	Homo sapiens IKAROS family zinc finger 1 (Ikaros) (IKZF1). mRNA.	Down_Regulated
ILMN_1731001	NM_207332.1	ERICH1	-0.73	0.60	0.0182	8	Homo sapiens glutamate-rich 1 (ERICH1). mRNA.	Down_Regulated
ILMN_1737498	XM_001126647.1	MLKL	-0.73	0.60	0.0172		PREDICTED: Homo sapiens mixed lineage kinase domain-like (MLKL). mRNA.	Down_Regulated
ILMN_1696432	NM_005896.2	IDH1	-0.73	0.60	0.0485	2	Homo sapiens isocitrate dehydrogenase 1 (NADP+). soluble (IDH1). mRNA.	Down_Regulated
ILMN_1781207	NM_153047.1	FYN	-0.73	0.60	0.0164	6	Homo sapiens FYN oncogene related to SRC. FGR. YES (FYN). transcript variant 2. mRNA.	Down_Regulated
ILMN_1742052	NM_004155.3	SERPINB9	-0.74	0.60	0.0331	6	Homo sapiens serpin peptidase inhibitor. clade B (ovalbumin). member 9 (SERPINB9). mRNA.	Down_Regulated
ILMN_1685371	NM_001042470.1	SUMF2	-0.74	0.60	0.0227	7	Homo sapiens sulfatase modifying factor 2 (SUMF2). transcript variant 4. mRNA.	Down_Regulated
ILMN_1789830	NM_003879.3	CFLAR	-0.75	0.60	0.0397	2	Homo sapiens CASP8 and FADD-like apoptosis regulator (CFLAR). mRNA.	Down_Regulated
ILMN_3249327	XM_001714618.1	LOC100134530	-0.75	0.60	0.0348	7	PREDICTED: Homo sapiens hypothetical protein LOC100134530 (LOC100134530). mRNA.	Down_Regulated
ILMN_2103362	NM_199282.1	ARHGAP27	-0.75	0.59	0.0173	17	Homo sapiens Rho GTPase activating protein 27 (ARHGAP27). mRNA.	Down_Regulated
ILMN_1770673	NM_030767.3	AKNA	-0.75	0.59	0.0389		Homo sapiens AT-hook transcription factor (AKNA). mRNA.	Down_Regulated
ILMN_1672589	NM_198925.1	SEMA4B	-0.76	0.59	0.0227	15	Homo sapiens sema domain. immunoglobulin domain (Ig). transmembrane domain (TM) and short cytoplasmic domain	Down_Regulated
ILMN_1758798	XM_001132754.1	LOC728734	-0.76	0.59	0.0164	16	PREDICTED: Homo sapiens similar to kidney-specific protein (KS). transcript variant 1 (LOC728734). mRNA.	Down_Regulated
ILMN_1734543	NM_130435.2	PTPRE	-0.76	0.59	0.0389	10	Homo sapiens protein tyrosine phosphatase. receptor type. E (PTPRE). transcript variant 2. mRNA.	Down_Regulated
ILMN_3251658	NM_024318.2	LILRA6	-0.77	0.59	0.0315	19	Homo sapiens leukocyte immunoglobulin-like receptor. subfamily A (with TM domain). member 6 (LILRA6). mRNA.	Down_Regulated
ILMN_1662334	NM_005147.3	DNAJA3	-0.77	0.59	0.0429	16	Homo sapiens DnaJ (Hsp40) homolog. subfamily A. member 3 (DNAJA3). mRNA.	Down_Regulated
ILMN_1693650	NM_002005.2	FES	-0.77	0.59	0.0322	15	Homo sapiens feline sarcoma oncogene (FES). mRNA.	Down_Regulated
ILMN_1753608	NM_015348.1	TMEM131	-0.77	0.58	0.0470	2	Homo sapiens transmembrane protein 131 (TMEM131). mRNA.	Down_Regulated
ILMN_1698605	NM_024334.1	TMEM43	-0.77	0.58	0.0227	3	Homo sapiens transmembrane protein 43 (TMEM43). mRNA.	Down_Regulated
ILMN_1653652	NM_080923.2	PTPRC	-0.78	0.58	0.0484	1	Homo sapiens protein tyrosine phosphatase. receptor type. C (PTPRC). transcript variant 4. mRNA.	Down_Regulated
ILMN_1683609	NM_003334.2	UBE1	-0.78	0.58	0.0227	X	Homo sapiens ubiquitin-activating enzyme E1 (UBE1). transcript variant 1. mRNA.	Down_Regulated
ILMN_1752520	NM_152270.2	SLFN11	-0.78	0.58	0.0434	17	Homo sapiens schlafen family member 11 (SLFN11). mRNA.	Down_Regulated
ILMN_3241234	XM_001126471.1	LOC730278	-0.78	0.58	0.0336	7	PREDICTED: Homo sapiens hypothetical LOC730278 (LOC730278). mRNA.	Down_Regulated
ILMN_2104696	NM_207332.1	ERICH1	-0.79	0.58	0.0286	8	Homo sapiens glutamate-rich 1 (ERICH1). mRNA.	Down_Regulated
ILMN_1679428	NM_012110.2	CHIC2	-0.79	0.58	0.0164	4	Homo sapiens cysteine-rich hydrophobic domain 2 (CHIC2). mRNA.	Down_Regulated
ILMN_2340217	NM_080921.2	PTPRC	-0.79	0.58	0.0299	1	Homo sapiens protein tyrosine phosphatase. receptor type. C (PTPRC). transcript variant 2. mRNA.	Down_Regulated
ILMN_1666089	NM_138983.1	OLIG1	-0.79	0.58	0.0449	21	Homo sapiens oligodendrocyte transcription factor 1 (OLIG1). mRNA.	Down_Regulated
ILMN_1767377	NM_207331.2	LOC153561	-0.79	0.58	0.0485	5	Homo sapiens hypothetical protein LOC153561 (LOC153561). mRNA.	Down_Regulated
ILMN_2410986	NM_139276.2	STAT3	-0.81	0.57	0.0437	17	Homo sapiens signal transducer and activator of transcription 3 (acute-phase response factor) (STAT3). transcript varian	Down_Regulated
ILMN_1761721	NM_018206.3	VPS35	-0.81	0.57	0.0401	16	Homo sapiens vacuolar protein sorting 35 homolog (S. cerevisiae) (VPS35). mRNA.	Down_Regulated
ILMN_1760727	NM_001097577.1	ANG	-0.81	0.57	0.0227	14	Homo sapiens angiogenin. ribonuclease. RNase A family. 5 (ANG). transcript variant 2. mRNA.	Down_Regulated
ILMN_1700584	NM_004907.2	IER2	-0.81	0.57	0.0432	19	Homo sapiens immediate early response 2 (IER2). mRNA.	Down_Regulated
ILMN_2087692	NM_024843.2	CYBRD1	-0.81	0.57	0.0401	2	Homo sapiens cytochrome b reductase 1 (CYBRD1). mRNA.	Down_Regulated
ILMN_2326953	NM_032464.2	LAT2	-0.81	0.57	0.0227	7	Homo sapiens linker for activation of T cells family. member 2 (LAT2). transcript variant 1. mRNA.	Down_Regulated
ILMN_1752351	NM_013995.1	LAMP2	-0.82	0.57	0.0227	X	Homo sapiens lysosomal-associated membrane protein 2 (LAMP2). transcript variant LAMP2B. mRNA.	Down_Regulated
ILMN_1672650	NM_002654.3	PKM2	-0.82	0.57	0.0353	15	Homo sapiens pyruvate kinase. muscle (PKM2). transcript variant 1. mRNA.	Down_Regulated
ILMN_1671703	NM_001613.1	ACTA2	-0.82	0.57	0.0429	10	Homo sapiens actin. alpha 2. smooth muscle. aorta (ACTA2). mRNA.	Down_Regulated
ILMN_1684385	NM_004766.1	COPB2	-0.82	0.57	0.0288	3	Homo sapiens coatomer protein complex. subunit beta 2 (beta prime) (COPB2). mRNA.	Down_Regulated
ILMN_1795835	XM_931359.2	LOC338758	-0.82	0.57	0.0396	12	PREDICTED: Homo sapiens hypothetical protein LOC338758 (LOC338758). mRNA.	Down_Regulated
ILMN_1750518	XM_001134346.1	THOC4	-0.84	0.56	0.0275		PREDICTED: Homo sapiens THO complex 4 (THOC4). mRNA.	Down_Regulated
ILMN_1794470	NM_016376.3	ANKFY1	-0.84	0.56	0.0404	17	Homo sapiens ankyrin repeat and FYVE domain containing 1 (ANKFY1). transcript variant 1. mRNA.	Down_Regulated
ILMN_1671281	NM_021133.2	RNASEL	-0.84	0.56	0.0331	1	Homo sapiens ribonuclease L (2'.5'-oligoisoadenylate synthetase-dependent) (RNASEL). mRNA.	Down_Regulated
ILMN_1790807	NM_004628.3	XPC	-0.85	0.56	0.0182	3	Homo sapiens xeroderma pigmentosum. complementation group C (XPC). mRNA.	Down_Regulated

ILMN_1776464	NM_006437.3	PARP4	-0.85	0.56	0.0321	13	Homo sapiens poly (ADP-ribose) polymerase family. member 4 (PARP4). mRNA.	Down_Regulated
ILMN_2383611	NM_130435.2	PTPRE	-0.85	0.55	0.0322	10	Homo sapiens protein tyrosine phosphatase. receptor type. E (PTPRE). transcript variant 2. mRNA.	Down_Regulated
ILMN_1759326	NM_002562.4	P2RX7	-0.85	0.55	0.0164	12	Homo sapiens purinergic receptor P2X. ligand-gated ion channel. 7 (P2RX7). mRNA.	Down_Regulated
ILMN_3251545	NM_016410.4	CHMP5	-0.86	0.55	0.0389	9	Homo sapiens chromatin modifying protein 5 (CHMP5). mRNA.	Down_Regulated
ILMN_3297945	NM_003334.3	UBA1	-0.87	0.55	0.0227	X	Homo sapiens ubiquitin-like modifier activating enzyme 1 (UBA1). transcript variant 1. mRNA.	Down_Regulated
ILMN_1756806	NM_021960.3	MCL1	-0.87	0.55	0.0444	1	Homo sapiens myeloid cell leukemia sequence 1 (BCL2-related) (MCL1). transcript variant 1. mRNA.	Down_Regulated
ILMN_1784661	NM_013390.1	TMEM2	-0.88	0.54	0.0254	9	Homo sapiens transmembrane protein 2 (TMEM2). mRNA.	Down_Regulated
ILMN_3305772	XR_038094.1	LOC730286	-0.88	0.54	0.0442	7	PREDICTED: Homo sapiens misc_RNA (LOC730286). miscRNA.	Down_Regulated
ILMN_1739210	NM_001042549.1	NSL1	-0.89	0.54	0.0336	1	Homo sapiens NSL1. MIND kinetochore complex component. homolog (S. cerevisiae) (NSL1). transcript variant 2. mRNA.	Down_Regulated
ILMN_1658247	NM_002534.2	OAS1	-0.89	0.54	0.0444	12	Homo sapiens 2'.5'-oligoadenylate synthetase 1. 40/46kDa (OAS1). transcript variant 2. mRNA.	Down_Regulated
ILMN_1710937	NM_005531.1	IFI16	-0.90	0.53	0.0324	1	Homo sapiens interferon. gamma-inducible protein 16 (IFI16). mRNA.	Down_Regulated
ILMN_3246910	NR_024456.1	LOC100190986	-0.90	0.53	0.0230	16	Homo sapiens hypothetical LOC100190986 (LOC100190986). non-coding RNA.	Down_Regulated
ILMN_2087575	NM_015168.1	ZC3H4	-0.92	0.53	0.0407	19	Homo sapiens zinc finger CCCH-type containing 4 (ZC3H4). mRNA.	Down_Regulated
ILMN_1701753	XR_016547.1	LOC644063	-0.92	0.53	0.0353	3	PREDICTED: Homo sapiens similar to heterogeneous nuclear ribonucleoprotein K (LOC644063). mRNA.	Down_Regulated
ILMN_1699703	NM_001655.3	ARCN1	-0.93	0.52	0.0227	11	Homo sapiens archain 1 (ARCN1). mRNA.	Down_Regulated
ILMN_1797731	NM_022349.2	MS4A6A	-0.94	0.52	0.0272	11	Homo sapiens membrane-spanning 4-domains. subfamily A. member 6A (MS4A6A). transcript variant 2. mRNA.	Down_Regulated
ILMN_1788213	NM_012083.2	FRAT2	-0.94	0.52	0.0465	10	Homo sapiens frequently rearranged in advanced T-cell lymphomas 2 (FRAT2). mRNA.	Down_Regulated
ILMN_1660579	NM_015986.2	CRLF3	-0.95	0.52	0.0228	17	Homo sapiens cytokine receptor-like factor 3 (CRLF3). mRNA.	Down_Regulated
ILMN_1683658	NM_000801.2	FKBP1A	-0.95	0.52	0.0299	20	Homo sapiens FK506 binding protein 1A. 12kDa (FKBP1A). transcript variant 12B. mRNA.	Down_Regulated
ILMN_1654778	NM_001013253.1	LSP1	-0.96	0.52	0.0444	11	Homo sapiens lymphocyte-specific protein 1 (LSP1). transcript variant 2. mRNA.	Down_Regulated
ILMN_1659463	NM_013229.2	APAF1	-0.96	0.51	0.0327	12	Homo sapiens apoptotic peptidase activating factor 1 (APAF1). transcript variant 1. mRNA.	Down_Regulated
ILMN_1751887	NM_002726.3	PREP	-0.97	0.51	0.0228	6	Homo sapiens prolyl endopeptidase (PREP). mRNA.	Down_Regulated
ILMN_1802205	NM_004040.2	RHOB	-0.97	0.51	0.0245	2	Homo sapiens ras homolog gene family. member B (RHOB). mRNA.	Down_Regulated
ILMN_1669674	NM_006586.3	CNPY3	-0.97	0.51	0.0230	6	Homo sapiens canopy 3 homolog (zebrafish) (CNPY3). mRNA.	Down_Regulated
ILMN_1781468	NM_022733.1	SMAP2	-0.98	0.51	0.0227	1	Homo sapiens small ArfGAP2 (SMAP2). mRNA.	Down_Regulated
ILMN_1814789	NM_014847.2	UBAP2L	-0.99	0.50	0.0434	1	Homo sapiens ubiquitin associated protein 2-like (UBAP2L). mRNA.	Down_Regulated
ILMN_2159453	NM_006949.1	STXBP2	-0.99	0.50	0.0227	19	Homo sapiens syntaxin binding protein 2 (STXBP2). mRNA.	Down_Regulated
ILMN_1724250	NM_002087.2	GRN	-1.03	0.49	0.0229	17	Homo sapiens granulin (GRN). mRNA.	Down_Regulated
ILMN_1787529	NM_004054.2	C3AR1	-1.03	0.49	0.0401	12	Homo sapiens complement component 3a receptor 1 (C3AR1). mRNA.	Down_Regulated
ILMN_1763837	NM_001150.1	ANPEP	-1.03	0.49	0.0286	15	Homo sapiens alanyl (membrane) aminopeptidase (aminopeptidase N. aminopeptidase M. microsomal aminopeptidase	Down_Regulated
ILMN_1685625	NM_003355.2	UCP2	-1.04	0.48	0.0441	11	Homo sapiens uncoupling protein 2 (mitochondrial. proton carrier) (UCP2). nuclear gene encoding mitochondrial protein	Down_Regulated
ILMN_1713749	NM_007074.2	CORO1A	-1.05	0.48	0.0339	16	Homo sapiens coronin. actin binding protein. 1A (CORO1A). mRNA.	Down_Regulated
ILMN_1757072	XM_925989.1	LOC642489	-1.05	0.48	0.0164	6	PREDICTED: Homo sapiens similar to FK506-binding protein 1A (LOC642489). mRNA.	Down_Regulated
ILMN_1665235	NM_006371.3	CRTAP	-1.07	0.48	0.0227	3	Homo sapiens cartilage associated protein (CRTAP). mRNA.	Down_Regulated
ILMN_1736700	NM_184041.1	ALDOA	-1.08	0.47	0.0404	16	Homo sapiens aldolase A. fructose-bisphosphate (ALDOA). transcript variant 2. mRNA.	Down_Regulated
ILMN_1770085	NM_006763.2	BTG2	-1.09	0.47	0.0227	1	Homo sapiens BTG family. member 2 (BTG2). mRNA.	Down_Regulated
ILMN_2356838	NM_006090.3	CEPT1	-1.10	0.47	0.0227	1	Homo sapiens choline/ethanolamine phosphotransferase 1 (CEPT1). transcript variant 1. mRNA.	Down_Regulated
ILMN_2112988	NR_003187.1	NCF1C	-1.10	0.47	0.0215	7	Homo sapiens neutrophil cytosolic factor 1C pseudogene (NCF1C). non-coding RNA.	Down_Regulated
ILMN_1746090	NM_152713.2	STT3A	-1.12	0.46	0.0164	11	Homo sapiens STT3. subunit of the oligosaccharyltransferase complex. homolog A (S. cerevisiae) (STT3A). mRNA.	Down_Regulated
ILMN_1738992	NM_002432.1	MNDA	-1.12	0.46	0.0348	1	Homo sapiens myeloid cell nuclear differentiation antigen (MNDA). mRNA.	Down_Regulated
ILMN_1758087	NM_020791.1	TAOK1	-1.21	0.43	0.0243	17	Homo sapiens TAO kinase 1 (TAOK1). mRNA.	Down_Regulated
ILMN_2179837	NM_003860.2	BANF1	-1.22	0.43	0.0164	11	Homo sapiens barrier to autointegration factor 1 (BANF1). mRNA.	Down_Regulated
ILMN_1668960	NM_021242.4	MID1IP1	-1.22	0.43	0.0227	X	Homo sapiens MID1 interacting protein 1 (gastrulation specific G12 homolog (zebrafish)) (MID1IP1). transcript variant 1.	Down_Regulated
ILMN_1743646	NM_003370.3	VASP	-1.27	0.42	0.0227	19	Homo sapiens vasodilator-stimulated phosphoprotein (VASP). mRNA.	Down_Regulated
ILMN_1716105	NM_144687.1	NLRP12	-1.28	0.41	0.0210	19	Homo sapiens NLR family. pyrin domain containing 12 (NLRP12). transcript variant 2. mRNA.	Down_Regulated
ILMN_1676588	NM_001007794.1	CEPT1	-1.28	0.41	0.0221	1	Homo sapiens choline/ethanolamine phosphotransferase 1 (CEPT1). transcript variant 2. mRNA.	Down_Regulated
ILMN_2375825	NM_001774.2	CD37	-1.30	0.41	0.0227	19	Homo sapiens CD37 molecule (CD37). transcript variant 1. mRNA.	Down_Regulated
ILMN_1697309	NM_000265.4	NCF1	-1.32	0.40	0.0164	7	Homo sapiens neutrophil cytosolic factor 1 (NCF1). mRNA.	Down_Regulated
ILMN_1786176	NM_001774.1	CD37	-1.32	0.40	0.0249	19	Homo sapiens CD37 antigen (CD37). mRNA.	Down_Regulated
ILMN_3289090	XR_015606.1	LOC728059	-1.38	0.38	0.0295		PREDICTED: Homo sapiens misc_RNA (LOC728059). miscRNA.	Down_Regulated
ILMN_2155719	NM_001039703.1	NBPF10	-1.38	0.38	0.0324		Homo sapiens neuroblastoma breakpoint family. member 10 (NBPF10). mRNA. XM_930727 XM_930739 XM_930751 XM	Down_Regulated
ILMN_2208903	NM_001803.2	CD52	-1.50	0.35	0.0292	1	Homo sapiens CD52 molecule (CD52). mRNA.	Down_Regulated
ILMN_2115490	NM_001037675.1	NBPF20	-1.52	0.35	0.0227	1	Homo sapiens neuroblastoma breakpoint family. member 20 (NBPF20). mRNA.	Down_Regulated
ILMN_2086077	NM_002229.2	JUNB	-1.54	0.34	0.0227	19	Homo sapiens jun B proto-oncogene (JUNB). mRNA.	Down_Regulated
ILMN_1762899	NM_001964.2	EGR1	-1.63	0.32	0.0469	5	Homo sapiens early growth response 1 (EGR1). mRNA.	Down_Regulated
ILMN_1704961	NM_001614.2	ACTG1	-1.63	0.32	0.0227	17	Homo sapiens actin. gamma 1 (ACTG1). mRNA.	Down_Regulated
ILMN_1789074	NM_005345.4	HSPA1A	-1.69	0.31	0.0336	6	Homo sapiens heat shock 70kDa protein 1A (HSPA1A). mRNA.	Down_Regulated
ILMN_3246805	XM_001713810.1	LOC100134364	-1.91	0.27	0.0398		PREDICTED: Homo sapiens hypothetical protein LOC100134364 (LOC100134364). mRNA.	Down_Regulated
ILMN_1733559	NR_003287.1	LOC100008589	-2.12	0.23	0.0407		Homo sapiens 28S ribosomal RNA (LOC100008589). non-coding RNA.	Down_Regulated
ILMN_3249578	XM_001713809.1	LOC100132394	-2.21	0.22	0.0373	X	PREDICTED: Homo sapiens hypothetical protein LOC100132394 (LOC100132394). mRNA.	Down_Regulated
ILMN_1682312	NM_000397.2	CYBB	-2.22	0.22	0.0182	X	Homo sapiens cytochrome b-245. beta polypeptide (chronic granulomatous disease) (CYBB). mRNA.	Down_Regulated
ILMN_3251587	NR_003287.1	LOC100008589	-2.32	0.20	0.0327		Homo sapiens 28S ribosomal RNA (LOC100008589). non-coding RNA.	Down_Regulated
